# Synthesis and Biological Evaluation of New Quinoline and Anthranilic Acid Derivatives as Potential Quorum Sensing Inhibitors

**DOI:** 10.3390/molecules28155866

**Published:** 2023-08-03

**Authors:** Ivana Perković, Tanja Poljak, Kirsi Savijoki, Pekka Varmanen, Gordana Maravić-Vlahoviček, Maja Beus, Anja Kučević, Ivan Džajić, Zrinka Rajić

**Affiliations:** 1 Faculty of Pharmacy and Biochemistry, University of Zagreb, 10000 Zagreb, Croatia; gmaravic@pharma.hr (G.M.-V.); mbeus@pharma.hr (M.B.); anja.kucevic@proton.me (A.K.); zrajic@pharma.hr (Z.R.); 2 Selvita Ltd., 10000 Zagreb, Croatia; tanja.poljak@selvita.com; 3 Department of Food and Nutrition, Faculty of Agriculture and Forestry, University of Helsinki, 00014 Helsinki, Finland; pekka.varmanen@helsinki.fi; 4 Faculty of Pharmacy, University of Ljubljana, 1000 Ljubljana, Slovenia; ivan.dzajic@ffa.uni-lj.si

**Keywords:** 4-amino-7-chloroquinoline, 1,3,4-oxadiazole, anthranilic acid, synthesis, quorum sensing, antibiofilm/virulence, *Chromobacterium violaceum*, *Pseudomonas aeruginosa*, PQS

## Abstract

Inhibiting quorum sensing (QS), a central communication system, is a promising strategy to combat bacterial pathogens without antibiotics. Here, we designed novel hybrid compounds targeting the PQS (*Pseudomonas* quinolone signal)-dependent quorum sensing (QS) of *Pseudomonas aeruginosa* that is one of the multidrug-resistant and highly virulent pathogens with urgent need of new antibacterial strategies. We synthesized 12 compounds using standard procedures to combine halogen-substituted anthranilic acids with 4-(2-aminoethyl/4-aminobuthyl)amino-7-chloroquinoline, linked via 1,3,4-oxadiazole. Their antibiofilm activities were first pre-screened using Gram-negative *Chromobacterium violaceum*-based reporter, which identified compounds **15**–**19** and **23** with the highest anti-QS and minimal bactericidal effects in a single experiment. These five compounds were then evaluated against *P. aeruginosa* PAO1 to assess their ability to prevent biofilm formation, eradicate pre-formed biofilms, and inhibit virulence using pyocyanin as a representative marker. Compound **15** displayed the most potent antibiofilm effect, reducing biofilm formation by nearly 50% and pre-formed biofilm masses by 25%. On the other hand, compound **23** exhibited the most significant antivirulence effect, reducing pyocyanin synthesis by over 70%. Thus, our study highlights the potential of 1,3,4-oxadiazoles **15** and **23** as promising scaffolds to combat *P. aeruginosa*. Additionally, interactive QS systems should be considered to achieve maximal anti-QS activity against this clinically relevant species.

## 1. Introduction

Bacterial pathogens are equipped with sophisticated strategies to establish disease and overcome obstacles to increase viability in the host and against antibiotics. Antibiotics effectively target and eliminate specific bacteria; however, their use also exerts selective pressure on bacterial cells, resulting in the emergence of resistant species. Consequently, this poses challenges in achieving a successful therapeutic outcome for the infection [1]. Based on the data collected from 204 countries worldwide, an estimated 4.95 million deaths were associated with bacterial antimicrobial resistance (AMR) in 2019, including 1.27 million deaths directly attributable to bacterial AMR [2]. The decline in antibacterial drug discovery and development together with the rise of AMR represents one of the leading public health threats of the 21st century [3]. This highlights an urgent demand for novel antimicrobial strategies replacing or potentiating the conventional antibiotic-based therapies. 

Gram-negative *Pseudomonas aeruginosa* is an opportunistic pathogen affecting immunocompromised patients, and the leading cause of morbidity and mortality in cystic fibrosis [4]. This species is reported as one of the most difficult-to-treat resistant species, exhibiting resistance to piperacillin-tazobactam, ceftazidime, cefepime, aztreonam, meropenem, imipenem-cilastatin, ciprofloxacin, and levofloxacin [5]. In addition to developing genetic resistance, *P. aeruginosa* can also increase antibiotic tolerance by colonizing a variety of medical materials and forming resilient biofilms in the cystic fibrosis lung environment [6]. Biofilms represent multicellular populations residing within a self-produced matrix that protects cells against life-threatening attacks, including antibiotics. In bacteria, the biofilm formation and expression of many virulence factors are controlled by quorum sensing (QS) signaling, a central cell-to-cell communication process enabling cells to collectively modify their behavior in cell-density-dependent manner. Activation of the QS signaling system depends on the production, release, and group-wide detection of secreted/extracellular signaling molecules, autoinducers (AIs). AIs accumulate in the environment and, after reaching a threshold concentration, QS-mediated biofilm growth and virulence factor production are activated [7]. Blocking the central QS signaling system is considered one of the most promising strategies to combat pathogens effectively and sustainably, including *P. aeruginosa*, because an ideal anti-QS agent is likely to exert less selective pressure on the pathogen compared to conventional antibiotics [8].

*P. aeruginosa* PAO1, a spontaneous chloramphenicol-resistant mutant of the original PAO wound isolate, is a widely used reference strain in number of studies, as this strain uses four interconnected QS signaling (Las-, RhI-, PQS-, and IQS-QS) pathways to coordinate biofilm formation and virulence-related traits (e.g., elastase, rhamnolipids, and pyocyanin) [9,10,11,12]. Agents preventing the biofilm formation of *P. aeruginosa* or disrupting the formed biofilms could help treat chronic and recurring infections caused by this species. In this regard, several classes of quinoline derivatives have previously been investigated as potential anti-QS compounds to prevent biofilm formation due to their structural similarity to alkylquinolone AIs (e.g., 2-heptyl-3-hydroxy-4(1*H*)-quinolone, PQS, and its precursor 2-heptyl-4(1*H*)-quinolone, HHQ) produced by *P. aeruginosa*. Among these studies, the 4-amino-7-chloroquinoline scaffold has emerged as a promising candidate for developing anti-QS/biofilm agents that target multidrug-resistant bacteria [13,14,15,16,17]. Additionally, anthranilic acid is known to serve as a precursor of PQS biosynthesis [18], and halogenated anthranilic acids and their derivatives have been reported as inhibitors of PQS biosynthesis [19,20,21,22,23]. 

**Figure 1:**
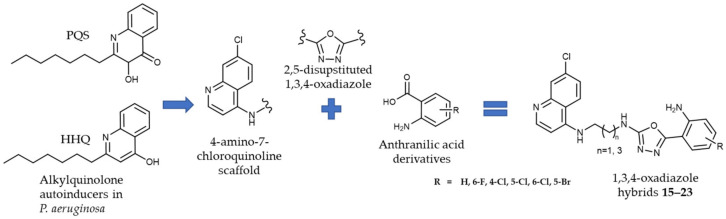
Novel 1,3,4-oxadiazoles as quorum sensing inhibitors.

The present study is a continuation of our work on anti-QS agents against Gram-negative biofilm-forming pathogens [24]. Here, we report a set of new series of hybrid molecules incorporating the quinoline and anthranilic acid scaffolds (Figure 1). For compound design, we applied molecular hybridization, i.e., covalent linking of two or more bioactive scaffolds to form a single molecule with improved properties. The goal was to combine complementary features from different molecules to create a new compound with improved potency or a broader spectrum of activity. The advantages of such compounds include increased efficacy, improved pharmacokinetics, and reduced risk of resistance development, side effects, and drug–drug interactions [25]. Thus, this method can significantly contribute to the development of new and effective strategies to combat bacterial infections and overcome issues related to antibiotic resistance. We have successfully implemented this approach to obtain a harmine-based antimalarial compound library [26,27,28,29,30,31]. Also, the concept of hybridization has been previously employed by various research groups within the subject area [32,33,34]. In this study, the generated hybrid compounds were first pre-screened for their anti-QS/biofilm and bactericidal activities using a Gram-negative *Chromobacterium violaceum*-based microscale screening platform, which allows compounds with genuine anti-QS activity to be distinguished from those with bactericidal activity in one single experiment [24,35,36,37,38,39]. *C. violaceum* is also an opportunistic pathogen capable of causing severe and potentially fatal manifestations in both immunocompromised and non-compromised hosts [40], and, therefore, serves as a suitable reporter for screening therapeutic agents. Here, we tested the most potent anti-QS compounds for their antibiofilm and antivirulence effects using *P. aeruginosa* PAO1 as the biofilm model with four interconnected QS systems. Our findings indicate that interrupting the PQS biosynthesis in *P. aeruginosa* could, indeed, lead to the discovery of new anti-infective agents against this species. We also show that testing bacterial models with less and more complex QS systems is necessary to identify/design anti-QS compounds with maximal efficacy against Gram-negative bacteria using multiple and overlapping QS systems. 

## 2. Results and Discussion

### 2.1. Chemistry

**Scheme 1:**
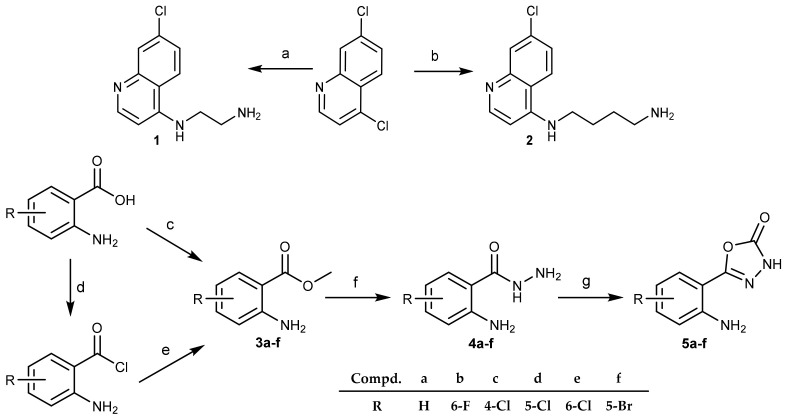
Synthesis of 4-amino-7-chloroquinoline and anthranilic acid intermediates **1**, **2,** and **5**. Reagents and conditions: (a) ethylenediamine, 120 °C; (b) 1,4-butylenediamine, 95 °C, MW; (c) SOCl_2_, MeOH, 60 °C; (d) SOCl_2_, dry toluene, 120 °C; (e) MeOH, 0 °C; (f) N_2_H_4_/H_2_O, 105 °C; (g) CDI, DMF, rt.

The compounds were obtained by standard synthetic procedures. The synthesis was divided into two parts. First, 4-amino-7-chloroquinoline- (**1** and **2**) and anthranilic acid-based (**5a**–**f**) building blocks were obtained, as outlined in Scheme 1. The 4-amino-7-chloroquinoline building blocks bearing a terminal amino group were prepared in the reaction of 4,7-dichloroquinoline and ethylenediamine (**1**) or 1,4-diaminobuthane (**2**) [41,42]. The anthranilic acid building blocks in the form of the corresponding 1,3,4-oxadiazol-2-ones (series **5**) were obtained in multiple reaction steps. First, anthranilic acid esters (series **3**) were either purchased (**3a**–**c**) or obtained from the corresponding anthranilic acids (**3d**–**f**). Hydrazides **4** were obtained from anthranilic acid esters **3** and hydrazine hydrate. 3-*H*-1,3,4-oxadiazole-2-ones (series **5**) were obtained from the corresponding hydrazides **4** in the presence of 1,1-carbonyldiimidazole (CDI) following the previously published procedure [43,44]. 

**Scheme 2:**
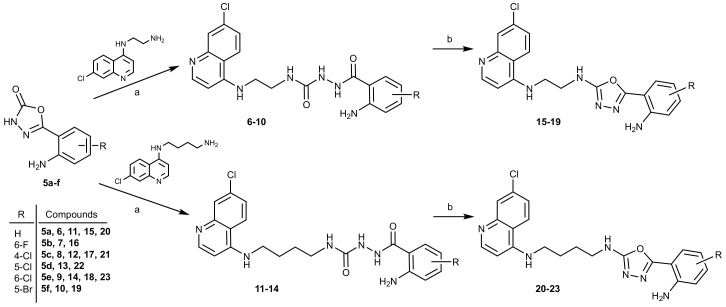
Synthesis of 2,5-disubstituted 1,3,4-oxadiazoles **15**–**19** and **20**–**23**. Reagents and conditions: (a) EtOH, 100 °C; (b) PPh_3_, CCl_4_, TEA, DCM, 46 °C.

The key reaction step was a nucleophilic attack of the primary amino group (**1** or **2**) on the carbonyl of the 3-*H*-1,3,4-oxadiazole-2-ones **5**, leading to the ring opening and merging of the two building blocks in the form of the corresponding acylsemicarbazides **6**–**14**. The reaction was conducted in ethanol at 100 °C in a sealed vial. In the case of acylsemicarbazides **7**, **10**, and **13**, we proceeded to the next reaction step without the purification of the product due to the low yields. The final reaction step presents intramolecular cyclization of acylsemicarbazides **6**–**14**. Under the dehydration conditions (utilizing triphenylphosphine, carbon tetrachloride, and triethylamine), the desired 1,3,4-oxadiazoles **15**–**23** were obtained (Scheme 2). 

The reagents used in the cyclization reaction include triphenylphosphine, carbon tetrachloride, and triethylamine. Dichloromethane was used as a solvent, which proved to be suitable due to its dielectric constant that affects the reaction rate [45]. Triethylamine was also added to the reaction mixture, as it promotes intramolecular cyclization by deprotonating the hydroxyl group of the enol form of acylsemicarbazide [46,47]. The final reaction step progressed well, and the reaction yields were slightly higher for the butan-1,4-diamine 1,3,4-oxadiazoles (**20**–**23**), ranging from 41 to 73%, as opposed to the ethan-1,2-diamine 1,3,4-oxadiazoles (**15**–**19**) (yields from 9 to 31%). The lowest reactivity was observed in the case of the 5-Br derivative.

Thus, we synthesized novel hybrid compounds **15**–**23**, derived from anthranilic acids and 4-amino-7-chloroquinoline-based amines, linked via a 1,3,4-oxadiazole ring, along with their precursors (4-amino-7-chloroquinoline **1** and **2**, and anthranilic acid-based building blocks **5a**–**f**). The 1,3,4-oxadiazole is a five-membered heterocyclic ring, composed of one oxygen, two carbons, and two nitrogen atoms, which was selected as a linker due to its variety of favorable physicochemical and biological properties. Notably, 1,3,4-oxadiazole is often used as a bioisostere of carbonyl-containing functional groups, such as amides, esters, and carbamates. Additionally, compounds containing 1,3,4-oxadiazole have demonstrated increased aqueous solubility, decreased hERG (the human Ether-a-go-go-Related Gene) inhibition, and improved metabolic stability when compared to those containing 1,2,4-oxadiazole [48,49,50]. The structural diversity of the title compounds was achieved by varying the length of the alkyl chain attached to the 4-amino-7-chloroquinoline scaffold (two or four carbon atoms) and the position and type of the halogen atom attached to the phenyl ring of the anthranilic acid scaffold (F, Cl, or Br). The 1H and 13C NMR spectroscopic data for the compounds are shown in the Supplementary Materials.

### 2.2. Anti-QS and Bactericidal Activity against the QS-Reporter Strain

**Figure 2:**
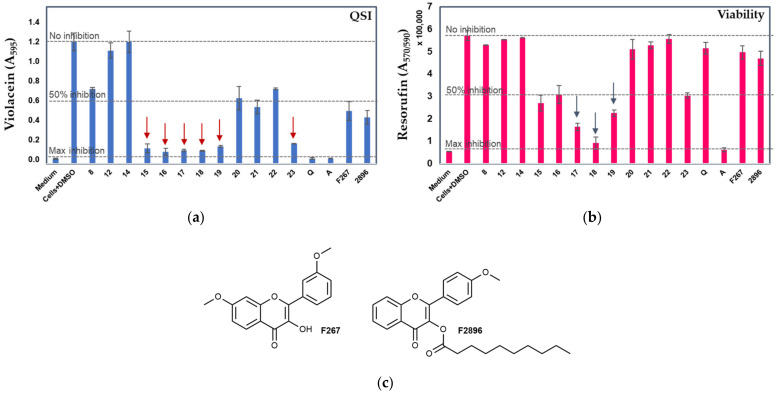
Comparison of the violacein production (**a**) and cell viability (**b**) of *C. violaceum* ATCC 31523 cultures with and without the tested compounds. Medium—culture medium with 2% DMSO; Cells+DMSO—*C. violaceum* cells with 2% DMSO; Q—quercetin; AZ—azithromycin (both at 400 μM). Error bars ± SD (n = 3–4). Z’ was > 0.5 in each screening experiment. (**c**) F267 and F2896—previously identified flavonols with demonstrated anti-QS activity [37,38]. Red arrows in (**a**) indicate the most active anti-QS compounds and blue arrows in (**b**) indicate the compounds with the most pronounced bactericidal activity (unpaired *t*-test with Welch’s correction, *p* < 0.05) in comparison to control cells with DMSO.

The developed compounds were first pre-screened for their anti-QS and bactericidal activities using the *C. violaceum* ATCC 31532 strain as the reporter and the previously established optimized conditions for 96-well format [39]. In this reporter strain, the activation of QS induces the expression of genes contributing to biofilm formation and the simultaneous synthesis of a deep-purple violacein [35] that can be quantitatively monitored [37,38]. When combined with a parallel resazurin staining, this high-throughput screening system allows genuine anti-QS compounds to be distinguished from those with bactericidal effect in one single experiment [37,38,51]. Due to the low reaction yields for the acylsemicarbazides series **6**–**14**, only three compounds of this group (**8**, **12** and **14**) were tested against the *C. violaceum* reporter (Table 1). Six out of twelve tested compounds at 400 µM (ethan-1,2,diamine 1,3,4-oxadiazoles **15**–**19** and butan-1,4-diamine 1,3,4-oxadiazole derivative **23**) inhibited the violacein production in *C. violaceum* almost to the same extent as quercetin (indicated as red arrows in Figure 2a, 83.5–90%). Among these compounds, two compounds (**17** and **18**) also exerted a strong bactericidal effect on the reporter strain (Figure 2b, more than 70%). At the same time, acylsemicarbazides **8**, **12** and **14** showed only weak or no effect on the QS-inducible violacein production or the viability of the reporter strain. It is worth mentioning that in the acylsemicarbazide series, similarly to the 1,3,4-oxadiazoles, the ethan-1,2,diamine compound **8** was more potent compared to the butan-1,4-diamine compounds **12** and **14**. 

**Figure 3:**
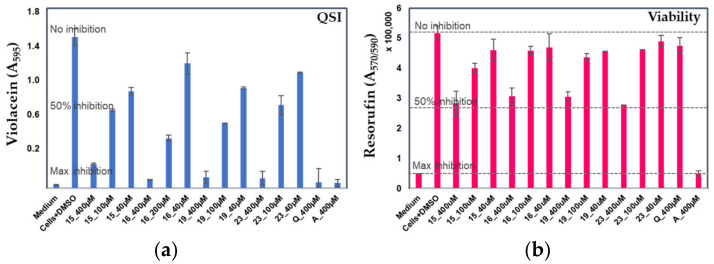
Dose–response analyses with the most promising anti-QS compounds (**15**, **16**, **19,** and **23**) at 400, 200, 100, and 40 µM concentrations of violacein production (**a**) and cell viability (**b**) of *C. violaceum* ATCC 31523 cultures with and without the tested compounds. Medium—culture medium with 2% DMSO; Cells+DMSO—*C. violaceum* cells with 2% DMSO; Q—quercetin; A—azithromycin (both at 400 μM). Error bars ± SD (n = 3–6). IC_50_ calculations; paired *t* test with two-tailed values of *p* < 0.05 were considered as significant.

Next, the most promising compounds (**15**, **16**, **19** and **23**) were selected for dose–response analyses. Figure 3 indicates that each compound with concentrations up to 100 μM reduced the violacein production by ca. 50% compared to the control cells with DMSO, while the viability of the reporter under the same conditions was only marginally affected. For compound **19**, IC_50_ of 63.15 μM (*p* < 0.05, confidence interval 47.6–83.8 μM) was measured. Compound **19** demonstrated the most efficient anti-QS activity; the violacein production in *C. violaceum* was reduced by more than 85% at 400 μM concentration, with a moderate bactericidal effect under the same conditions (47%). At a concentration of 100 μM, this compound was able to reduce the QS activity by more than 50% (Figure 3a), while no effect on cell viability was observed (Figure 3b). Taken together, our findings indicated that compounds **15**, **16**, **19,** and **23** demonstrated high anti-QS activity with only a minor bactericidal effect against *C. violaceum* already at concentrations of 100 µM.

### 2.3. Effect of Selected Compounds on Biofilm and Pyocyanin Production in P. aeruginosa PAO1

**Figure 4:**
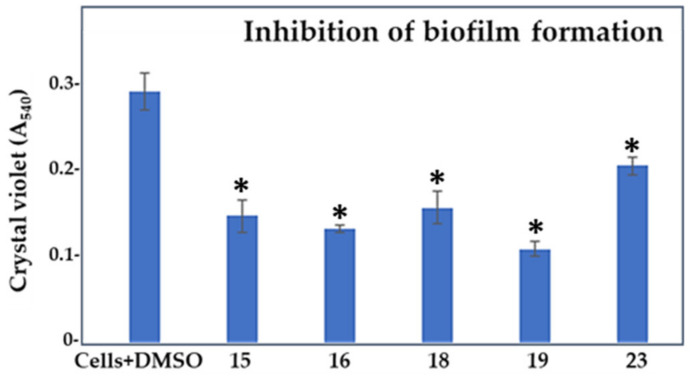
Effect of compounds **15**, **16**, **18**, **19,** and **23** at 100 μM concentrations on the biofilm formation of *P. aeruginosa* PAO1 compared to control cells treated with 1% DMSO. *, compounds showing significant reduction in the biofilm formation in comparison to the control cells with DMSO (1-ANOVA, *p* < 0.05).

Since the PQS-dependent QS system regulates both the biofilm formation and the production of pyocyanin in *P. aeruginosa* [11,12], we wished to assess if the compounds (**15**, **16**, **19**, and **23**) with genuine anti-QS activity against *C. violaceum* could interfere with these cellular processes using *P. aeruginosa* PAO1 as the model. Compound **18**, which showed the greatest bactericidal effect on *C. violaceum*, was also included in these analyses. The anti-QS activity of the compounds were tested at 100 μM because at that concentration compounds **15**, **16**, **19**, and **23** showed the most optimal anti-QS activity vs. bactericidal effect on the *C. violaceum* reporter. First, we tested the ability of the compounds to inhibit the biofilm formation (BFI) of *P. aerugionosa* PAO1. Figure 4 and Table 2 show that all compounds reduced the biofilm formation, with compound **19** demonstrating the highest antibiofilm effect (>60%, *p* < 0.05) compared to the control cells with 1% DMSO. However, compound **19** also conferred the strongest inhibitory effect on the cell growth (Table 2—GI), suggesting that this compound could predominantly act as a bactericidal agent against the *P. aeruginosa* strain. In view of this, we next calculated the biofilm index, including both the biofilm mass and the cell count, for each compound (Table 2). As a result, among the tested compounds, compound **15** showed the greatest potency against *P. aeruginosa* by reducing its ability to form biofilm by nearly 50% with a minimal effect on cell growth (*p* < 0.05), implying the anti-QS nature of this compound. 

**Figure 5:**
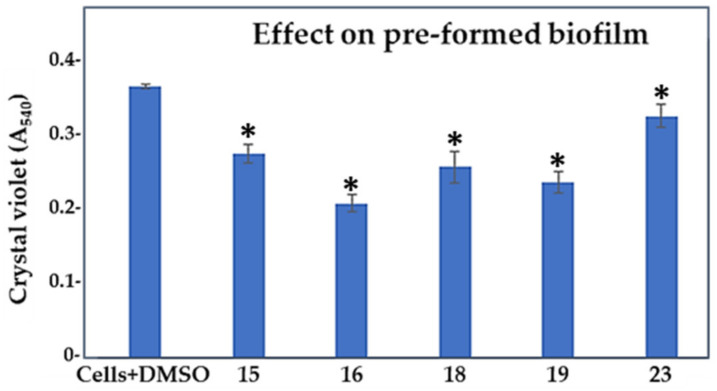
Effect of compounds **15**, **16**, **18**, **19,** and **23** in 100 μM concentrations on the established biofilm of *P. aeruginosa* PAO1 compared to control cells treated with 1% DMSO. *, compounds showing statistically significant change in comparison to the control cells with DMSO (1-ANOVA, *p* < 0.05).

In the next experiment, we allowed *P. aeruginosa* PAO1 to form biofilm for 24 h and then treated the pre-formed biofilm with the selected five compounds (Figure 5, Table 2—column BE). Compounds **16** and **19** were the most effective biofilm eradicators, reducing the biofilm mass by 43% and 35%, respectively (*p* < 0.05). Compound **23**, by reducing the biofilm mass by 11%, was considered the least-effective biofilm eradicator. Compound **15,** in addition to its predicted anti-QS activity (ca., 50%, BHI), could also act on mature biofilms, as nearly 25% less biofilm mass was detected after treating the pre-formed biofilms with this compound. In addition, a comparison of all cell growth inhibitory values (GI) with those indicating the biofilm eradication efficiency for each tested compound strengthens the idea that compound **15** is a genuine anti-QS agent, showing activity also on formed biofilms. 

All compounds showed significantly higher inhibitory effects on cell growth (by 30% to 45%, Table 2—GI), with compounds **16** and **19** showing the greatest effect. As these compounds were also more efficient biofilm formation inhibitors, we suggest that they predominantly acted by killing the PAO1 cells (possibly including both planktonic and biofilm cells) rather than interfering with the PQS-QS system. To complement these analyses, we also measured the minimum inhibitory concentration (MIC) and minimum bactericidal concentration (MBC) for the five indicated compounds. The MIC was 1600 μM, and the MBC was 3200 μM for each compound. Since the compounds were initially dissolved in DMSO, we also monitored the effect of DMSO alone at corresponding concentrations (ranging from 1% to 32% *v*/*v*) and obtained the following inhibitory effects: 4.82 ± 0.95% for 1% DMSO, 12.53 ± 1.12% for 2% DMSO, 18.72 ± 1.76% for 4% DMSO, 28.12 ± 1.97% for 8% DMSO, 78.13 ± 2.23% for 16% DMSO, and 97.21 ± 2.11% for 32% DMSO. Thus, taking the DMSO-mediated effects into account, the actual MIC and MBC values for the compounds are estimated to be significantly higher. These findings also indicate that a concentration of 100 µM for testing the selected compounds was well below their MIC and MBC values, ensuring that the detected antibiofilm effect, rather than antibacterial or bactericidal activity, likely resulted from interference with the PQS-QS signaling system.

**Figure 6:**
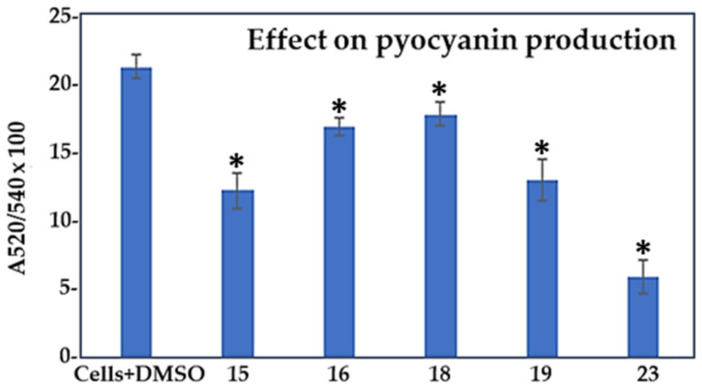
Effect of compounds **15**, **16**, **18**, **19,** and **23** at 100 μM concentrations on the production of pyocyanin by *P. aeruginosa* PAO1 compared to control cells treated with 1% DMSO. Results are expressed as a ratio (A520/A570) × 100 to normalize pyocyanin absorbance to the cell count. *, compounds showing statistically significant *change* in comparison to the control cells with DMSO (1-ANOVA, *p* < 0.05).

We also examined if the selected five compounds (**15**, **16**, **18**, **19**, and **23**) had any impact on the production of pyocyanin, as this extracellular virulence factor is regulated by the PQS-QS system in *P. aeruginosa* [11]. The effect of compounds on the synthesis of this pigment was tested at the 100 µM level, since statistically significant differences in biofilm formation and pre-formed biofilm assays were obtained at this concentration. Figure 6 and Table 2 (column PI) show that compound **23** was the most effective antipyocyanin agent, reducing the production of this virulence factor by more than 70% in comparison to the control cells (*p* < 0.05). Compounds **15** and **19** were the second best by decreasing the pyocyanin level by ca. 40% (*p* < 0.05). Compounds **16** and **18**, with predicted bactericidal effects, were the least effective, which is likely the consequence of the reduced cell growth detected for the two compounds. Since compound **23** displayed only a minor antibiofilm effect, we conclude that this could be a genuine antivirulence compound against *P. aeruginosa* PAO1.

### 2.4. Factors Limiting the Efficacy of Compounds Targeting PQS-QS of P. aeruginosa 

**Figure 7:**
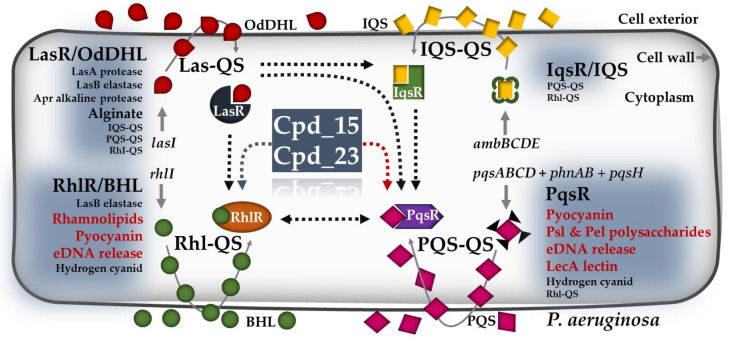
Schematic representation of the four QS signaling networks present in *P. aeruginosa*, with indicated QS-mediated pathways likely to be affected by compounds 15 and 23. Black dotted arrows indicate a stimulatory effect between the four QS interactive systems. Dotted arrows in grey and red indicate indirect and direct effects of the indicated compounds on Rhl-QS- and PQS-QS-mediated activities, respectively. AIs refer to *N*-butyrylhomoserine lactone (BHL) and *N*-(3-oxododecanoyl)-homoserine lactone (OdDHL). The abbreviations 2-heptyl-3-hydroxy-4-quinolone and 2-(2-hydroxy phenyl)-thiazole-4-carbaldehyde are denoted as PQS. Additionally, IQS stands for Integrated Quorum Sensing Signal. LasR, RhlR, IqsR, and PqsR are the regulators of the indicated QS systems. Furthermore, *ambBCDE* represents the non-ribosomal peptide synthase gene cluster involved in IQS synthesis, while *pqsABCD*, *phnAB*, and *pqsH* are the genes contributing to PQS synthesis. The genes *lasI* and *RhlI* code for OdDHL and BHL, respectively. In the context of this figure, Cpd refers to the compounds, and red letters in bold indicate the metabolic activities targeted by Cpd_15 or Cpd_23. This figure was created according to related studies on *P. aeruginosa* [11,57,58,59].

Since none of the tested compounds were able to completely block the biofilm formation, eradicate the pre-formed biofilms, or prevent pyocyanin production, we suggest that the presence of the four interconnected QS systems in *P. aeruginosa* PAO1 (Figure 7) explains the obtained results. The Las-QS system acts as the top of the QS hierarchy, governing both the Rhl- and PQS-based QS systems and inducing the synthesis of alginate, a negatively charged polysaccharide important for biofilm formation [11]. In addition to alginate, *P. aeruginosa* PAO1 produces several other polysaccharides, along with lipopolysaccharides, including rhamnolipids, lectin, Psl, and Pel, each playing important roles in biofilm formation, development, stabilizing the biofilm matrix, and protecting the mature biofilm against invading substances [52]. Among these, lectin, Psl, and Pel are controlled by the PQS-QS system, while the synthesis of rhamnolipids is activated through Las- and Rhl-dependent QS [11]. Considering the close interaction between the PQS- and Rhl-QS systems in *P. aeruginosa*, it is possible that the mechanism of action for compound **15** included interference with the synthesis of lectin, Psl, Pel, and/or rhamnolipids, rather than directly killing the cells within the biofilm (Figure 7). On the other hand, the eradication of pre-formed biofilms by this compound was not caused by the reduced cell growth, which was observed with the other compounds. Thus, we cannot exclude the possibility that compound **15** could have also prevented the generation of one or more of the charged polysaccharides [53,54,55], or other cell surface-associated/secreted factors (e.g., pyocyanin, proteins, extracellular DNA/eDNA, etc.) [56,57] contributing to the formation of a stable biofilm matrix. Nevertheless, since *P. eruginosa* can also use Las- and Rhl-QS to stimulate biofilm formation, and since compound **15** could inhibit the biofilm formation by only 50%, we suggest that either the Las- or Rhl-QS systems may have also contributed to the biofilm formation. 

Our findings indicate that compound **23**, instead of exerting significant antibiofilm effects, preferably targets the PQS-QS-mediated pyocyanin production. This virulence factor has been shown to be secreted by 95% of *P. aeruginosa* isolates, including the strain PAO1 [60]. It not only confers increased virulence to the biofilm cells but also plays a crucial role in releasing eDNA, which serves as an essential component of the biofilm matrix, providing structural support and aiding in biofilm formation [57]. In *P. aeruginosa*, the synthesis of pyocyanin is regulated by both the PQS- and Rhl-QS systems (Figure 7), likely explaining why compound **23** does not fully inhibit pyocyanin synthesis. As we observed only a marginal antibiofilm effect on PAO1 with this compound, we suggest that the increased synthesis of other matrix-associated factors via PQS-QS-independent pathways may compensate for the pyocyanin-stimulated biofilm formation. 

Notably, PQS has also been shown to exert QS-independent functions, such as iron acquisition, cytotoxicity, outer membrane vesicle biogenesis, or host immune modulation, indicating a pleiotropic role of this AI in *P. aeruginosa* [61,62]. Studies have also demonstrated that QS *lasR* mutants easily develop during cystic fibrosis in vivo [62,63,64,65], highlighting the importance of PQS-QS in compensating for a lack of the Las-QS system; LasR activates also the RhlR-activated genes, including genes contributing to virulence and biofilm formation (e.g., pyocyanin and rhamnolipids) (Figure 7). Thus, PQS could overcome the dependency on LasR by activating the expression of the Rhl-QS system and the production of downstream virulence factors [59] (Figure 7). In view of this, strategies targeting both the PQS-dependent and -independent pathways could, indeed, be the method of choice to treat *P. aeruginosa* infections in clinical settings. 

Taken together, the 1,3,4-oxadiazoles **15** and **23** reported in the present study showed the highest promise for designing new and more effective anti-QS strategies against *P. aeruginosa*. To meet that goal, our study also stresses the importance of the interactive QS systems, which should be considered when aiming to maximize efforts against this clinically relevant pathogen.

## 3. Materials and Methods

### 3.1. Chemistry

#### 3.1.1. General Information

Melting points of compounds **9** and **15**–**19** were determined by differential scanning calorimetry (DSC). Thermograms were recorded using DSC 822e (Mettler Toledo), and analyzed by STARe software (V16.20b) (Mettler-Toledo). Melting points of compounds **8**, **11**, **12**, **14,** and **20**-**23** were determined on a Stuart Melting Point Apparatus (Barloworld Scientific, UK) in open capillaries and were uncorrected. FTIR-ATR spectra were recorded using a Fourier-Transform Infrared Attenuated Total Reflection UATR Two spectrometer (PerkinElmer, Waltham, MA, USA) in the range from 450 to 4000 cm^–1^. ^1^H and ^13^C NMR spectra were recorded on a Bruker Avance III 600, Bruker Avance DRX500, Bruker Avance AV400, and Bruker Avance DPX300 spectrometers operating at 300 or 400 MHz for the ^1^H and 75, 101, or 151 MHz for the ^13^C nuclei, BrukerDPX 300 MHz, Bruker AV400 MHz, and Bruker DRX 500 MHz (Bruker, Billerica, MA, USA). ^1^H and ^13^C NMR spectra were recorded on Bruker Avance III 600, Bruker Avance DRX500, Bruker Avance AV400, and Bruker Avance DPX300 spectrometers (Billerica, MA, USA), Chemical shifts are reported in parts per million (ppm) using tetramethylsilane (TMS) as a reference in the ^1^H and DMSO residual peak as a reference in the ^13^C spectra (39.52 ppm). Data for ^1^H NMR are described as follows: chemical shift (δ in ppm), multiplicity (s: singlet; d: doublet; t: triplet; q: quartet; m: multiplet; bs: broad signal), integration, and coupling constant J (Hz). Samples were measured in DMSO-d_6_ solutions at 20 °C in 5 mm NMR tubes. Mass spectra were recorded on Agilent 1200 Series HPLC coupled with Agilent 6410 Triple Quad (Agilent Technologies, St. Clara, CA, USA). LCMS data were acquired on a Waters Acquity UPLC instrument using a Waters Acquity UPLC C18 (2.1 × 50 mm, 1.7 um) column, water/MeCN gradient, and 0.1% (*v*/*v*) formic acid as an acidic modifier or 0.05% (*v*/*v*) NH_4_OH as a basic modifier. The column eluent was analyzed using a Waters SQ mass spectrometer with ESI scanning in both positive- and negative-ion modes from 100 to 2000 Da and reaction conversions expressed as percentage (where applied) by using a UV detector at 254 nm. 

All compounds were routinely checked by analytical thin-layer chromatography (TLC) with silica gel 60F-254 glass plates (Merck, Darmstadt, Germany) using DCM/MeOH 7.5:2.5, 7:3, 8.5:1.5, 8:2, 9.5:0.5, and 9:1 and cyclohexane/EtOAc/MeOH 3:1:0.5 and 1:1:0.5 as the solvent system. Spots were visualized by UV light (λ = 254 nm; 365 nm) and iodine vapor. Column chromatography was performed on silica gel 0.063–0.200 mm (Sigma-Aldrich, St Louis, CA, USA) with the same eluents used for TLC. Flash chromatography was performed on Interchem Puriflash XS 520 Plus, Interchim Puriflash SiHC (12–25 g; 15 μm) columns. All chemicals and solvents were of analytical grade and purchased from commercial sources. Methyl anthranilates were purchased from commercial sources (methyl 2-aminobenzoate from Sigma Aldrich, USA, methyl 2-amino-6-fluorobenzoate and methyl 2-amino-4-chlorobenzoate from Fluorochem, UK).

Dry toluene was obtained using the following procedure: toluene was extracted with water and dried over anhydrated calcium chloride, distilled and stored over elemental sodium. Dry DCM: DCM was extracted with water and dried over anhydrated calcium chloride and distilled. Dry DMF was stored over activated molecular sieves.

#### 3.1.2. Synthesis of 4-Amino-7-chloroquinoline Intermediates **1** and **2**

##### *N*^1^-(7-Chloroquinolin-4-yl)butane-1,4-diamine (**1**)

Compound **1** was prepared according to the published procedure [42]. A mixture of 4,7-dichloroquinoline (5.00 g, 25.3 mmol) and 1,2-diaminoethane (13.50 g, 227.2 mmol) was gradually heated to 80 °C for 1 h while stirring. The reaction mixture was then heated to 120 °C and stirred for an additional 6 h and poured on iced water and stirred for 18 h. An amount of 5.2 g (93%) of white solid **1** was filtered off, washed with water, and vacuum-dried.

^1^H NMR (DMSO-*d*_6_, δ ppm, *J*/Hz) δ 8.38 (d, *J* = 4.83 Hz, 1H), 8.28 (d, *J* = 9.05 Hz, 1H), 7.78 (d, *J* = 3.02 Hz, 1H), 7.43 (d, *J* = 8.99 Hz, 1H), 7.29–7.20 (bs, 1H), 6.48 (d, *J* = 5.99 Hz, 1H), 3.25 (q, *J* = 5.99 Hz, 2H), 2.82 (t, *J* = 6.74 Hz, 2H).

##### *N*^1^-(7-Chloroquinolin-4-yl)butane-1,4-diamine (**2**)

Compound **2** was prepared according to the published procedure [41]. A mixture of 4,7-dichloroquinoline (0.400 g, 2.02 mmol) and 1,4-diaminobutane (1.78 g, 20,2 mmol) was stirred under microwave irradiation (300 W) at 95 °C for 1 h. The reaction mixture was then diluted with dichloro-methane (100 mL), extracted with 5% NaOH (3 × 100 mL), and washed with water (1 × 100 mL). The organic layer was dried over anhydrous sodium sulfate, filtered, and evaporated under reduced pressure. The crude product was triturated with diethyl ether to give 0.428 g (86%) of white solid **2**.

^1^H NMR (DMSO-*d*_6_, δ ppm, *J*/Hz) δ 8.39 (d, *J* = 5.4 Hz, 1H), 8.27 (d, *J* = 9.0 Hz, 1H), 7.78 (d, *J* = 2.2 Hz, 1H), 7.43 (dd, *J* = 9.0, 2.2 Hz, 1H), 7.39 (s, 1H), 6.46 (d, *J* = 5.5 Hz, 1H), 3.26 (dd, *J* = 12.0, 6.9 Hz, 2H), 2.59 (t, *J* = 6.9 Hz, 2H), 1.69 (dt, *J* = 14.8, 7.4 Hz, 2H), 1.46 (dt, *J* = 14.0, 6.9 Hz, 2H).

#### 3.1.3. Synthesis of Methyl Anthranilates **3** and Hydrazides **4**

Methyl anthranilates **3a–f** were either purchased from commercial sources (**3a**, methyl 2-aminobenzoate; **3b**, methyl 2-amino-6-fluorobenzoate; and **3c**, methyl 2-amino-4-chlorobenzoate) or prepared from the corresponding anthranilic acid (**3d**, methyl 2-amino-5-chlorobenzoate; **3e**, methyl 2-amino-6-chlorobenzoate; and **3f**, methyl 2-amino-5-bromobenzoate) according to the previously published procedures [43,44].

Hydrazides **4a–f** were obtained in the reaction of the corresponding methyl anthranilate (**3a–f**) and hydrazine hydrate according to the previously published procedure [43].

#### 3.1.4. General Procedure for the Synthesis of 5-(2-Aminophenyl)-l,3,4-oxadiazole-2(3*H*)-ones **5a–f**

The compounds **5a–f** were obtained according to slightly modified procedures [43]. 

1,1′-carbonyldiimidazole (0.254 g, 1.6 mmol) was added to a solution of the corresponding anthranilic acid hydrazide **4a–f** (1.4 mmol) in dry DMF (1 mL). The reaction mixture was stirred for 4-18 h at room temperature, followed by the addition of ethyl acetate (30 mL) and extraction with brine (3 × 30 mL). The organic layer was dried over anhydrous sodium sulfate, filtered, and evaporated under reduced pressure. The crude product was used in successive reaction steps with **(5d**) or without **(5a–c**, **5e**, **5f**) trituration with diethyl ether.

##### 5-(2-Aminophenyl)-1,3,4-oxadiazol-2(3*H*)-one (**5a**)

Hydrazide **4a**: 0.212 g; ^1^H NMR (DMSO-d_6_): *δ* 12.70–12.27 (bs, 1H), 7.44 (dd, 1H, *J* = 8.0, 1.6 Hz), 7.21 (dt, 1H, *J* = 7.2, 1.6 Hz), 6.84 (dd, 1H, *J* = 8.4, 0.8 Hz), 6.64 (t, 1H, *J* = 7.2, 1.2 Hz),6.37–6.29 (s, 2H).

##### 5-(2-Amino-6-fluorophenyl)-1,3,4-oxadiazol-2(3*H*)-one (**5b**)

Hydrazide **4b**: 0.236 g.

##### 5-(2-Amino-4-chlorophenyl)-1,3,4-oxadiazol-2(3*H*)-one (**5c**)

Hydrazide **4c**: 0.260 g.

##### 5-(2-Amino-5-chlorophenyl)-1,3,4-oxadiazol-2(3*H*)-one (**5d**)

Hydrazide **4d**: 0.260 g; yield: 0.177 g (60%); IR (ATR, *ν*/cm^−1^) 3465, 3364, 3170, 1734, 1628, 1562, 1488, 1310, 1257, 1161, 1042, 813, 717; ^1^H NMR (DMSO-d_6_): *δ* 12.63 (bs, 1H), 7.39 (d, 1H, *J* = 2.6 Hz), 7.25 (dd, 1H, *J* = 8.9, 2.6 Hz), 6.88 (d, 1H, *J* = 8.9 Hz), 6.45 (s, 2H).

##### 5-(2-Amino-6-chlorophenyl)-1,3,4-oxadiazol-2(3*H*)-one (**5e**)

Hydrazide **4e**: 0.260 g.

##### 5-(2-Amino-5-bromophenyl)-1,3,4-oxadiazol-2(3*H*)-one (**5f**)

Hydrazide **4f**: 0.322 g.

#### 3.1.5. General Procedure for the Synthesis of Acylsemicarbazides (**6**–**10**)

*N*^1^-(7-chloroquinolin-4-yl)athane-1,2-diamine **1** (0.213 g, 0.961 mmol) was added to a suspension of an appropriate 5-(2-aminophenyl)-l,3,4-oxadiazole-2(3*H*)-one **5** (0.915 mmol) in 5–10 mL of anhydrous EtOH. The reaction mixture was refluxed at 100 °C for 18–72 h and cooled to room temperature. The crude product was filtered off and washed with EtOH and vacuum-dried (**6**–**9**) or purified using flash chromatography (**10**) (eluent DCM/MeOH 80:20).

##### 2-(2-Aminobenzoyl)-*N*-(2-((7-chloroquinolin-4-yl)amino)ethyl)hydrazine-1-carboxamide (**6**)

Oxadiazol-2-one **5a**: 0.162 g; reaction time: 48 h; yield 0.179 g (49%); mp nd; ^1^H NMR (DMSO-d_6_): *δ* 9.93–9.72 (bs, 1H), 8.40 (d, 1H, *J* = 5.2 Hz), 8.16 (d, 1H, *J* = 8.8 Hz), 8.05–7.92 (bs, 1H), 7.79 (d, 1H, *J =* 2.8 Hz), 7.60 (d, 1H, *J* = 8.4 Hz), 7.43 (dd, 1H, *J* = 10.4, 2.4 Hz), 7.48–7.36 (m, 1H), 7.18 (dt, 1H, *J* = 8.4, 1.2 Hz), 6.86–6.75 (bs, 1H), 6.72 (d, 1H, *J* = 6.00 Hz), 6.57 (d, 1H, *J* = 5.6 Hz), 6.51 (t, 1H, *J* = 7.6 Hz), 6.44–6.32 (bs, 2H), 3.40–3.25 (m, 4H) ppm; ^13^C NMR (DMSO-d_6_): *δ*)*: δ* 168.9, 159.3, 152.0, 150.1, 150.0, 149.0, 133.4, 132.2, 128.4, 127.5, 124.1, 123.8, 117.3, 116.3, 114.4, 112.4, 98.6, 43.4, 37.8 ppm; ESI-MS: *m/z* 399.14 (M + 1)^+^.

##### 2-(2-Amino-6-fluorobenzoyl)-*N*-(2-((7-chloroquinolin-4-yl)amino)ethyl)hydrazine-1-carboxamide (**7**)

Oxadiazol-2-one **5b**: 0.179 g, reaction time: 18 h; the structure of the product was indirectly confirmed in the successive reaction step.

##### 2-(2-Amino-4-chlorobenzoyl)-*N*-(2-((7-chloroquinolin-4-yl)amino)ethyl)hydrazine-1-carboxamide (**8**)

Oxadiazol-2-one **5c**: 0.194 g; reaction time: 72 h; yield: 0.177 g (45%); mp 222.5–224 °C; IR (ATR, *ν*/cm^−1^) 3344, 1661, 1630, 1613, 1587, 1539, 1449, 1370, 1255, 1235, 1146, 1081, 941, 877, 807; ^1^H NMR (DMSO-d_6_): *δ* 10.00–9.87 (bs, 1H), 8.41 (d, 1H, *J* = 5.4 Hz), 8.16 (d, 1H, *J* = 8.4 Hz), 8.02 (s, 1H), 7.79 (d, 1H, *J* = 2.4 Hz), 7.61 (d, 1H, *J* = 9,0 Hz), 7.44 (dd, 1H, *J* = 9.6, 2.4 Hz), 7.41 (t, 1H, *J* = 4.8 Hz), 6.88–6.80 (bs, 1H), 6.79 (d, 1H, *J* = 2.4 Hz), 6.73–6.65 (bs, 2H), 6.56 (d, 1H, *J* = 5.4 Hz), 6.54 (dd, 1H, *J* = 9.0, 1.8 Hz), 3.38-3.27 (m, 4H) ppm; ^13^C NMR (DMSO-d_6_): *δ* 168.2, 159.2, 152.0, 151.2, 150.0, 149.0, 136.7, 133.3, 130.4, 127.5, 124.1, 123.8, 117.3, 115.0, 114.1, 111.2, 98.6, 43.3, 37.8 ppm; ESI-MS: *m/z* 432.97 (M + 1)^+^.

##### 2-(2-Amino-6-chlorobenzoyl)-*N*-(2-((7-chloroquinolin-4-yl)amino)ethyl)hydrazine-1-carboxamide (**9**)

Oxadiazol-2-one **5e**: 0.194 g; reaction time: 18 h; yield: 0.192 g (49%); mp 213 °C; IR (ATR, *ν*/cm^−1^) 3231, 1687, 1629, 1603, 1582, 1540, 1457, 1428, 1319, 1250, 1146, 907, 877, 811, 763; ^1^H NMR (DMSO-d_6_): *δ* 10.08–9.85 (bs, 1H), 8.56 (s, 1H), 8.41 (d, 1H, *J* = 6.0 Hz), 8.18 (d, 1H, *J* = 9.0 Hz), 7.79 (d, 1H, *J* = 2.0 Hz), 7.46 (dd, 1H, *J* = 4.5, 2.0 Hz), 7.40 (t, 1H, *J* = 5.0 Hz), 7.05 (t, 1H, *J* = 7.5 Hz), 6.63 (d, 1H, *J* = 9.0 Hz), 6.63 (s, 1H), 6.56 (d, 1H, *J* = 5.0 Hz), 6.54 (d, 1H, *J* = 6.5 Hz), 5.99 (s, 2H), 3.46-3.41 (m, 4H) ppm; ^13^C NMR (DMSO-d_6_): *δ* 165.9, 159.2, 152.0, 150.0, 149.0, 147.9, 133.4, 130.6, 130.5, 127.5, 124.2, 123.9, 119.1, 117.3, 115.1, 113.2, 98.7, 43.3, 38.1 ppm; ESI-MS: *m/z* 433.1 (M + 1)^+^.

##### 2-(2-Amino-5-bromobenzoyl)-*N*-(2-((7-chloroquinolin-4-yl)amino)ethyl)hydrazine-1-carboxamide (**10**)

Oxadiazol-2-one **5f**: 0.234 g; reaction time: 18 h; the structure of the product was indirectly confirmed in the successive reaction step.

#### 3.1.6. General Procedure for the Synthesis of Acylsemicarbazides (**11**–**14**)

*N*^1^-(7-chloroquinolin-4-yl)butane-1,4-diamine **2** (0.240 g, 0.961 mmol) was added to a suspension of an appropriate 5-(2-aminophenyl)-l,3,4-oxadiazole-2(3*H*)-one **5** (0.915 mmol) in 5 mL of anhydrous EtOH. The reaction mixture was refluxed at 100 °C for 24–48 h and cooled at room temperature, the organic solvent was evaporated and the crude product was purified using column chromatography (DCM/MeOH 75:25).

##### 2-(2-Aminobenzoyl)-*N*-(4-((7-chloroquinolin-4-yl)amino)butyl)hydrazine-1-carboxamide (**11**)

Oxadiazol-2-one **5a**: 0.162 g; yield: 0.155 g (40%); mp 184–185 °C; IR (ATR, *ν*/cm^−1^) 3328, 2934, 1645, 1613, 1580, 1532, 1450, 1368, 1251, 1137, 852, 806, 748; ^1^H NMR (DMSO-d_6_): *δ* 9.78 (s, 1H), 8.38 (d, 1H, *J* = 5,4 Hz), 8.28 (d, 1H, *J* = 9.0 Hz), 7.77 (d, 1H, *J* = 2.2 Hz), 7.69 (s, 1H), 7.58 (d, 1H, *J* = 7.9 Hz), 7.43 (dd, 1H, *J* = 9.0, 2.3 Hz), 7.34 (t, 1H, *J* = 5.4 Hz), 7.20–7.13 (m, 1H), 6.70 (dd, 1H, *J* = 8.4, 1.2 Hz), 6.52–6.46 (m, 3H), 6.41 (s, 2H), 3.28 (q, 2H, *J* = 7.1 Hz), 3.10 (q, 2H, *J* = 6.6 Hz), 1.66 (q, 2H, *J* = 7.3 Hz), 1.53 (q, 2H, *J* = 7.1 Hz) ppm; ^13^C NMR (DMSO-d_6_): *δ* 168.9, 158.7, 151.9, 150.1, 149.8, 149.0, 133.3, 132.1, 128.0, 127.4, 124.1, 124.0, 117.4, 116.2, 114.4, 112.7, 98.7, 42.2, 38.9, 27.6, 25.1 ppm; ESI-MS: *m/z* 427.1 (M + 1)^+^.

##### 2-(2-Amino-4-chlorobenzoyl)-*N*-(4-((7-chloroquinolin-4-yl)amino)butyl)hydrazine-1-carboxamide (**12**)

Oxadiazol-2-one **5c**: 0.194 g; yield 0.086 g (21%); mp 174–175 °C; IR (ATR, *ν*/cm^−1^) 3306, 2956, 2869, 1612, 1578, 1482, 1430, 1368, 1322, 1253, 1207, 1141, 1078, 920, 847, 809, 766, 643; ^1^H NMR (DMSO-d_6_): *δ* 9.86 (s, 1H), 8.39 (d, 1H, *J* = 5,4 Hz), 8.29 (d, 1H, *J* = 9.1 Hz), 7.78 (d, 1H, *J* = 2.2 Hz), 7.72 (s, 1H), 7.58 (d, 1H, *J* = 8.5 Hz), 7.44 (dd, 1H, *J* = 9.0, 2.3 Hz), 7.42 (t, 1H, *J* = 5.3 Hz), 6.77 (d, 1H, *J* = 2.2 Hz), 6.66 (s, 2H), 6.52 (dd, 2H, *J* = 8.5, 2.2 Hz), 6.49 (d, 1H, *J* = 5.5 Hz), 3.29 (q, 2H, *J* = 7.1 Hz), 3.09 (q, 2H, *J* = 6.6 Hz), 1.69–1.62 (m, 2H), 1.56–1.49 (m, 2H) ppm; ^13^C NMR (DMSO-d_6_): *δ* 168.1, 158.6, 151.5, 151.1, 150.3, 148.6, 136.6, 133.5, 130.3, 127.1, 124.2, 124.1, 117.4, 115.0, 114.1, 111.5, 98.7, 42.2, 38.9, 27.6, 25.1 ppm; ESI-MS: *m/z* 461.1 (M + 1)^+^.

##### 2-(2-Amino-5-chlorobenzoyl)-*N*-(4-((7-chloroquinolin-4-yl)amino)butyl)hydrazine-1-carboxamide (**13**)

Oxadiazol-2-one **5d**: 0.194 g; the structure of the compounds was indirectly confirmed in the next reaction step.

##### 2-(2-Amino-6-chlorobenzoyl)-*N*-(4-((7-chloroquinolin-4-yl)amino)butyl)hydrazine-1-carboxamide (**14**)

Oxadiazol-2-one **5e**: 0.194 g; yield 0.085 g (22%); mp 144–155.5 °C; IR (ATR, *ν*/cm^−1^) 3345, 3229, 2934, 1651, 1601, 1579, 1532, 1469, 1450, 1368, 1332, 1279, 1250, 1206, 1137, 1081, 1041, 903, 852, 805, 644; NMR (DMSO-d_6_): *δ* 9.92 (s, 1H), 8.39 (d, 1H, *J* = 5.4 Hz), 8.28 (d, 1H, *J* = 9.1 Hz), 8.17 (s, 1H), 7.77 (d, 1H, *J* = 2.2 Hz), 7.42 (dd, 1H, *J* = 9.0, 2.3 Hz), 7.32 (t, 1H, *J* = 5.4 Hz), 7.04 (t, 1H, *J* = 8.1 Hz), 6.60 (dd, 1H, *J* = 8.3, 1.0 Hz), 6.54 (dd, 2H, *J* = 7.8, 0.9 Hz), 6.49 (d, 1H, *J* = 5.5 Hz), 6.35 (t, 1H, *J* = 5.8 Hz), 5.93 (s, 2H), 3.29 (q, 2H, *J* = 7.1 Hz), 3.13 (q, 2H, *J* = 6.6 Hz), 1.72–1.64 (m, 2H), 1.59–1.51 (m, 2H) ppm; ^13^C NMR (DMSO-*d*_6_): *δ* 166.0, 158.6, 152.0, 150.1, 149.1, 147.9, 133.3, 130.6, 127.5, 124.1, 124.0, 119.1, 117.5, 115.1, 113.2, 98.7, 42.1, 39.1, 27.5, 25.1 ppm; ESI-MS: *m/z* 461.1 (M +1 )^+^.

#### 3.1.7. General Procedure for the Synthesis of Oxadiazoles (**15**–**19**)

Triphenylphosphine (0.168 g, 0.639 mmol for **15**,**17**–**19** or 0.223 g, 0.852 mmol for **16**), TEA (0.238 mL, 0.172 g, 1.704 mmol), and carbon tetrachloride (0.091 mL, 0.105 g, 0.682 mmol) were added to the solution of an appropriate acylsemicarbazide (**6**–**10**) (0.426 mmol) in dry dichloromethane (4 mL). The reaction mixture was refluxed for 18–72 h at 46 °C. The reaction mixture was cooled to r.t. and 25 mL of dichloromethane was added. The organic layer was extracted with water (3 × 20 mL), dried over anhydrous sodium sulfate, filtered, and evaporated under reduced pressure. The crude product was purified using flash chromatography (Interchim 12 g, 15 μm) and trituration with MeOH (**15**–**18**) or diisopropyl ether (**19**).

##### *N*′-(5-(2-Aminophenyl)-1,3,4-oxadiazol-2-yl)-*N*-(7-chloroquinolin-4-yl)ethane-1,2-diamine (**15**)

Acylsemicarbazide **6**: 0.170 g; additional 0.056 g (0.213 mmol) of triphenylphosphine was added to the reaction mixture and refluxed at 46 °C for another 72 h; eluent: DCM/MeOH 9:1; yield 0.050 g (31%); mp 219 °C; IR (ATR, *ν*/cm^−1^) 3350, 1619, 1581, 1548, 1494, 1375, 1332, 1146, 1024, 806, 738; ^1^H NMR (DMSO-d_6_): *δ* 8.43 (d, 1H, *J* = 6.0 Hz), 8.22 (d, 1H, *J* = 9.6 Hz), 7.92 (t, 1H, *J* = 5.4 Hz), 7.79 (d, 1H, *J* = 4.2 Hz), 7.52 (t, 1H, *J* = 5.4 Hz), 7.45 (dd, 1H, *J* = 4.2 Hz, 2.4 Hz), 7.38 (dd, 1H, *J* = 7.5, 1.2 Hz), 7.16 (dt, 1H, J = 7.8, 1.8 Hz), 6.83 (d, 1H, *J* = 4.2 Hz), 6.64 (d, 1H, *J* = 3.0 Hz), 6.60 (t, 1H, *J* = 7.2 Hz), 6.58–6.52 (bs, 2H), 3.55 (m, 4H) ppm; ^13^C NMR (DMSO-d_6_): *δ* 162.5, 158.7, 152.3, 150.6, 149.5, 147.2, 134.0, 131.4, 127.9, 126.6, 124.6, 124.5, 117.9, 115.9, 115.8, 105.6, 99.1, 41.9, 41.3 ppm; ESI-MS: *m/z* 380.93 (M + 1)^+^.

##### *N*′-(5-(2-Amino-6-fluorophenyl)-1,3,4-oxadiazol-2-yl)-*N*-(7-chloroquinolin-4-yl)ethane-1,2-diamine (**16**)

Acylsemicarbazide **7**: 0.178 g; eluent: DCM/MeOH 0–20%; yield 0.031 g (18%); mp 191 °C; IR (ATR, *ν*/cm^−1^) 3460, 3304, 1641, 1628, 1579, 1540, 1453, 1483, 1378, 1233, 1076, 1037, 998, 914, 867, 842, 810, 760; ^1^H NMR (DMSO-d_6_): *δ* 8.46 (d, 1H, *J* = 10.8 Hz), 8.30 (d, 1H, *J* = 9.6 Hz), 8.04 (t, 1H, *J* = 7.2 Hz), 8.03–7.93 (bs, 1H), 7.83 (d, 1H, *J* = 2.4 Hz), 7.53 (dd, 1H, *J* = 8.4, 1.8 Hz), 7.15 (q, 1H, *J* = 7.2 Hz), 6.85 (s, 2H), 6.71 (d, 1H, *J* = 5.4 Hz), 6.60 (d, 1H, *J* = 8.4 Hz), 6.44–6.40 (m, 1H), 3.63 (q, 2H), 3.53 (q, 2H) ppm; ^13^C NMR (DMSO-d_6_): *δ* 163.1, 161.2, 159.5, 151.9, 150.2, 149.4, 135.0, 132.0 (d, *J* = 46.2 Hz), 125.9, 125.3, 124.9, 117.5, 111.7, 101.9 (d, *J* = 87.6 Hz), 99.1, 95.1 (d, *J* = 60.0 Hz), 42.0, 41.3 ppm; ESI-MS: *m/z* 399.16 (M + 1)^+^.

##### *N*′-(5-(2-Amino-4-chlorophenyl)-1,3,4-oxadiazol-2-yl)-*N*-(7-chloroquinolin-4-yl)ethane-1,2-diamine (**17**)

Acylsemicarbazide **8**: 0.185 g; eluent: DCM/MeOH 0–20%; yield 0.044 g (25%); mp 208 °C; IR (ATR, *ν*/cm^−1^) 3489, 3363, 1641, 1619, 1579, 1537, 1488, 1446, 1331, 1265, 1145, 1026, 844, 815, 787, 770, 737; ^1^H NMR (DMSO-d_6_): *δ* 8.42 (d, 1H, *J* = 5.4 Hz), 8.20 (d, 1H, *J* = 9.6 Hz), 7.97 (t, 1H, *J* = 5.4 Hz), 7.78 (d, 1H, *J* = 1.8 Hz), 7.46 (m, 1H), 7.45 (dd, 1H, *J* = 9.6, 2.4 Hz), 7.34 (d, 1H, *J* = 9.0 Hz), 6.89 (d, 1H, *J* = 2.4 Hz), 6.83–6.78 (bs, 2H), 6.62 (m, 2H), 3.54 (m, 4H) ppm; ^13^C NMR (DMSO-d_6_): *δ* 162.6, 157.9, 152.4, 150.5, 148.6, 148.2, 135.7, 133.9, 128.2, 128.0, 124.6, 124.5, 118.0, 115.6, 114.8, 104.6, 99.1, 41.8, 41.2 ppm; ESI-MS: *m/z* 414.89 (M + 1)^+^.

##### *N*′-(5-(2-Amino-6-chlorophenyl)-1,3,4-oxadiazol-2-yl)-*N*-(7-chloroquinolin-4-yl)ethane-1,2-diamine (**18**)

Acylsemicarbazide **9**: 0.185 g; reaction time: 72 h; eluent: DCM/MeOH 0–17%; yield 0.041 g (23%); mp 210 °C; IR (ATR, *ν*/cm^−1^) 3311, 1627, 1611, 1578, 1453, 1428, 1331, 1281, 1140, 1019, 907, 852, 778, 725; ^1^H NMR (DMSO-d_6_): *δ* 8.68–8.58 (bs, 1H), 8.50 (d, 1H, *J* = 6.6 Hz), 8.41 (d, 1H, *J* = 8.4 Hz), 8.01 (t, 1H, *J* = 5.4 Hz), 7.89 (s, 1H), 7.62 (d, 1H, *J* = 9.0 Hz), 7.14 (t, 1H, *J* = 7.8 Hz), 6.82 (d, 1H, *J* = 7.2 Hz), 6.75 (d, 1H, *J* = 9.0 Hz), 6.66 (d, 1H, *J* = 8.4 Hz), 6.22–6.12 (bs, 2H), 3.70 (q, 2H, *J* = 3.6 Hz), 3.55 (q, 2H, *J* = 3.5 Hz) ppm; ^13^C NMR (DMSO-d_6_): *δ* 163.4, 154.4, 151.7, 150.7, 149.8, 148.8, 136.5, 133.5, 132.7, 131.8, 127.1, 124.2, 124.1, 117.4, 116.5, 114.1, 105.7, 98.6, 41.4, 40.9 ppm; ESI-MS: *m/z* 415.12 (M + 1)^+^.

##### *N*′-(5-(2-Amino-5-bromophenyl)-1,3,4-oxadiazol-2-yl)-*N*-(7-chloroquinolin-4-yl)ethane-1,2-diamine (**19**)

Acylsemicarbazide **10**: 0.204 g; an additional 0.056 g (0.213 mmol) of triphenylphosphine was added to the reaction mixture and refluxed at 46 °C for another 72 h; flash chromatography eluent: DCM/MeOH 0-15%; yield 0.018 g (9%); mp 213 °C; IR (ATR, *ν*/cm^−1^) 3456, 3304, 1627, 1604, 1579, 1541, 1452, 1333, 1282, 1231, 1075, 1039, 866, 841, 810, 779; ^1^H NMR (DMSO-d_6_): *δ* 8.46 (d, 1H, *J* = 6.6 Hz), 8.26 (d, 1H, *J* = 9.6 Hz), 8.00 (t, 1H, *J* = 6.0 Hz), 7.82 (d, 1H, *J* = 2.4 Hz), 7.86–7.73 (m, 1H), 7.49 (dd, 1H, *J* = 8.4, 2.4 Hz), 7.47 (d, 1H, *J* = 3.6 Hz), 7.29 (dd, 1H, *J* = 9.0, 2.4 Hz), 6.82 (d, 1H, *J* = 9.6 Hz), 6.75–6.70 (bs, 2H), 6.69 (d, 1H, *J* = 5.4 Hz), 3.60–3.55 (m, 4H) ppm; ESI-MS: *m/z* 461.0 (M + 1)^+^.

#### 3.1.8. General Procedure for the Synthesis of Oxadiazoles (**20**–**23**)

PPh_3_ (0.168 g, 0.639 mmol), TEA (0.238 mL, 0.172 g, 1.704 mmol), and carbon tetrachloride (0.091 mL, 0.105 g, 0.682 mmol) were added to the solution of an appropriate acylsemicarbazide (**11**–**14**) (0.426 mmol) in dry dichloromethane (4 mL). The reaction mixture was stirred for 18 h at 46 °C. The reaction mixture was cooled to r.t. and 30 mL of dichloromethane was added. The organic layer was extracted with water (3 × 30 mL) and evaporated under reduced pressure. The crude product was purified using column chromatography and trituration with diethyl ether.

##### *N*^1^-(5-(2-Aminophenyl)-1,3,4-oxadiazol-2-yl)-*N*^4^-(7-chloroquinolin-4-yl)butane-1,4-diamine (**20**)

Acylsemicarbazide **11**: 0.182 g; column chromatography eluent: DCM/MeOH 85:15; yield 0.128 g (74%); mp 127.0–128.0 °C; IR (ATR, *ν*/cm^−1^) 3646, 3448, 3319, 3195, 2948, 1636, 1585, 1610, 1547, 1494, 1453, 1367, 1332, 1268, 1199, 1147, 1025, 897, 870, 814, 764, 739, 645; ^1^H NMR (DMSO-d_6_): *δ* 8.40 (d, 1H, *J* = 5.5 Hz), 8.30 (d, 1H, *J* = 9.1 Hz), 7.81–7.75 (m, 2H), 7.51–7.42 (m, 3H), 7.19–7.14 (m, 1H), 6.84 (dd, 1H, *J* = 8.3, 1.1 Hz), 6.62 (t, 1H, *J* = 8.1 Hz), 6.57 (s, 2H), 6.52 (d, 1H, *J* = 5,6 Hz), 3.36–3.28 (m, 4H), 1.78–1.69 (m, 4H) ppm; ^13^C NMR (DMSO-d_6_): *δ* 162.1, 158.0, 151.2, 150.5, 148.3, 146.7, 133.7, 130.8, 126.8, 126.1, 124.2, 117.3, 115.4, 115.3, 105.2, 98.7, 42.3, 42.1, 26.5, 25.1 ppm; ESI-MS: *m/z* 409.0 (M + 1)^+^. 

##### *N*^1^-(5-(2-Amino-4-chlorophenyl)-1,3,4-oxadiazol-2-yl)-*N*^4^-(7-chloroquinolin-4-yl)butane-1,4-diamine (**21**)

Acylsemicarbazide **12**: 0.196 g; column chromatography eluent: DCM/MeOH 85:15; yield 0.115 g (62%); mp 123.0–124.5 °C; IR (ATR, *ν*/cm^−1^) 3454, 3319, 2949, 1644, 1610, 1585, 1546, 1489, 1426, 1369, 1350, 1335, 1262, 1201, 1141, 1085, 1029, 901, 864, 844, 816, 791, 764; ^1^H NMR (DMSO-d_6_): *δ* 8.40 (d, 1H, *J* = 5.5 Hz), 8.30 (d, 1H, *J* = 9.1 Hz), 7.83 (t, 1H, *J* = 5.6 Hz), 7.79 (d, 1H, *J* = 2.1 Hz), 7.50 (t, 1H, *J* = 4.9 Hz), 7.46 (dd, 1H, *J* = 9.0, 2.1 Hz), 7.41 (d, 1H, *J* = 8.5 Hz), 6.90 (d, 1H, *J* = 2.0 Hz), 6.81 (s, 2H), 6.64 (dd, 1H, *J* = 8.5, 2.0 Hz), 6.53 (d, 1H, *J* = 5.6 Hz), 3.80–3.08 (m, 4H), 1.94–1.51 (m, 4H) ppm; ^13^C NMR (DMSO-d_6_): *δ* 162.6, 157.7, 151.6, 151.0, 148.6, 148.2, 135.6, 134.2, 128.2, 127.1, 124.7, 117.7, 115.6, 114.8, 104.7, 99.1, 42.8, 42.6, 26.9, 25.5 ppm; ESI-MS: *m/z* 443.1 (M + 1)^+^.

##### *N*^1^-(5-(2-Amino-5-chlorophenyl)-1,3,4-oxadiazol-2-yl)-*N*^4^-(7-chloroquinolin-4-yl)butane-1,4-diamine (**22**)

Acylsemicarbazide **13**: 0.196 g; column chromatography eluent: DCM/MeOH 85:15; yield 0.104 g (55%); mp 125 °C; IR (ATR, *ν*/cm^−1^) 3395, 3097, 2947, 2869, 1672, 1613, 1581, 1547, 1492, 1471, 1450, 1357, 1322, 1254, 1212, 1138, 1058, 873, 721; ^1^H NMR (DMSO-d_6_): *δ* 8.39 (d, 1H, *J* = 5.5 Hz), 8.29 (d, 1H, *J* = 9.0 Hz), 7.84 (t, 1H, *J* = 5.7 Hz), 7.78 (d, 1H, *J* = 2.3 Hz), 7.48–7.44 (m, 2H), 7.39 (d, 1H, *J* = 2.5 Hz), 7.20 (dd, 1H, *J* = 8.8, 2.5 Hz), 6.87 (d, 1H, *J* = 8.8 Hz), 6.71 (s, 2H), 6.52 (d, 1H, *J* = 5.5 Hz), 3.40–3.31 (m, 4H), 1.77–1.70 (m, 4H) ppm; ^13^C NMR (DMSO-d_6_): *δ* 162.3, 156.9, 151.3, 150.4, 148.4, 145.5, 133.7, 130.5, 126.9, 124.9, 124.2, 124.2, 118.4, 117.3, 117.2, 106.3, 98.7, 42.3, 42.1, 26.5, 25.1 ppm; ESI-MS: *m/z* 443.1 (M + 1)^+^.

##### *N*^1^-(5-(2-Amino-6-chlorophenyl)-1,3,4-oxadiazol-2-yl)-*N*^4^-(7-chloroquinolin-4-yl)butane-1,4-diamine (**23**)

Acylsemicarbazide **14**: 0.196 g; column chromatography eluent: DCM/MeOH 75:25; yield 0.077 g (41%); mp 133.0–134.0 °C; IR (ATR, *ν*/cm^−1^) 3278, 2945, 2866, 1645, 1610, 1584, 1465, 1357, 1334, 1282, 1233, 1140, 1017, 900, 855, 811, 774, 726; ^1^H NMR (DMSO-d_6_): *δ* 8.41 (d, 1H, *J* = 5.7 Hz), 8.35 (d, 1H, *J* = 9.1 Hz), 7.81 (dd, 2H, *J* = 10.3, 3.9 Hz), 7.74 (s, 1H), 7.50 (dd, 1H, *J* = 9.0, 2.2 Hz), 7.14 (t, 1H, *J* = 8.1 Hz), 6.77 (dd, 1H, *J* = 7.8, 0.7 Hz), 6.56 (d, 1H, *J* = 5.8 Hz), 6.20 (s, 2H), 3.46–3.23 (m, 4H), 1.80–1.68 (m, 4H) ppm; ^13^C NMR (DMSO-d_6_): *δ* 162.9, 154.7, 151.5, 150.7, 150.2, 147.6, 134.6, 133.1, 132.2, 126.4, 125.0, 124.9, 117.6, 117.0, 114.5, 106.3, 99.1, 42.8, 42.7, 26.9, 25.6 ppm; ESI-MS: *m/z* 443.0 (M + 1)^+^.

### 3.2. Anti-QS and Bactericidal Activity Screening

*Chromobacterium violaceum* ATCC31532 (ATCC; Wesel, Germany) was used as the indicator reporter strain to screen compounds **8**, **12**, and **14**–**23** for anti-QS/biofilm or bactericidal activities [17,35,37]. Shortly, the reporter strain grown overnight at 27 °C on Luria-Bertani agar (Fischer Scientific, Leicestershire, UK) was suspended in PDYT (0.5% peptone, 0.3% D-glucose, 0.25% yeast extract, and 0.05% L-tryptophan, *m*/*v*) to achieve OD_600_ = 0.02. Then, 200 µL of the obtained cell suspension with 2% DMSO (control) or with the indicated compounds dissolved in DMSO at varying concentrations (400, 200, 100, and 40 μM) was added into the wells in two parallel 96-well plates (Tissue Culture Treated, polystyrene, flat-bottom, Becton Dickinson). Quercetin [66] and azithromycin (Sigma-Aldrich) at 400 μM dissolved in DMSO were used as positive controls for QS inhibition and cell viability (bactericidal agent), respectively. The plates were incubated at 27 °C under aerobic conditions (200 rpm) for 22 h. Resazurin, a redox-sensitive dye that is reduced to fluorescent resorufin only by viable cells, was added at 200 μM per well in the first 96-well plate to assess the bactericidal effects of the compounds [67,68]. The 96-well plates, with/without the resazurin, were shaken for an additional 30 min (pm) in the dark and then centrifuged (4000 rpm, for 20 min, 20 °C) to pellet insoluble violacein and cells. Resorufin-containing supernatants (100 µL) were transferred into a new plate and the produced/remaining fluorescence was recorded with a PerkinElmer Victor3 multilabel microtiter plate reader using excitation/emission wavelengths of 550/590 nm. Supernatants from the 96-well plate without resazurin were removed and the pelleted violacein was dissolved in 96% (*v*/*v*) ethanol. The 96-well plate was centrifuged (4000 rpm for 20 min at 20 °C) and the supernatants with soluble violacein (100 µL) were transferred into a new 96-well plate. Changes in the violacein yields were monitored at 595 nm using the PerkinElmer Victor3 reader. Both the anti-QS and bactericidal screening experiments were repeated twice with at least three technical replicates for each plate. The inhibitory action of the most potent anti-QS compounds showing minimal bactericidal activity against *C. violaceum* was validated in 96-well plates with six independent replica samples and using cells with DMSO as the control and the experimental conditions as described above. 

### 3.3. Determination of Minimum Inhibitory Concentration (MIC) and Minimum Bactericidal Concentration (MBC)

The minimum inhibitory concentration (MIC) and minimum bactericidal concentration (MBC) for *Pseudomonas aeruginosa* PAO1 (Leibnitz Institute DSMZ 1707, Braunschweig, Germany) were determined for compounds **15**, **16**, **18**, **19,** and **23** (initially dissolved in DMSO) by the broth dilution method [69]. For the MIC assays, PAO1 cells were grown overnight in Luria-Bertani (LB) broth, and then diluted in fresh LB broth to obtain a concentration of 10^5^ CFU/mL. Subsequently, the cells were divided into 200 µL aliquots in 96-plate wells, without (DMSO alone) or with serially diluted compounds (ranging from 3200 to 100 μM). Following incubation at 37 °C for 24 h, the MIC value was determined by measuring the cell density at 570 nm to indicate the minimum compound concentration that prevented visible growth. The MBC was evaluated by transferring 100 µL from the bacterial cultures with no visible growth after the MIC assay onto the LB agar plates. These plates were then incubated at 37 °C for 24 h, and the MBC was identified as the lowest concentration of the compound that showed no growth under these conditions. The method’s limit of detection is 10 CFU/mL, meaning that the initial concentration of 10^5^ CFU/mL was reduced to below 10 CFU/mL. Consequently, the MBC was defined as the lowest concentration of the compound that kills ≥ 99.9% of bacteria [70]. Both the MIC and MBC assays were conducted in triplicate, with three independent experiments and three technical replicates per experiment. 

### 3.4. Antibiofilm Assay

The effect of compounds on biofilm formation and on the eradication of pre-formed biofilm was studied on *Pseudomonas aeruginosa* PAO1 (Leibnitz Institute DSMZ 1707, Braunschweig, Germany). We performed three independent experiments with 8 technical replicates per experiment.

For the inhibition of biofilm formation, *P. aeruginosa* PAO1 was grown overnight in Luria-Bertani (LB) medium and diluted with LB medium to an optical density of 0.5 McFarland. To initiate QS-dependent biofilm formation, we performed an additional 1:100 dilution step to obtain an optical density of approximately 0.01 at 600 nm. In this second step, bacterial culture was diluted with 1% DMSO in the M63 medium with 0.4% Arg (control) or with the M63 medium with 0.4% Arg containing indicated compounds (**15**, **16**, **18**, **19,** and **23**; initially dissolved in DMSO) at 100 μM concentration, and 100 μL was transferred to the 96-well microtiter plate with a U-bottom in 8 technical replicates. Plates were incubated aerobically at 37 °C for 24 h. Biofilm was detected as described in O’Toole G. A., 2011 [71]. Bacterial growth was measured at 570 nm (Wallac Victor 2 1420, Perkin Elmer), planktonic cells were removed, and biofilm was stained with 125 μL of 0.1% crystal violet for 15 min. The microtiter plate was rinsed three times with H_2_O, dried, and biofilm was extracted using 150 μL of 30% acetic acid. An amount of 125 μL of solubilized crystal violet was transferred to the new flat-bottom microtiter plate and absorbance was measured at 540 nm (Wallac Victor 2 1420, Perkin Elmer). Biofilm index was calculated as a ratio (A540/A570) × 100. To study the effect of selected compounds (**15**, **16**, **18**, **19,** and **23**) on already formed biofilm, *P. aeruginosa* PAO1 was grown overnight in LB medium and diluted with LB medium to an optical density of 0.5 McFarland. Bacterial culture was then diluted 1:100 in M63 medium with 0.4% Arg and 100 μL was transferred to the 96-well microtiter plate with a U-bottom and incubated aerobically at 37℃ for 24 h to form biofilm. The bacterial suspension was removed, and wells were washed twice with M63 medium with 0.4% Arg. An amount of 100 μL of 1% DMSO in the M63 medium with 0.4% Arg (control) or 100 μM of indicated compounds in the M63 medium with 0.4% Arg was added to each well in 8 technical replicates. Plates were further incubated at 37 ℃ for 24 h and biofilm was stained as described above.

### 3.5. Effect on Pyocyanin Production

*P. aeruginosa* PAO1 cells were grown overnight in LB medium, adjusted to 0.5 McFarland with LB medium, and diluted 1:100 in 5 mL LB medium containing either 1% DMSO (control) or 100 μM compounds **15**, **16**, **18**, **19,** and **23**. The culture was grown aerobically for 24 h at 37 ℃ and bacterial growth was measured at A570. Pyocyanin was extracted as described in Essar et al., 1990 [72]. Cells were pelleted and pyocyanin was extracted from the supernatant with 3 mL of chloroform, centrifuged for 10 min at 5000 rpm (Thermo Jouan BR4i), and the chloroform layer was re-extracted with 1 mL of 0.2 M HCl. Absorbance was measured at 520 nm and the results were expressed as a ratio (A520/A570) × 100 to normalize pyocyanin values to cell growth. We performed three independent experiments with 2 technical replicates per experiment. 

### 3.6. Statistical Analysis

The performance of *C. violaceum*-based screening results was monitored by calculating the screening window coefficient (Z’), as described in [73]. Potency (half inhibitory concentrations, IC50) calculations were performed using GraphPad Prism version 8 (GraphPad Software Inc., San Diego, CA, USA, version 8.0) using a paired t-test (two-tailed) and 95% confidence interval as criteria. The most potent QS inhibitors against *C. violaceum* were further validated using an unpaired *t*-test with Welch’s correction (GraphPad Software), with *p* < 0.05 as the criterion to indicate significant inhibition. For the antibiofilm and antipyocyanin assays with *P. aeruginosa*, a one-way analysis of variance (1-ANOVA) with *p* < 0.05 was considered as a significant change.

## 4. Conclusions

In the present study, we applied a molecular hybridization approach to design 9 novel compounds for interrupting the PQS-QS-mediated biofilm formation and virulence in *P. aeruginosa*. The generated 1,3,4-oxadiazole hybrid compounds, incorporating 4-(2-aminoethyl/4-aminobuthyl)amino-7-chloro-quinoline and anthranilic acid scaffolds, were first pre-screened using a Gram-negative *C. violaceum*-based microscale screening system to distinguish genuine QS inhibitors from those with bactericidal effects in one single experiment. From the tested compounds, ethan-1,2-diamine 1,3,4-oxadiazoles **15**–**19** and butan-1,4-diamine 1,3,4-oxadiazole **23**, demonstrating the most promising anti-QS activity, were then tested for their ability to prevent biofilm formation, disrupt pre-formed biofilms, and inhibit pyocyanin production using *P. aeruginosa* PAO1 as the model. Of these five anti-QS agents, ethan-1,2-diamine 1,3,4-oxadiazole **15** demonstrated the greatest anti-QS/biofilm effect (reducing biofilm formation by 50%) accompanied by some biofilm eradication activity (reducing the biomass by 25%), while showing only a minor effect on cell growth. Butan-1,4-diamine 1,3,4-oxadiazole **23,** with a marginal antibiofilm effect, proved to be the most efficient antivirulence compound by inhibiting the pyocyanin production by more than 70%. *P. aeruginosa* harbors four closely interactive QS systems, including the PQS-QS system, which explains why our compounds were not able to completely prevent biofilm formation or pyocyanin production in the PAO1 biofilm model. Thus, based on these findings, we suggest that expanding the chemical library around these two most active compounds (**15** and **23**) and considering the AIs used by other interactive QS systems used by *P. aeruginosa* is needed to establish a reliable SAR and to obtain a maximal anti-QS effect against this clinically important pathogen.

## Figures and Tables

**Table 1:** Quorum sensing inhibition and bactericidal effect of acylsemicarbazide (**8**, **12** and **14**) and 1,3,4-oxadiazole (**15**–**23**) derivatives at 400 μM concentrations on the *C. violaceum* ATCC 31532.

Compd.	QSI (%)	Bactericidal Effect (%)
**8**	38.6 ± 0.9	7.2 ± 0.0
**12**	ne	2.9 ± 0.1
**14**	ne	1.8 ± 0.2
15 *	87.4 ± 3.6	52.9 ± 6.3
16 *	90.5 ± 2.8	60.6 ± 2.5
17 *	89.6 ± 0.4	84.0 ± 4.8
18 *	89.3 ± 0.8	71.4 ± 2.8
19 *	85.8 ± 0.9	46.0 ± 7.1
**20**	46.5 ± 9.7	10.6 ± 7.8
**21**	53.7 ± 5.5	7.6 ± 2.7
**22**	38.6 ± 0.7	2.6 ± 3.6
23 *	83.5 ± 0.03	46.9 ± 2.1
**Q**	95.8 ± 0.4	9.8 ± 4.7
**AZ**	95.9 ± 0.2	89.1 ± 1.5
**F267**	56.8 ± 7.7	13.1 ± 5.3
**F2896**	62.1 ± 5.6	17.8 ± 5.6

QSI—quorum sensing inhibition; Q—quercetin; AZ—azithromycin; ne—no effect; F267 and F2896—previously identified flavonols with demonstrated anti-QS effects [37,38]. *, compounds showing significant change (unpaired *t*-test with Welch’s correction, *p* < 0.05) in comparison to control cells with DMSO.

**Table 2:** Parameters of cell growth inhibition, inhibition of biofilm formation, biofilm eradication, pyocyanin inhibition, and biofilm index of *P. aeruginosa* PAO1 cells treated with 100 μM compounds compared to control cells treated with 1% DMSO.

Compd.	GI (%)	BFI (%)	Biofilm Index	BE (%)	PI (%)
Ø *			60.25		
15 *	8.13 ± 2.76	48.6 ± 1.81	33.72	24.86 ± 1.13	42.34 ± 1.37
16 *	31.85 ± 1.64	53.72 ± 0.43	40.9	43.08 ± 2.15	20.45 ± 0.69
18 *	28.34 ± 2.57	45.26 ± 1.88	46.02	29.74 ± 1.47	16.02 ± 0.87
19 *	43.7 ± 2.24	61.57 ± 0.84	41.13	35.36 ± 1.62	38.87 ± 1.57
23 *	23.38 ± 3.03	29.08 ± 1.03	55.75	10.89 ± 1.29	72.02 ± 1.25

Ø—1% DMSO; GI—growth inhibition (A570); BFI—biofilm formation inhibition (A540); biofilm index—(A540/A570) × 100; BE—biofilm eradication (A540); PI—pyocyanin inhibition—(A520/A570) × 100. *, compounds showing statistically significant change in comparison to the control cells with DMSO (1-ANOVA, *p* < 0.05).

## Data Availability

The data presented in this study are available in this article and in the Supplementary Materials.

## Supplementary Materials

The following supporting information can be downloaded at https://www.mdpi.com/article/10.3390/molecules28155866/s1, Table S1. ^1^H and ^13^C NMR spectroscopic data for acylsemicarbazides; Table S2. ^1^H and ^13^C NMR spectroscopic data for 1,3,4-oxadiazoles; Figure S1. IR, MS and/or NMR spectra of compounds **2**, **4a**, **5a**, **5d**, **6**, **8**, **9**, **11**, **12**, **14–23**.

## Sample Availability

Samples of the compounds are available from the authors.
