# Supplementary material for: Synthesis and Biological Evaluation of New Quinoline and Anthranilic Acid Derivatives as Potential Quorum Sensing Inhibitors
Source: Molecules. 2023 Aug 3;28(15):5866. doi: 10.3390/molecules28155866 (PMC10420644; doi:10.3390/molecules28155866)
Supplement: Supplementary file 1 [file molecules-28-05866-s001.zip › molecules-2516938-supplementary.pdf]

Supplementary Materials

Synthesis and Biological Evaluation of New Quinoline and Anthranilic Acid Derivatives as Potential Quorum Sensing Inhibitors

Ivana Perković [1,*], Tanja Poljak [2], Kirsi Savijoki [3,*], Pekka Varmanen [3], Gordana Maravić-Vlahoviček [1], Maja Beus [1], Anja Kučević [1], Ivan Džajić [4] and Zrinka Rajić [1]

[1] Faculty of Pharmacy and Biochemistry, University of Zagreb, 10000 Zagreb, Croatia; gmaravic@pharma.hr (G.M.-V.); mbeus@pharma.hr (M.B.); anja.kucevic@proton.me (A.K.); zrajic@pharma.hr (Z.R.)
[2] Selvita Ltd., 10000 Zagreb, Croatia; tanja.poljak@selvita.com
[3] Department of Food and Nutrition, Faculty of Agriculture and Forestry, University of Helsinki,
00014 Helsinki, Finland; pekka.varmanen@helsinki.fi
[4] Faculty of Pharmacy, University of Ljubljana, 1000 Ljubljana, Slovenia; ivan.dzajic@ffa.uni-lj.si
* Correspondence: iperkovic@pharma.hr (I.P.); kirsi.savijoki@helsinki.fi (K.S.)

Table S1. ^{1}H and ^{13}C NMR spectroscopic data for acylsemicarbazides.

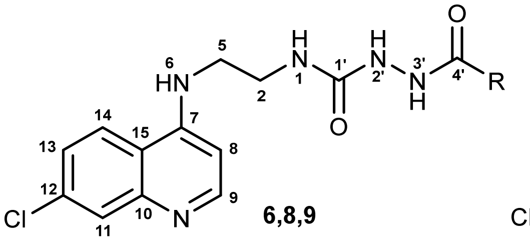

6,8,9

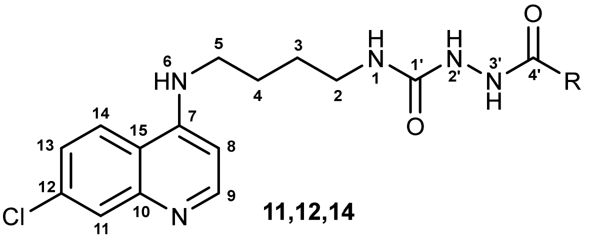

11,12,14

Compd.	R	^{1}H NMR (DMSO-d_6, δ ppm, J/Hz)	^{13}C NMR (DMSO-d_6, δ ppm)
**6**	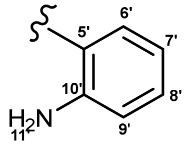	9.93–9.72 (bs, 1H, 3′), 8.40 (d, 1H, J = 5.2 Hz, 9), 8.16 (d, 1H, J = 8.8 Hz, 14), 8.05 – 7.92 (bs, 1H, 2′), 7.79 (d, 1H, J = 2.8 Hz, 11), 7.60 (d, 1H, J = 8.4 Hz, 6′), 7.43 (dd, 1H, J = 10.4, 2.4 Hz, 13), 7.48–7.36 (m, 1H, 6), 7.18 (dt, 1H, J = 8.4, 1.2 Hz, 8′), 6.86–6.75 (bs, 1H, 1), 6.72 (d, 1H, J = 6.00 Hz, 9′), 6.57 (d, 1H, J = 5.6 Hz, 8), 6.51 (t, 1H, J = 7.6 Hz, 7′), 6.44–6.32 (bs, 2H, 11′), 3.40–3.25 (m, 4H, 2, 5)	168.9 (4′), 159.3 (1′), 152.0 (9), 150.1 (7), 150.0 (10), 149.0 (10′), 133.4 (12), 132.2 (8′), 128.4 (11), 127.5 (6′), 124.1 (13), 123.8 (14), 117.3 (15), 116.3 (9′), 114.4 (7′), 112.4 (5′), 98.6 (8), 43.4 (2), 37.8 (5)
**8**	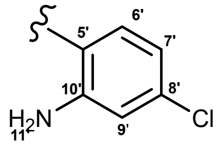	10.00–9.87 (bs, 1H, 3′), 8.41 (d, 1H, J = 5.4 Hz, 9), 8.16 (d, 1H, J = 8.4 Hz, 14), 8.02 (s, 1H, 2′), 7.79 (d, 1H, J = 2.4 Hz, 11), 7.61 (d, 1H, J = 9,0 Hz, 6′), 7.44 (dd, 1H, J = 9.6, 2.4 Hz, 13), 7.41 (t, 1H, J = 4.8 Hz, 6), 6.88-6.80 (bs, 1H, 1), 6.79 (d, 1H, J = 2.4 Hz, 9′), 6.73-6.65 (bs, 2H, 1′), 6.56 (d, 1H, J = 5.4 Hz, 8), 6.54 (dd, 1H, J = 9.0, 1.8 Hz, 7′), 3.38-3.27 (m, 4H, 2, 5)	168.2 (4′), 159.2 (1′), 152.0 (9), 151.2 (10), 150.1 (7), 149.0 (10′), 136.7 (8′), 133.4 (12), 130.3 (6′), 127.5 (11), 124.1 (13), 123.8 (14), 117.3 (15), 115.0 (9′), 114.1 (7′), 111.2 (5′), 98.6 (8), 43.3 (2), 37.8 (5)
**9**	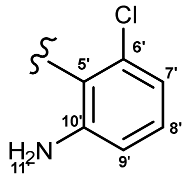	10.08–9.85 (bs, 1H, 3′), 8.56 (s, 1H, 2′), 8.41 (d, 1H, J = 6.0 Hz, 9), 8.18 (d, 1H, J = 9.0 Hz, 14), 7.79 (d, 1H, J = 2.0 Hz, 11), 7.46 (dd, 1H, J = 4.5, 2.0 Hz, 13), 7.40 (t, 1H, J = 5.0 Hz, 6), 7.05 (t, 1H, J = 7.5 Hz, 1), 6.63 (d, 1H, J = 9.0 Hz, 9′), 6.63 (s, 1H, 8′), 6.56 (d, 1H, J = 5.0 Hz, 8), 6.54 (d, 1H, J = 6.5 Hz, 7′), 5.99 (s, 2H, 11′), 3.46-3.41 (m, 4H, 2, 5)	165.9 (4′), 159.2 (1′), 152.0 (9), 150.0 (10), 149.0 (10′), 147.9 (7), 133.4 (12), 130.6 (8′), 130.5 (6′), 127.5 (11), 124.2 (13), 123.9 (14), 119.1 (5′), 117.3 (15), 115.1 (7′), 113.2 (9′), 98.7 (8), 43.3 (2), 38.1 (5)
**11**	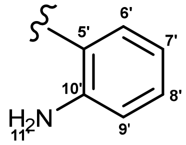	9.78 (s, 1H, 2′), 8.38 (d, 1H, J = 5,4 Hz, 3′), 8.28 (d, 1H, J = 9.0 Hz, 9), 7.77 (d, 1H, J = 2.2 Hz, 14), 7.69 (s, 1H, 11), 7.58 (d, 1H, J = 7.9 Hz, 6′), 7.43 (dd, 1H, J = 9.0, 2.3 Hz, 13), 7.34 (t, 1H, J = 5.4 Hz, 6), 7.20 – 7.13 (m, 1H, 8′), 6.70 (dd, 1H, J = 8.4, 1.2 Hz, 8), 6.52 – 6.46 (m, 3H, 1, 7′, 9′), 6.41 (s, 2H, 11′), 3.28 (q, 2H, J = 7,1 Hz, 5), 3.10 (q, 2H, J = 6.6 Hz, 2), 1.66 (q, 2H, J = 7.3 Hz, 4), 1.53 (q, 2H, J = 7.1 Hz, 3)	168.9 (4′), 158.7 (1′), 151.9 (9), 150.1 (7), 149.8 (10), 149.0 (10′), 133.3 (12), 132.1 (8′), 128.4 (11), 127.4 (6′), 124.1 (13), 124.0 (14), 117.4 (15), 116.2 (9′), 114.4 (7′), 112.7 (5′), 98.7 (8), 42.2 (2), 38.9 (5), 27.5 (4), 25.1 (3)

**12**	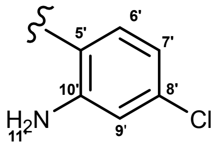	9.86 (s, 1H, 3′), 8.39 (d, 1H, *J* = 5,4 Hz, 9), 8.29 (d, 1H, *J* = 9.1 Hz, 14), 7.78 (d, 1H, *J* = 2.2 Hz, 11), 7.72 (s, 1H, 2′), 7.58 (d, 1H, *J* = 8.5 Hz, 6′), 7.44 (dd, 1H, *J* = 9.0, 2.3 Hz, 13), 7.42 (t, 1H, *J* = 5.3 Hz, 6), 6.77 (d, 1H, *J* = 2.2 Hz), 6.66 (s, 2H, 11′), 6.52 (dd, 2H, *J* = 8.5, 2.2 Hz, 9′), 6.49 (d, 1H, *J* = 5.5 Hz, 8), 3.29 (q, 2H, *J* = 7,1 Hz, 5), 3.09 (q, 2H, *J* = 6.6 Hz, 2), 1.69 – 1.62 (m, 2H, 4), 1.56 – 1.49 (m, 2H, 3)	168.1 (4′), 158.6 (1′), 151.5 (9), 151.1 (10), 150.3 (7), 148.6 (10′), 136.6 (8′), 133.5 (12), 130.3 (6′), 127.1 (11), 124.2 (13), 124.1 (14), 117.4 (15), 115.0 (9′), 114.1 (7′), 111.5 (5′), 98.7 (8), 42.2 (2), 38.9 (5), 27.6 (4), 25.1 (3)
**14**	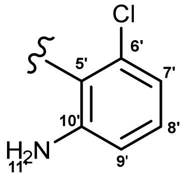	9.92 (s, 1H, 3′), 8.39 (d, 1H, *J* = 5,4 Hz, 9), 8.28 (d, 1H, *J* = 9.1 Hz, 14), 8.17 (s, 1H, 2′), 7.77 (d, 1H, *J* = 2.2 Hz, 11), 7.42 (dd, 1H, *J* = 9.0, 2.3 Hz, 13), 7.32 (t, 1H, *J* = 5.4 Hz, 6), 7.04 (t, 1H, *J* = 8.1 Hz, 8′), 6.60 (dd, 1H, *J* = 8.3, 1.0 Hz, 7′), 6.54 (dd, 2H, *J* = 7.8, 0.9 Hz, 9′), 6.49 (d, 1H, *J* = 5.5 Hz, 8), 6.35 (t, 1H, *J* = 5.8 Hz, 1), 5.93 (s, 2H, 11′), 3.29 (q, 2H, *J* = 7,1 Hz, 5), 3.13 (q, 2H, *J* = 6.6 Hz, 2), 1.72 – 1.64 (m, 2H, 4), 1.59 – 1.51 (m, 2H, 3)	166.0 (4′), 158.6 (1′), 152.0 (9), 150.1 (7), 149.1 (10′), 147.9 (10), 133.3 (12), 130.6 (8′), 130.6 (6′), 127.5 (11), 124.1 (13), 124.0 (14), 119.1 (5′), 117.5 (15), 115.1 (7′), 113.2 (9′), 98.7 (8), 42.1 (2), 39.1 (5), 27.5 (4), 25.1 (3)

Table S2. ^{1}H and ^{13}C NMR spectroscopic data for 1,3,4-oxadiazoles.

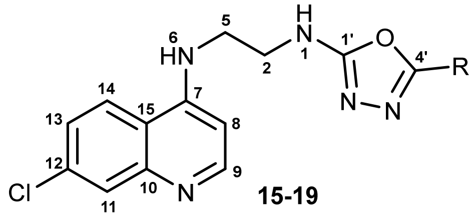

15-19

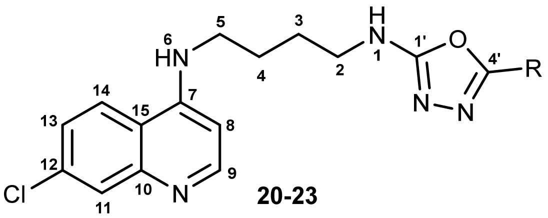

20-23

Compd.	R	^{1}H NMR (DMSO-d_6, δ ppm, J/Hz)	^{13}C NMR (DMSO-d_6, δ ppm)
**15**	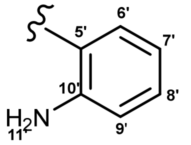	8.43 (d, 1H, J = 6.0 Hz, 9), 8.22 (d, 1H, J = 9.6 Hz, 14), 7.92 (t, 1H, J = 5.4 Hz, 1), 7.79 (d, 1H, J = 4.2 Hz, 11), 7.52 (t, 1H, J = 5.4 Hz, 6), 7.45 (dd, 1H, J = 4.2Hz, 2.4 Hz, 13), 7.38 (dd, 1H, J = 7.5, 1.2 Hz, 6′), 7.16 (dt, 1H, J = 7.8, 1.8 Hz, 8′), 6.83 (d, 1H, J = 4.2 Hz, 9′), 6.64 (d, 1H, J = 3.0 Hz, 7′), 6.60 (t, 1H, J = 7.2 Hz, 7′), 6.58 – 6.52 (bs, 2H, 11′), 3.55 (m, 4H, 2, 5)	162.5 (1′), 158.7 (4′), 152.3 (9), 150.6 (10′), 149.5 (10), 147.2 (7), 134.0 (12), 131.4 (8′), 127.9 (11), 126.6 (6′), 124.6 (13), 124.5 (14), 117.9 (15), 115.9 (9′), 115.8 (7′), 105.6 (5′), 99.1 (8), 41.9 (2), 41.3 (5)
**16**	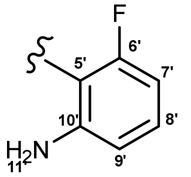	8.46 (d, 1H, J = 10.8 Hz, 9), 8.30 (d, 1H, J = 9.6 Hz, 14), 8.04 (t, 1H, J = 7.2 Hz, 1), 8.03 – 7.93 (bs, 1H, 8′), 7.83 (d, 1H, J = 2.4 Hz, 11), 7.53 (dd, 1H, J = 8.4, 1.8 Hz, 13), 7.15 (q, 1H, J = 7.2 Hz, 6), 6.85 (s, 2H, 11′), 6.71 (d, 1H, J = 5.4 Hz, 7′), 6.60 (d, 1H, J = 8.4 Hz, 8), 6.44 – 6.40 (m, 1H, 9′), 3.63 (q, 2H, 2), 3.53 (q, 2H, 5)	163.1 (1′), 161.2 (6′), 159.5 (4′), 155.4 (7), 151.9 (10), 150.2 (9), 149.4 (10′), 135.0 (12), 132.0 (d, J = 46.2 Hz, 8′), 125.9 (11), 125.3 (13), 124.9 (14), 117.5 (15), 111.7 (9′), 101.9 (d, J = 87.6 Hz, 7′), 99.1 (8), 95.1 (d, J = 60.0 Hz, 5′), 42.0 (2), 41.3 (5)
**17**	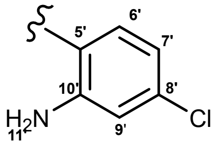	8.42 (d, 1H, J = 5.4 Hz, 9), 8.20 (d, 1H, J = 9.6 Hz, 14), 7.97 (t, 1H, J = 5.4 Hz, 1), 7.78 (d, 1H, J = 1.8 Hz, 11), 7.46 (m, 1H, 6), 7.45 (dd, 1H, J = 9.6, 2.4 Hz, 13), 7.34 (d, 1H, J = 9.0 Hz, 6′), 6.89 (d, 1H, J = 2.4 Hz, 7′), 6.83–6.78 (bs, 2H, 11′), 6.62 (m, 2H, 8, 9′), 3.54 (m, 4H, 2, 5)	162.6 (1′), 157.9 (4′), 152.4 (9), 150.5 (10′), 148.6 (10), 148.2 (7), 135.7 (8′), 133.9 (12), 128.2 (6′), 128.0 (11), 124.6 (13), 124.5 (14), 118.0 (15), 115.6 (9′), 114.8 (7′), 104.6 (5′), 99.1 (8), 41.8 (2), 41.2 (5)
**18**	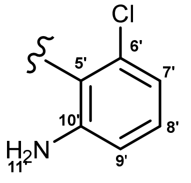	8.68–8.58 (bs, 1H, 13), 8.50 (d, 1H, J = 6.6 Hz, 9), 8.41 (d, 1H, J = 8.4 Hz, 14), 8.01 (t, 1H, J = 5.4 Hz, 1), 7.89 (s, 1H, 11), 7.62 (d, 1H, J = 9.0 Hz, 7′), 7.14 (t, 1H, J = 7.8 Hz, 6), 6.82 (d, 1H, J = 7.2 Hz, 8′), 6.75 (d, 1H, J = 9.0 Hz, 9′), 6.66 (d, 1H, J = 8.4 Hz, 8), 6.22–6.12 (bs, 2H, 11′), 3.70 (q, 2H, J = 3.6 Hz, 2), 3.55 (q, 2H, J = 3.5 Hz, 5)	163.4 (1′), 154.4 (4′), 151.7 (9), 150.7 (7), 149.8 (10′), 148.8 (10), 136.5 (8′), 133.5 (12), 132.7 (6′), 131.8 (9′), 127.1 (11), 124.2 (13), 124.1 (14), 117.4 (5′), 116.5 (15), 114.1 (7′), 105.7 (5′), 98.6 (8), 41.4 (2), 40.9 (5)

**19**	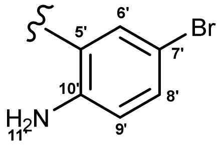	8.46 (d, 1H, *J* =6.6 Hz, 9), 8.26 (d, 1H, *J* = 9.6 Hz, 14), 8.00 (t, 1H, *J* = 6.0 Hz, 1), 7.82 (d, 1H, *J* = 2.4 Hz, 11), 7.86–7.73 (m, 1H, 6), 7.49 (dd, 1H, *J* = 8.4, 2.4 Hz, 13), 7.47 (d, 1H, *J* = 3.6 Hz, 6′), 7.29 (dd, 1H, *J* =9.0, 2.4 Hz, 8′), 6.82 (d, 1H, *J* = 9.6 Hz, 8), 6.75–6.70 (bs, 2H, 11′), 6.69 (d, 1H, *J* = 5.4 Hz, 9′), 3.60–3.55 (m, 4H, 2, 5)	na
**20**	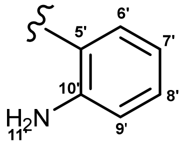	8.40 (d, 1H, *J* = 5.5 Hz, 9), 8.30 (d, 1H, *J* = 9.1 Hz, 14), 7.81 – 7.75 (m, 2H, 1, 11), 7.51 – 7.42 (m, 3H, 6, 13, 6′), 7.19 – 7.14 (m, 1H, 9′), 6.84 (dd, 1H, *J* = 8.3, 1.1 Hz, 7′), 6.62 (t, 1H, *J* = 8.1 Hz, 8′), 6.57 (s, 2H, 11′), 6.52 (d, 1H, *J* = 5,6 Hz, 8), 3.36 – 3.28 (m, 4H, 2, 5), 1.78 – 1.69 (m, 4H, 3, 4)	162.1 (1′), 158.0 (4′), 151.2 (9), 150.5 (7), 148.3 (10), 146.7 (10′), 133.7 (12), 130.8 (8′), 126.8 (11), 126.1 (6′), 124.2 (13, 14), 117.3 (15), 115.4 (9′), 115.3 (7′), 105.2 (5′), 98.7 (8), 42.3 (2), 42.1 (5), 26.5 (4), 25.1 (3)
**21**	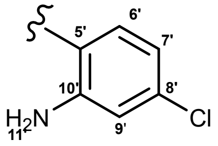	8.40 (d, 1H, *J* = 5.5 Hz, 9), 8.30 (d, 1H, *J* = 9.1 Hz, 14), 7.83 (t, 1H, *J* = 5.6 Hz, 1), 7.79 (d, 1H, *J* = 2.1 Hz, 11), 7.50 (t, 1H, J = 4.9 Hz, 6), 7.46 (dd, 1H, *J* = 9.0, 2.1 Hz, 13), 7.41 (d, 1H, *J* = 8.5 Hz, 6′), 6.90 (d, 1H, *J* = 2.0 Hz, 7′), 6.81 (s, 2H, 11′), 6.64 (dd, 1H, *J* = 8.5, 2.0 Hz, 9′), 6.53 (d, 1H, *J* = 5.6 Hz, 8), 3.80 – 3.08 (m, 4H, 2, 5), 1.94 – 1.51 (m, 4H, 3, 4)	162.6 (1′), 157.7 (4′), 151.6 (9), 151.0 (10′), 148.6 (10), 148.2 (7), 135.6 (8′), 134.2 (12), 128.2 (6′), 127.1 (11), 124.7 (13, 14), 117.7 (15), 115.6 (9′), 114.8 (7′), 104.7 (5′), 99.1 (8), 42.8 (2), 42.6 (5), 26.9 (4), 25.5 (3)
**22**	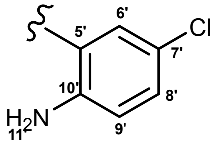	8.39 (d, 1H, *J* = 5.5 Hz, 9), 8.29 (d, 1H, *J* = 9.0 Hz, 14), 7.84 (t, 1H, *J* = 5.7 Hz, 1), 7.78 (d, 1H, *J* = 2.3 Hz, 11), 7.48 – 7.44 (m, 2H, 6, 13), 7.39 (d, 1H, *J* = 2.5 Hz, 6′), 7.20 (dd, 1H, *J* = 8.8, 2.5 Hz, 8′), 6.87 (d, 1H, *J* = 8.8 Hz, 9′), 6.71 (s, 2H, 11′), 6.52 (d, 1H, *J* = 5.5 Hz, 8), 3.40 – 3.31 (m, 4H, 2, 5), 1.77 – 1.70 (m, 4H, 3, 4)	162.3 (1′), 156.9 (4′), 151.3 (9), 150.4 (10′), 148.4 (10), 145.5 (7), 133.7 (12), 130.5 (8′), 126.9 (11), 124.9 (6′), 124.2 (8′), 124.2 (13, 14), 118.4 (7′), 117.3 (9′), 117.2 (15), 106.3 (5′), 98.7 (8), 42.3 (2), 42.1 (5), 26.5 (4), 25.1 (3)
**23**	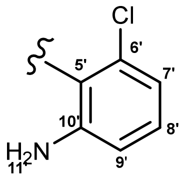	8.41 (d, 1H, *J* = 5.7 Hz, 9), 8.35 (d, 1H, *J* = 9.1 Hz, 14), 7.81 (dd, 2H, *J* = 10.3, 3.9 Hz, 11, 1), 7.74 (s, 1H, 6), 7.50 (dd, 1H, *J* = 9.0, 2.2 Hz, 13), 7.14 (t, 1H, *J* = 8.1 Hz, 8′), 6.77 (dd, 1H, *J* = 7.8, 0.7 Hz, 7′), 6.68 (dd, 1H, *J* = 7.8, 0.7 Hz, 9′), 6.56 (d, 1H, *J* = 5.8 Hz, 8), 6.20 (s, 2H, 11′), 3.46 – 3.23 (m, 4H, 2, 5), 1.80 – 1.68 (m, 4H, 3, 4)	162.9 (4′), 154.7 (1′), 151.5 (9), 150.7 (10), 150.2 (7), 147.6 (10), 134.7 (8′), 133.1 (12), 132.2 (6′), 126.4 (11), 125.0 (13), 124.9 (14), 117.6 (9′), 117.0 (15), 114.5 (7′), 106.3 (5′), 99.1 (8), 42.8 (2), 42.7 (5), 26.9 (3), 25.6 (4)

na-not available

Figure S1. IR, MS and/or NMR spectra of compounds 2, 4a, 5a, 5d, 6, 8, 9, 11, 12, 14–23.

2

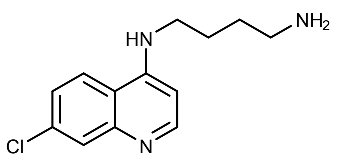

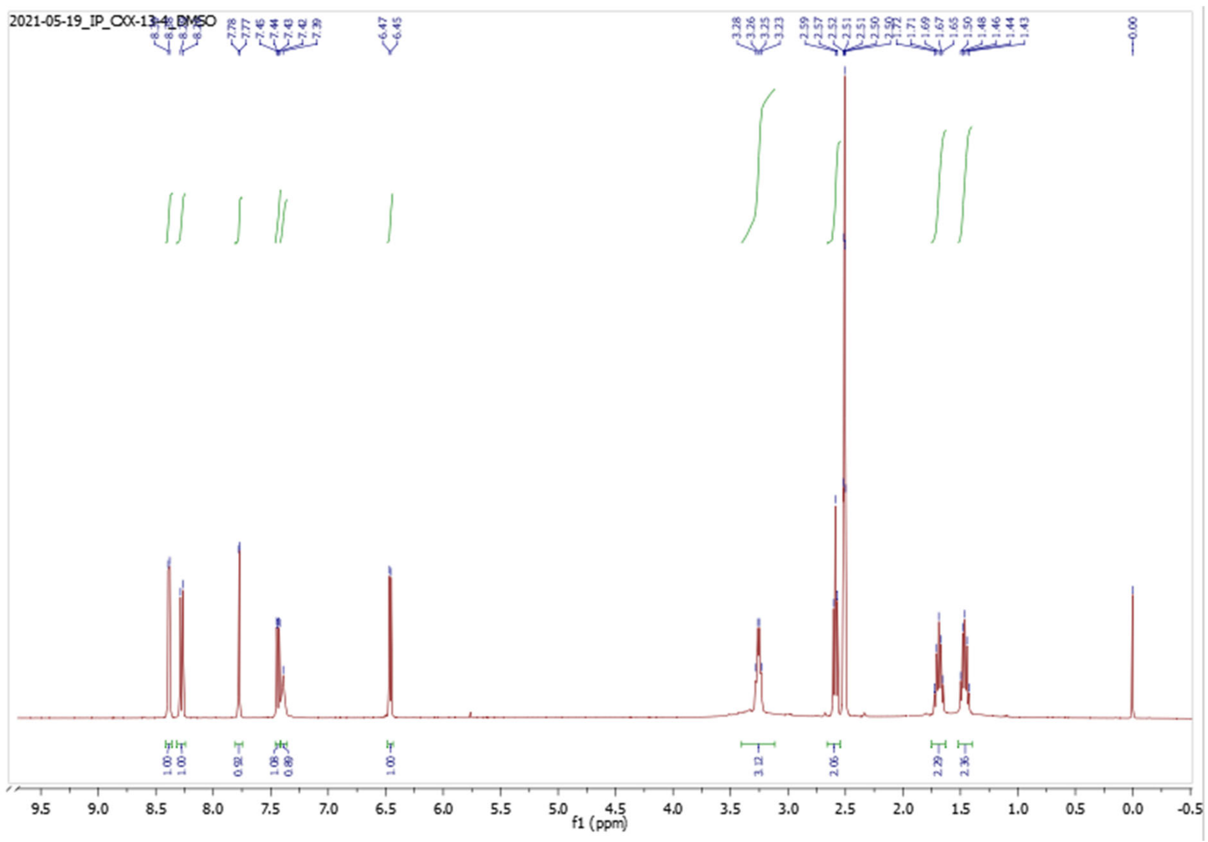

4a

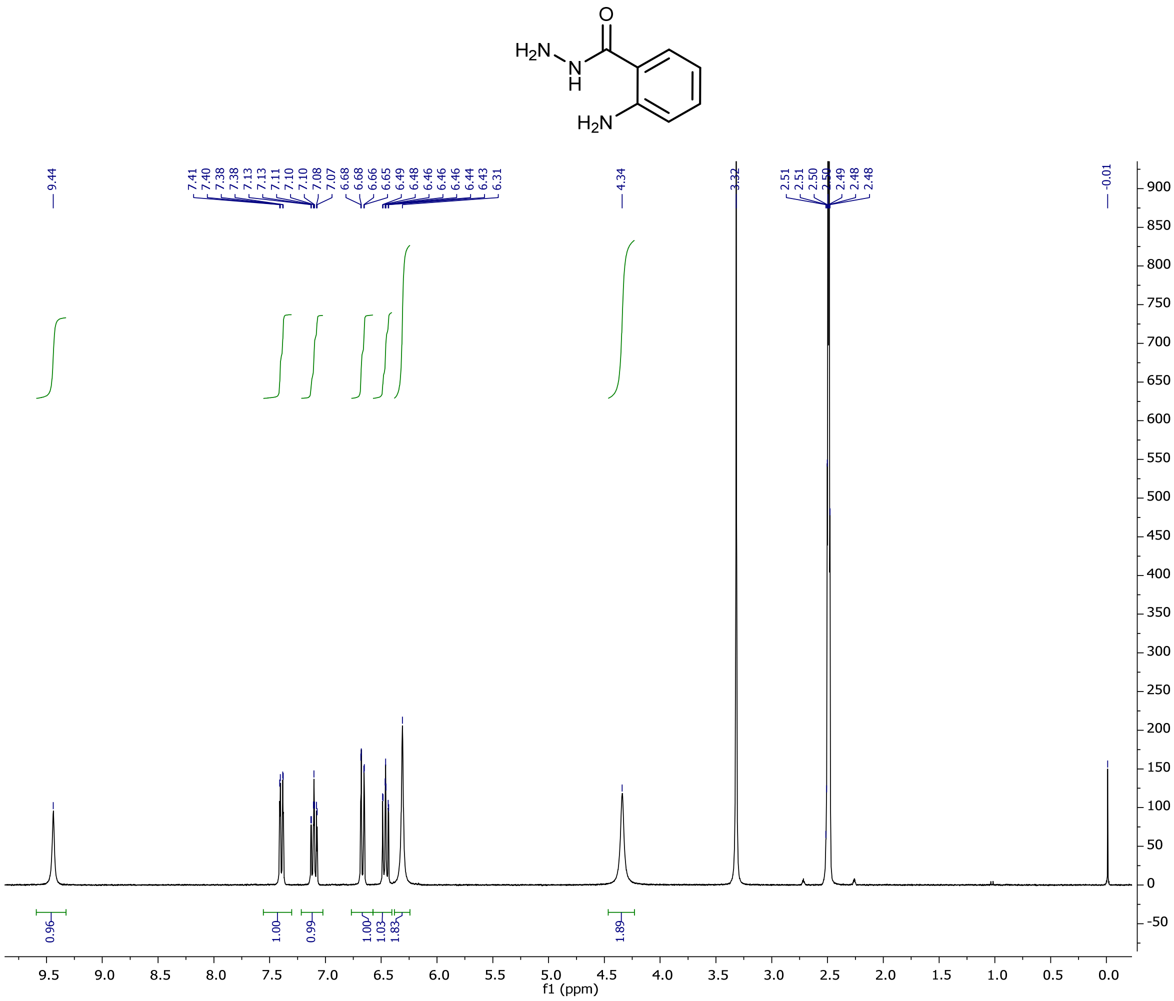
H2N
N
H
O
H2N
9.44
7.41
7.40
7.38
7.38
7.13
7.13
7.11
7.10
7.10
7.08
7.07
6.68
6.68
6.66
6.65
6.49
6.48
6.46
6.46
6.46
6.44
6.43
6.31
4.34
3.32
2.51
2.51
2.50
2.50
2.49
2.48
2.48
-0.01
0.96
1.00
0.99
1.00
1.03
1.83
1.89
f1 (ppm)

5a

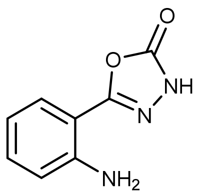
O
O
NH
N
NH2

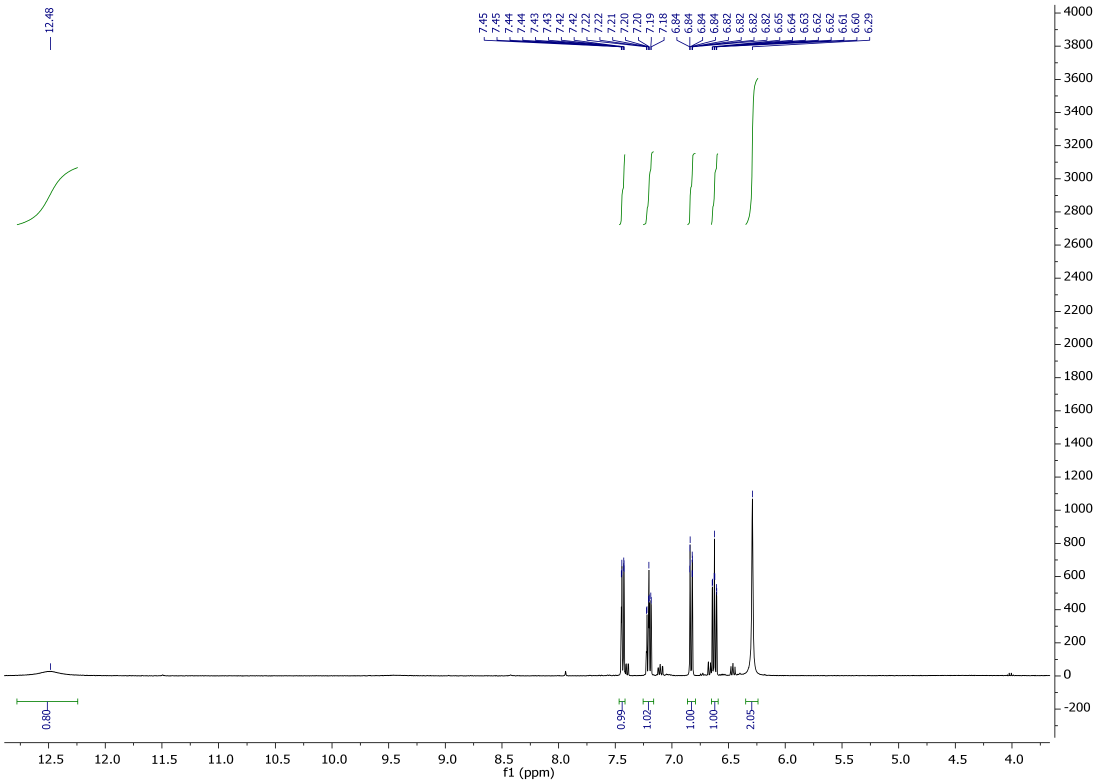
12.48
7.45
7.45
7.44
7.43
7.43
7.42
7.42
7.22
7.22
7.21
7.20
7.20
7.19
7.18
6.84
6.84
6.84
6.84
6.82
6.82
6.82
6.82
6.65
6.64
6.63
6.62
6.62
6.61
6.60
6.29
0.80
0.99
1.02
1.00
1.00
2.05
f1 (ppm)

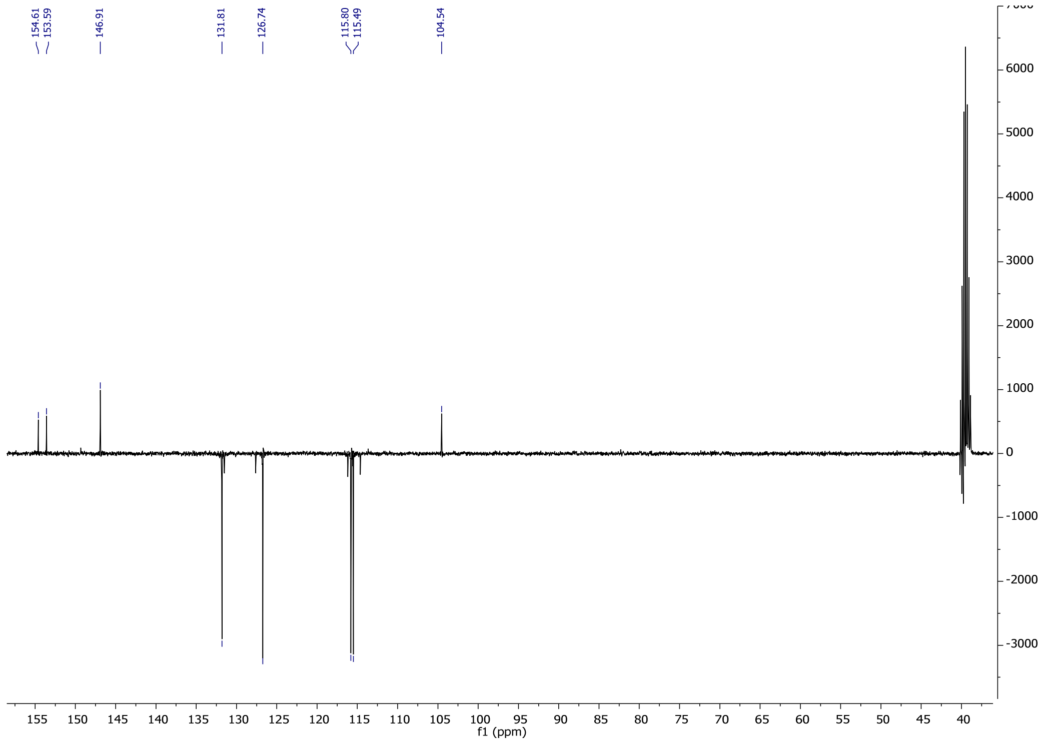
154.61
153.59
146.91
131.81
126.74
115.80
115.49
104.54
f1 (ppm)

5d

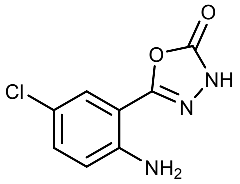
O
O
NH
N
Cl
NH2

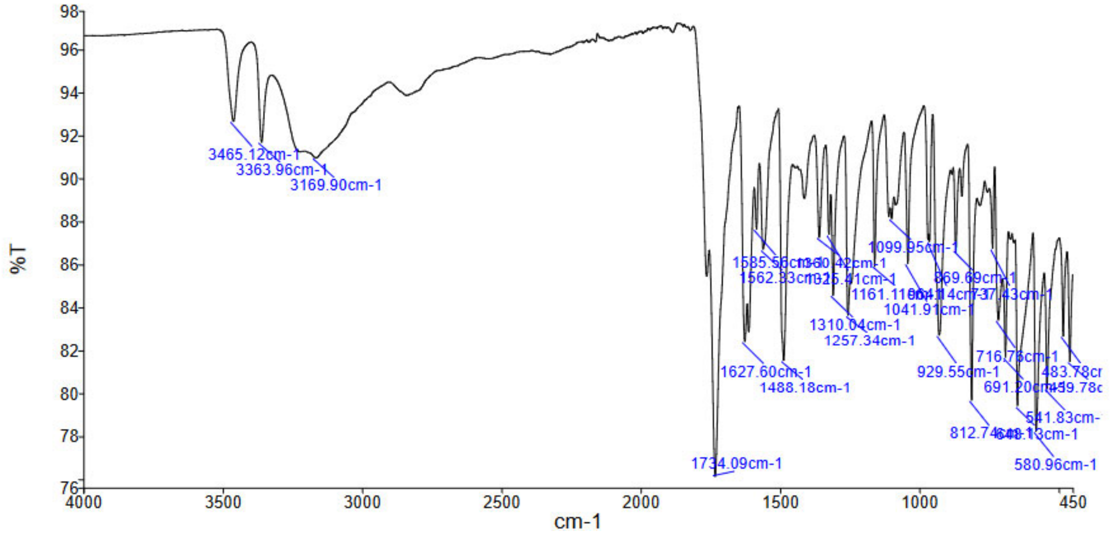
%T
cm-1
1734.09cm-1
1627.60cm-1
1488.18cm-1
3465.12cm-1
3363.96cm-1
3169.90cm-1
1310.04cm-1
1257.34cm-1
929.55cm-1
1099.95cm-1
1041.91cm-1
716.76cm-1
580.96cm-1

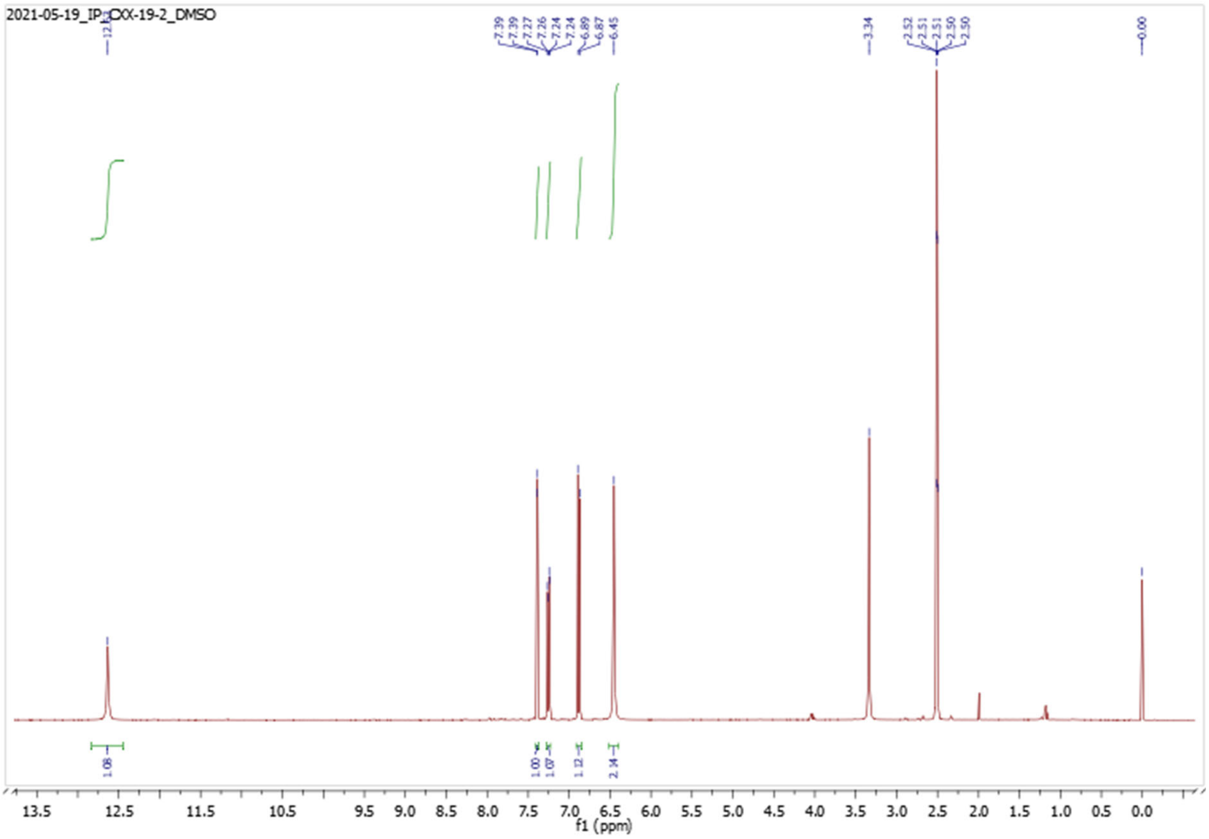
2021-05-19_IP_CXX-19-2_DMSO
f1 (ppm)

6

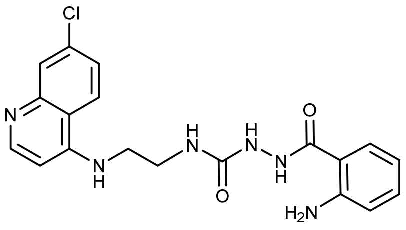

Peak ID	Compound	Time	Mass Found
1		0.61	

SAMPLE: 2:13 Combine (68:80-(50:55+93:98))

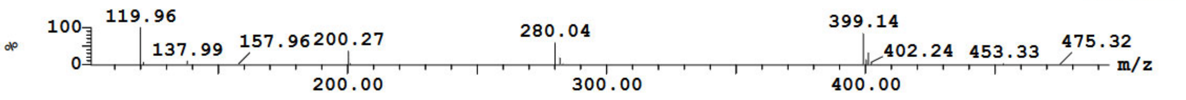

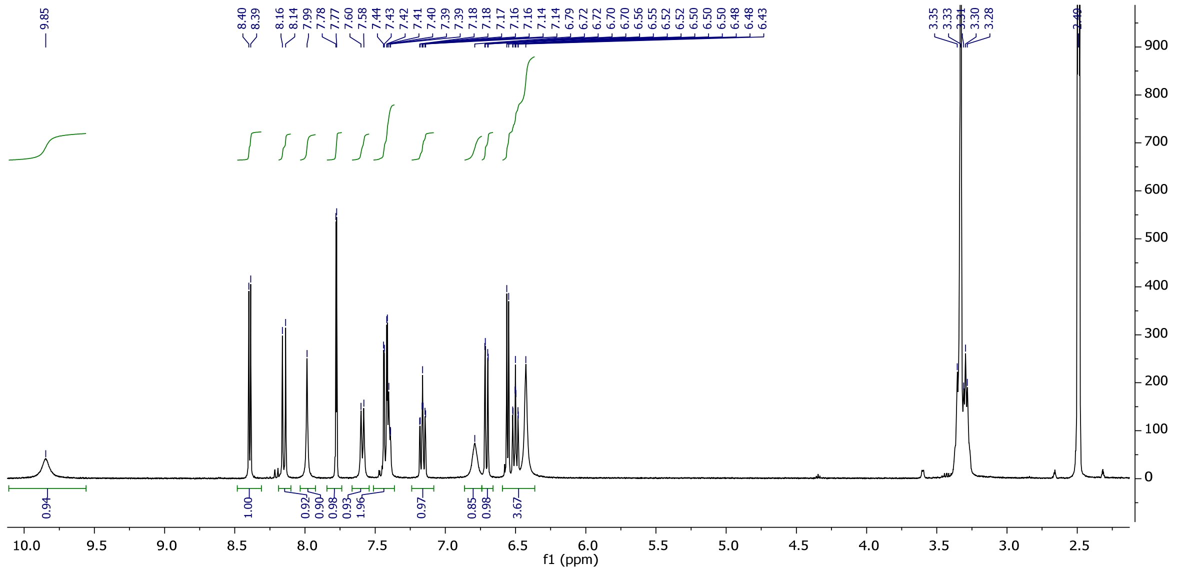

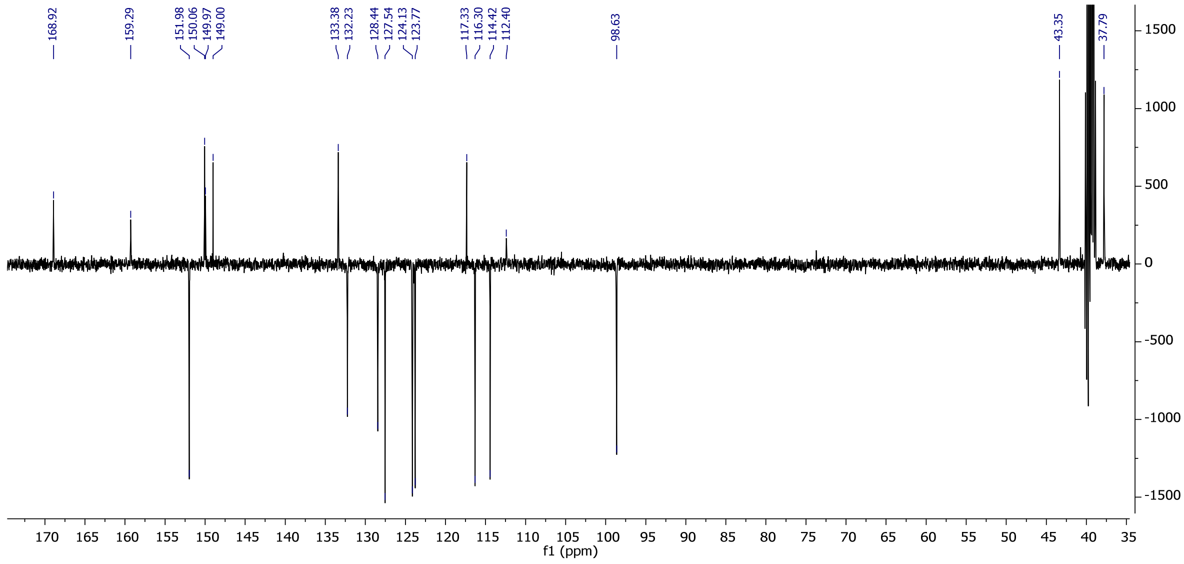

8

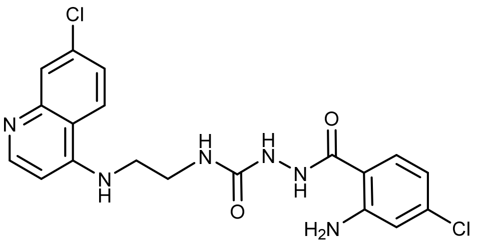
Cl
N
H
N
H
N
H
N
H
O
O
H2N
Cl

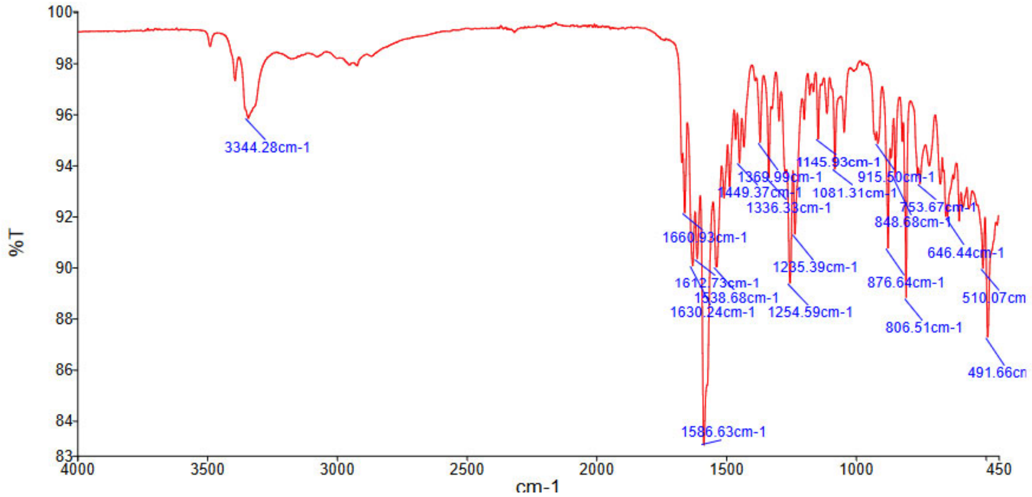
%T
cm-1
3344.28cm-1
1660.93cm-1
1612.73cm-1
1538.68cm-1
1630.24cm-1
1586.63cm-1
1449.37cm-1
1369.99cm-1
1336.33cm-1
1254.59cm-1
1235.39cm-1
1145.93cm-1
1081.31cm-1
915.50cm-1
876.64cm-1
848.68cm-1
806.51cm-1
753.67cm-1
646.44cm-1
510.07cm
491.66cm

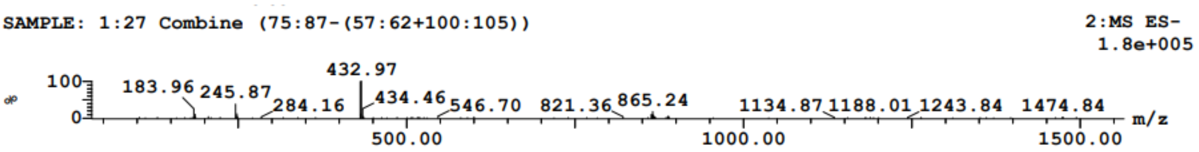
SAMPLE: 1:27 Combine (75:87-(57:62+100:105))
2:MS ES-
1.8e+005
%
432.97
183.96
245.87
284.16
434.46
546.70
821.36
865.24
1134.87
1188.01
1243.84
1474.84
m/z
500.00
1000.00
1500.00

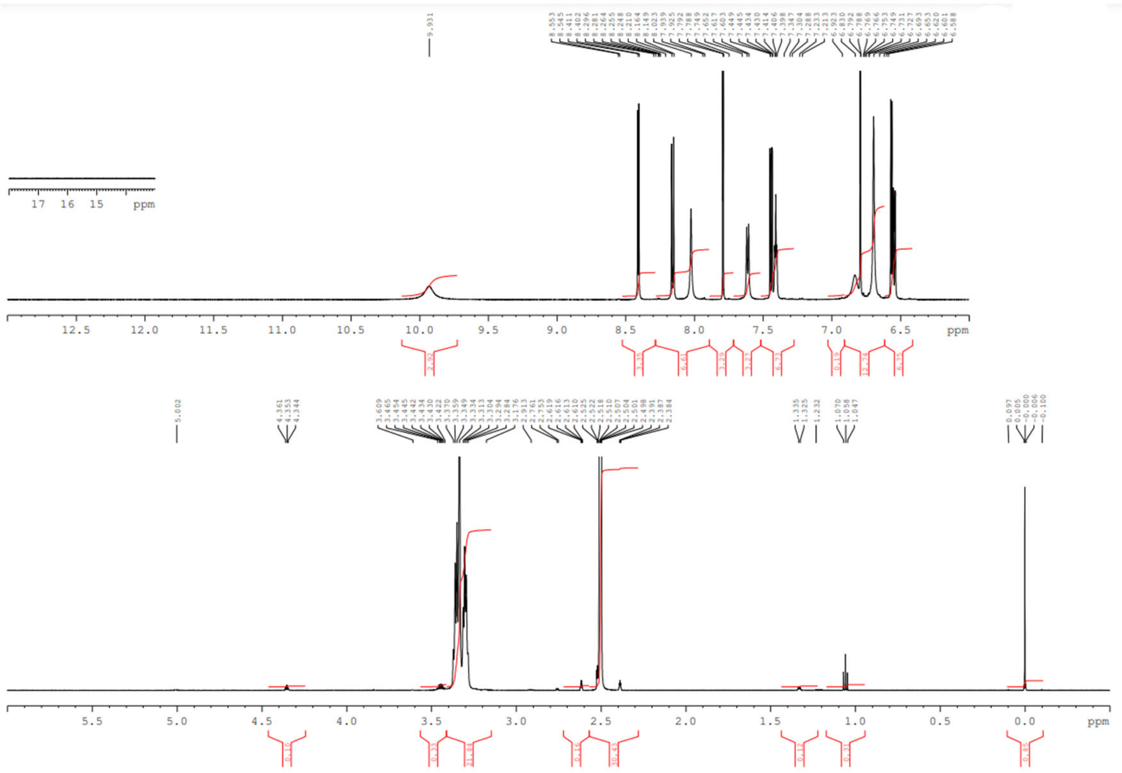

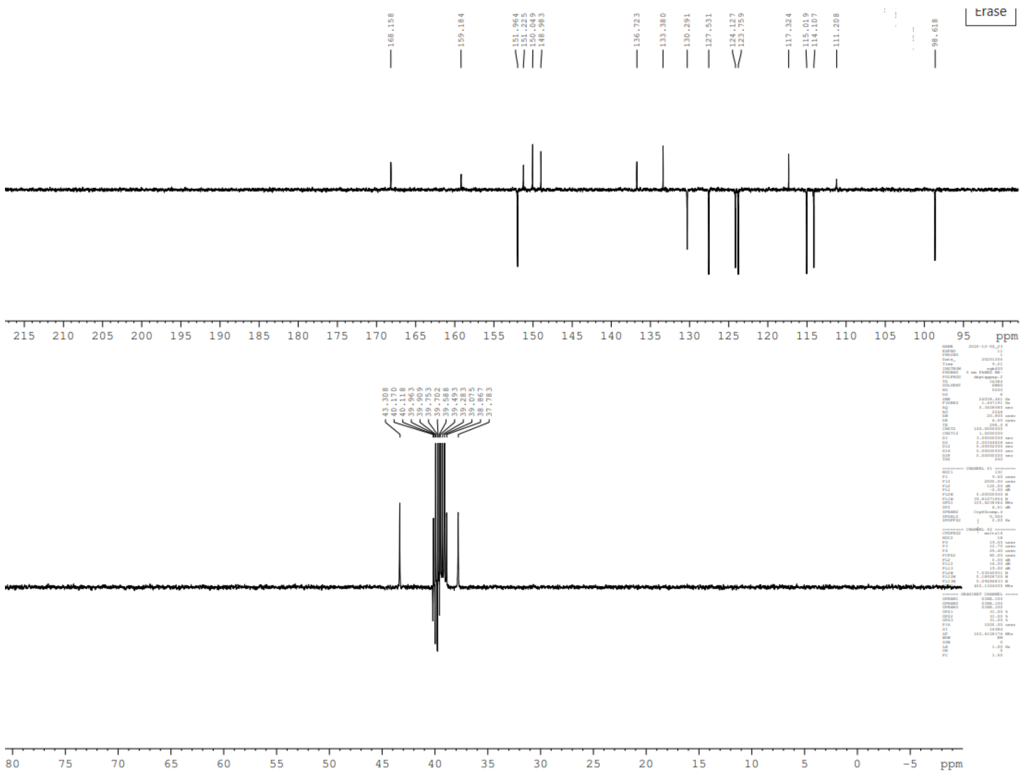

9

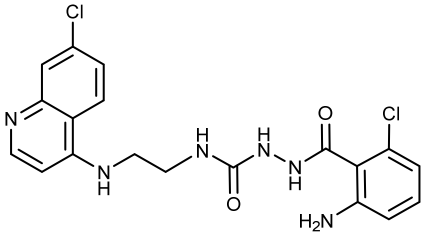

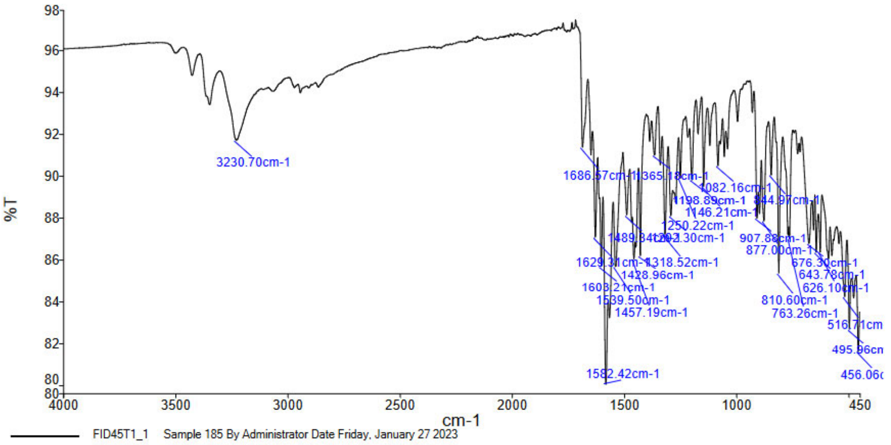

Peak ID	Compound	Time	Mass Found
3		0.69	

SAMPLE: 2:16 Combine (77:89-(59:64+102:107))

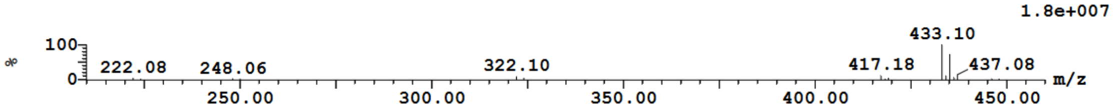

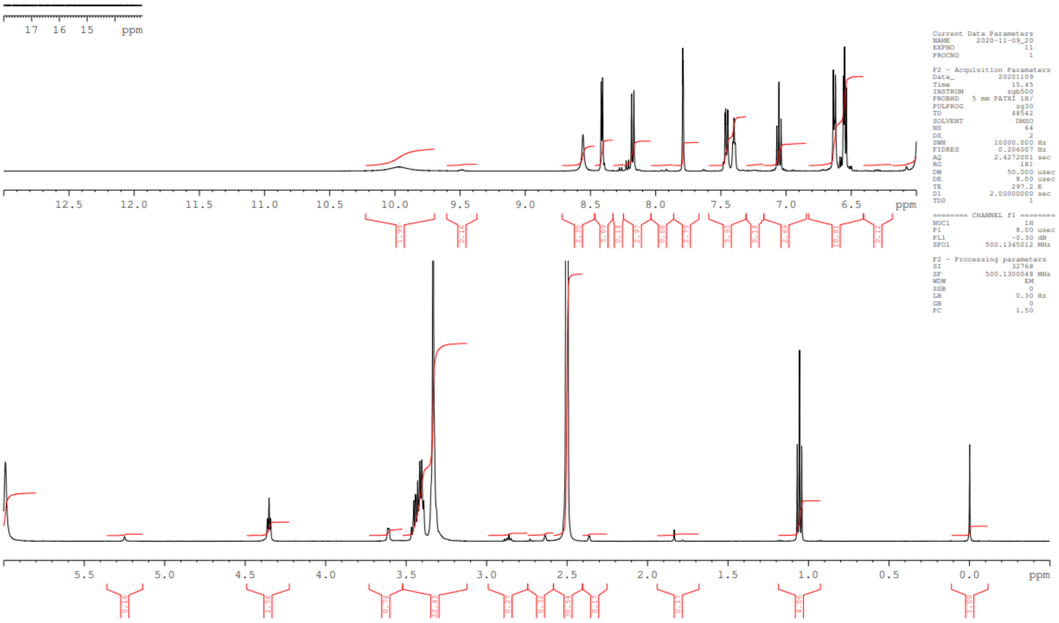
Current Data Parameters
NAME 2020-11-09_20
EXPNO 11
PROCNO 1
F2 - Acquisition Parameters
Date_ 20201109
Time 15.45
INSTRUM zgb500
PROBHD 5 mm PATXI 1H/
PULPROG zg30
TD 48542
SOLVENT DMSO
NS 64
DS 2
SWH 10000.000 Hz
FIDRES 0.206007 Hz
AQ 2.4272001 sec
RG 181
DW 50.000 usec
DE 8.00 usec
TE 297.2 K
D1 2.00000000 sec
TD0 1
======== CHANNEL f1 ========
NUC1 1H
P1 8.00 usec
PL1 -0.30 dB
SFO1 500.1345012 MHz
F2 - Processing parameters
SI 32768
SF 500.1300048 MHz
WDW EM
SSB 0
LB 0.30 Hz
GB 0
PC 1.50
ppm

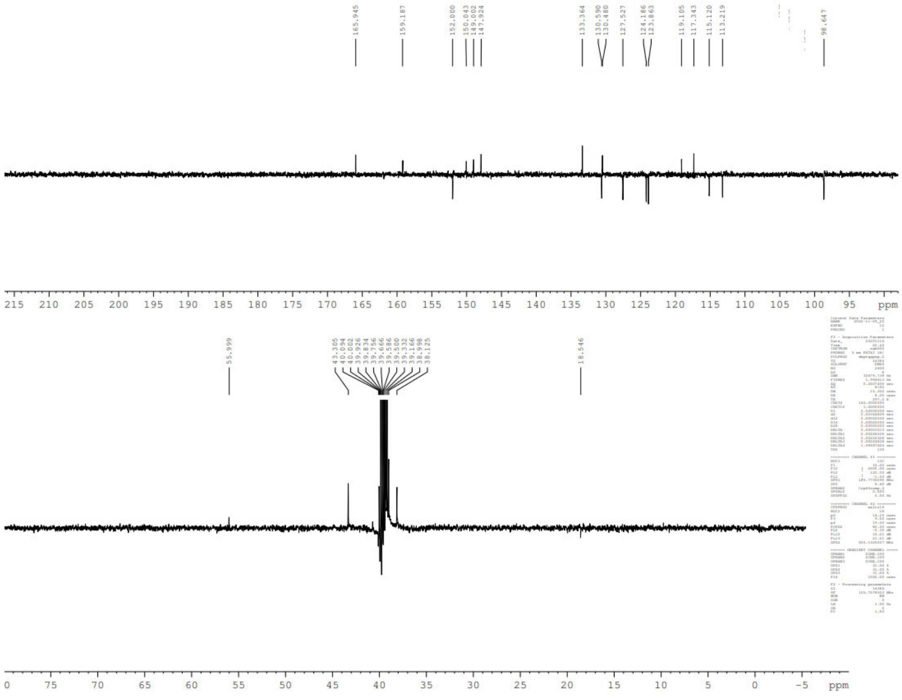
165.945
159.187
152.000
150.043
149.002
147.924
133.364
130.590
130.480
127.527
124.186
123.863
119.105
115.120
113.219
98.647
55.999
18.546
ppm

11

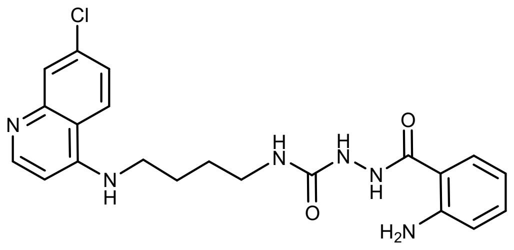
Cl
N
N
H
H
N
H
N
N
H
O
O
H_2N

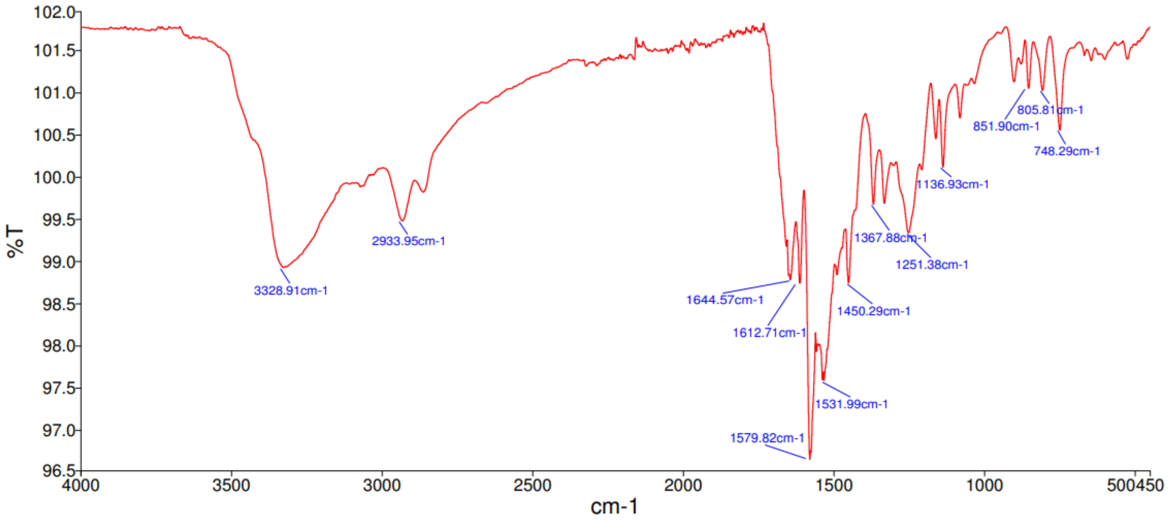
%T
cm-1
3328.91cm-1
2933.95cm-1
1644.57cm-1
1612.71cm-1
1579.82cm-1
1531.99cm-1
1450.29cm-1
1367.88cm-1
1251.38cm-1
1136.93cm-1
851.90cm-1
805.81cm-1
748.29cm-1

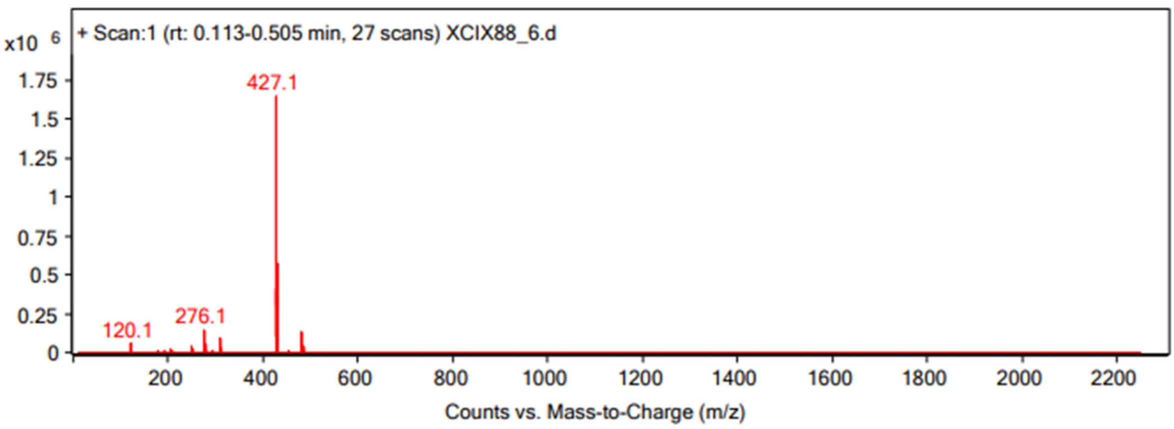
+ Scan:1 (rt: 0.113-0.505 min, 27 scans) XCIX88_6.d
x10 6
427.1
276.1
120.1
Counts vs. Mass-to-Charge (m/z)

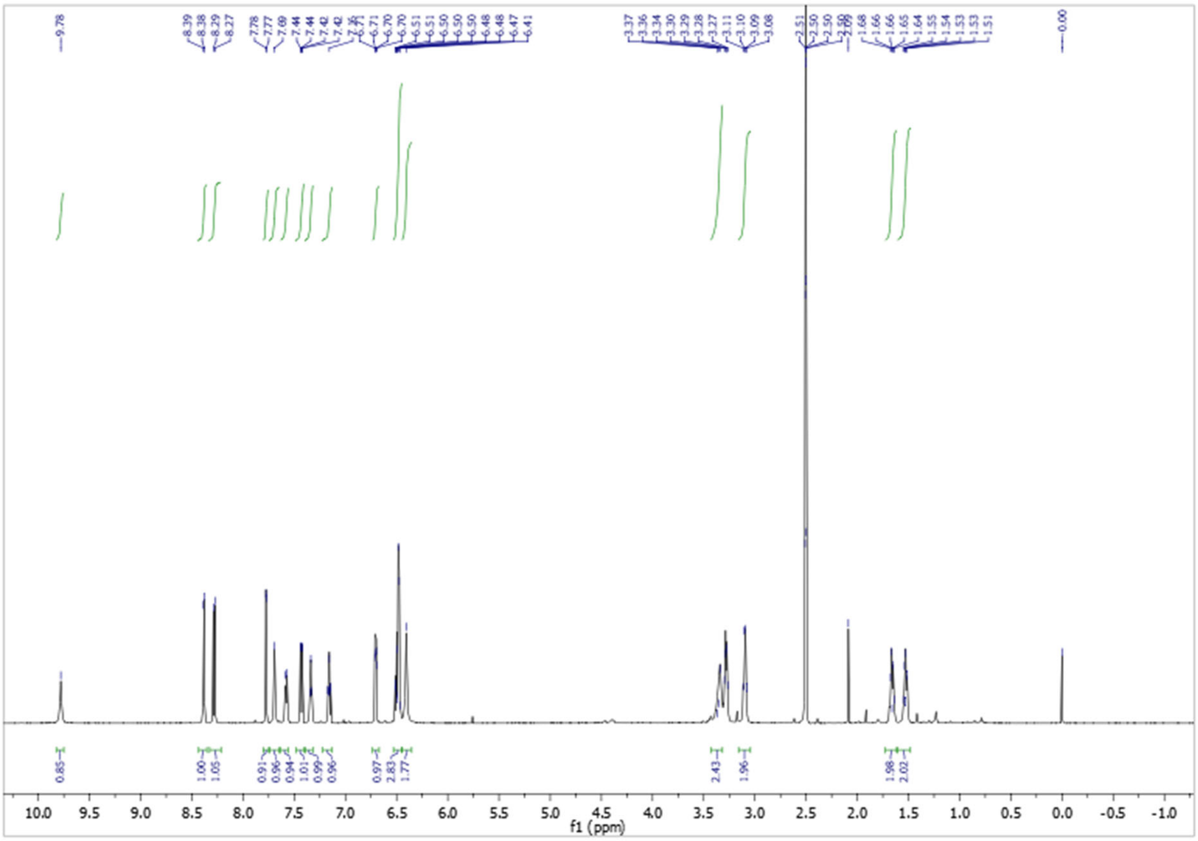
9.78
8.39 8.38 8.29 8.27
7.78 7.77 7.69 7.44 7.44 7.42 7.42 6.71 6.71 6.70 6.70 6.51 6.51 6.50 6.50 6.50 6.48 6.48 6.47 6.41
3.37 3.36 3.34 3.30 3.29 3.28 3.27 3.11 3.10 3.09 3.08
2.51 2.50 2.50 2.50 2.09
1.68 1.66 1.66 1.65 1.64 1.55 1.54 1.53 1.53 1.51
0.00
0.85 1.00 1.05 0.91 0.96 0.94 1.01 0.99 0.96 0.97 2.83 1.77 2.43 1.96 1.98 2.02
10.0 9.5 9.0 8.5 8.0 7.5 7.0 6.5 6.0 5.5 5.0 4.5 4.0 3.5 3.0 2.5 2.0 1.5 1.0 0.5 0.0 -0.5 -1.0
f1 (ppm)

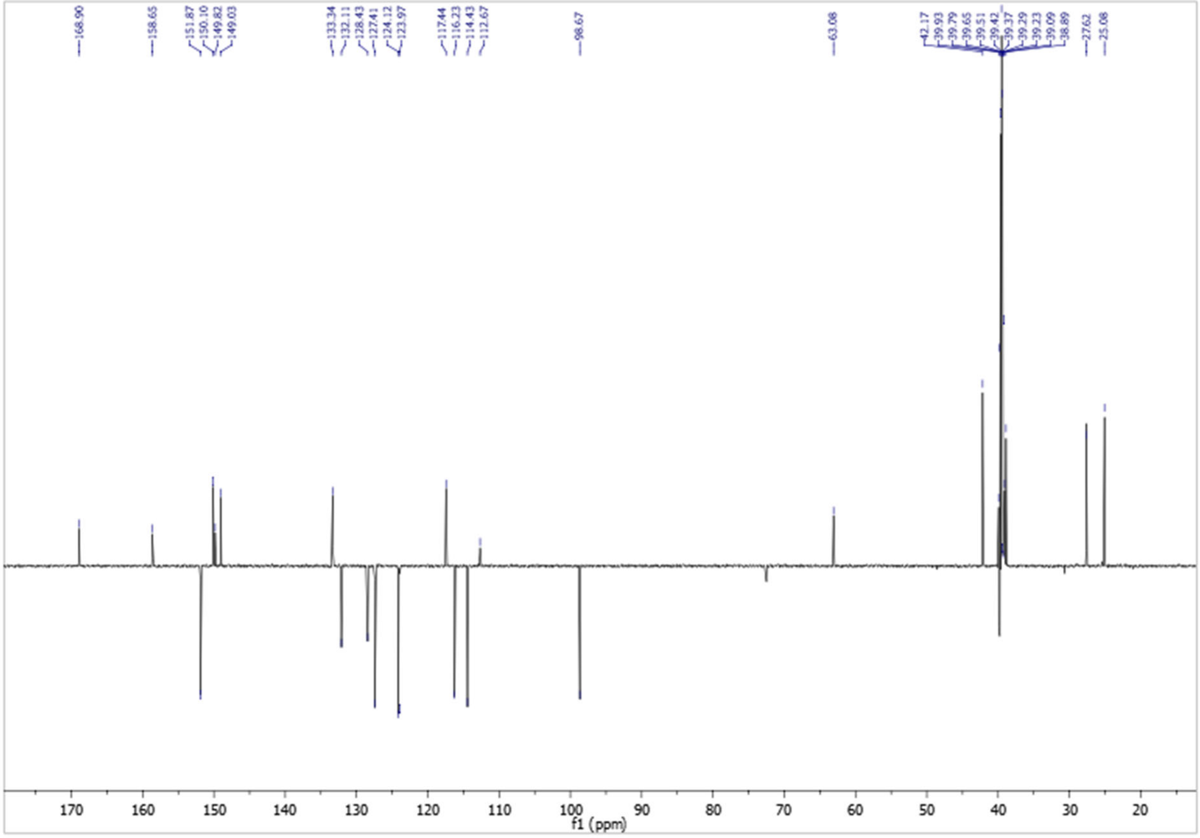
168.90
158.65
151.87 150.10 149.82 149.03
133.34 132.11 128.43 127.41 124.12 123.97
117.44 116.23 114.43 112.67
98.67
63.08
42.17 39.93 39.79 39.65 39.51 39.42 39.37 39.29 39.23 39.09 38.89
27.62 25.08
170 160 150 140 130 120 110 100 90 80 70 60 50 40 30 20
f1 (ppm)

12

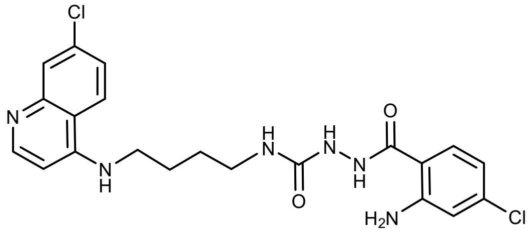

Cl
N
H
O
H₂N
Cl

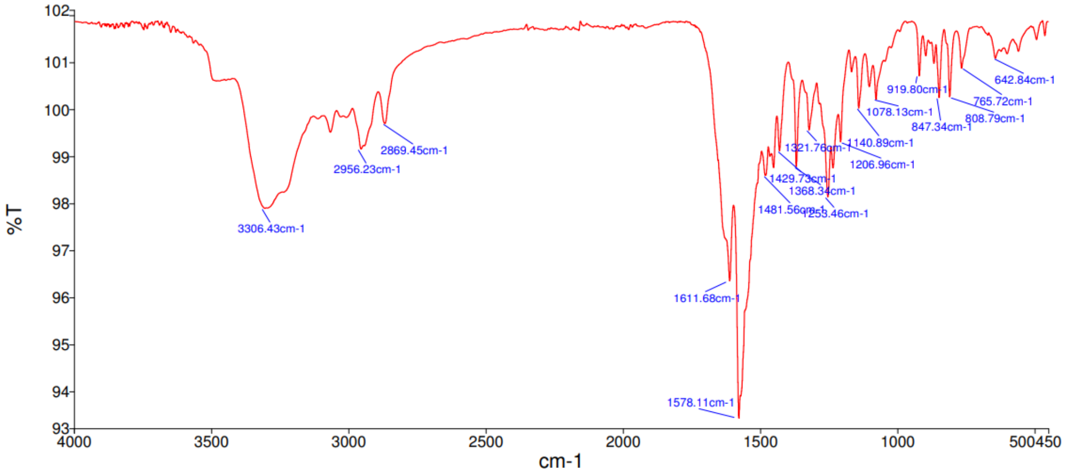

%T
cm-1
3306.43cm-1
2956.23cm-1
2869.45cm-1
1611.68cm-1
1578.11cm-1
1481.56cm-1
1429.73cm-1
1368.34cm-1
1321.76cm-1
1253.46cm-1
1206.96cm-1
1140.89cm-1
1078.13cm-1
919.80cm-1
847.34cm-1
808.79cm-1
765.72cm-1
642.84cm-1

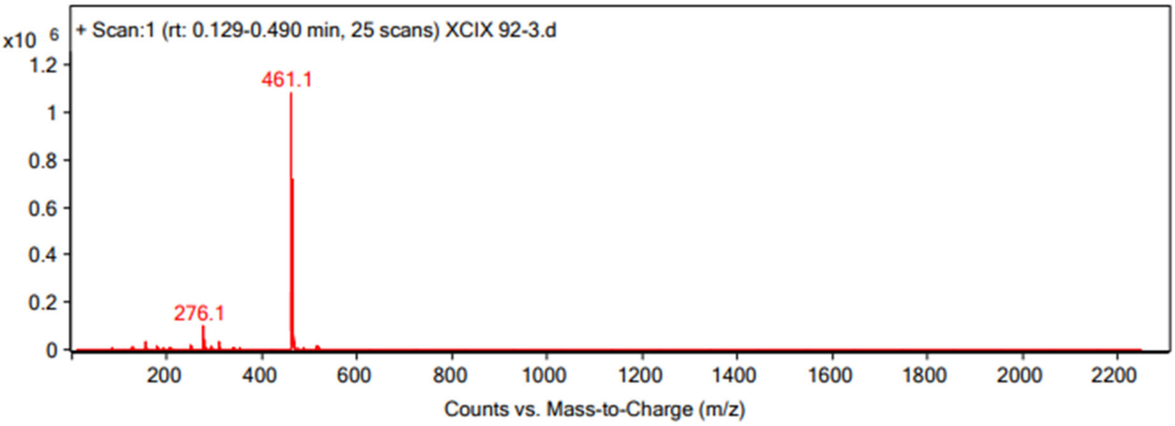

x10 6
+ Scan:1 (rt: 0.129-0.490 min, 25 scans) XCIX 92-3.d
461.1
276.1
Counts vs. Mass-to-Charge (m/z)

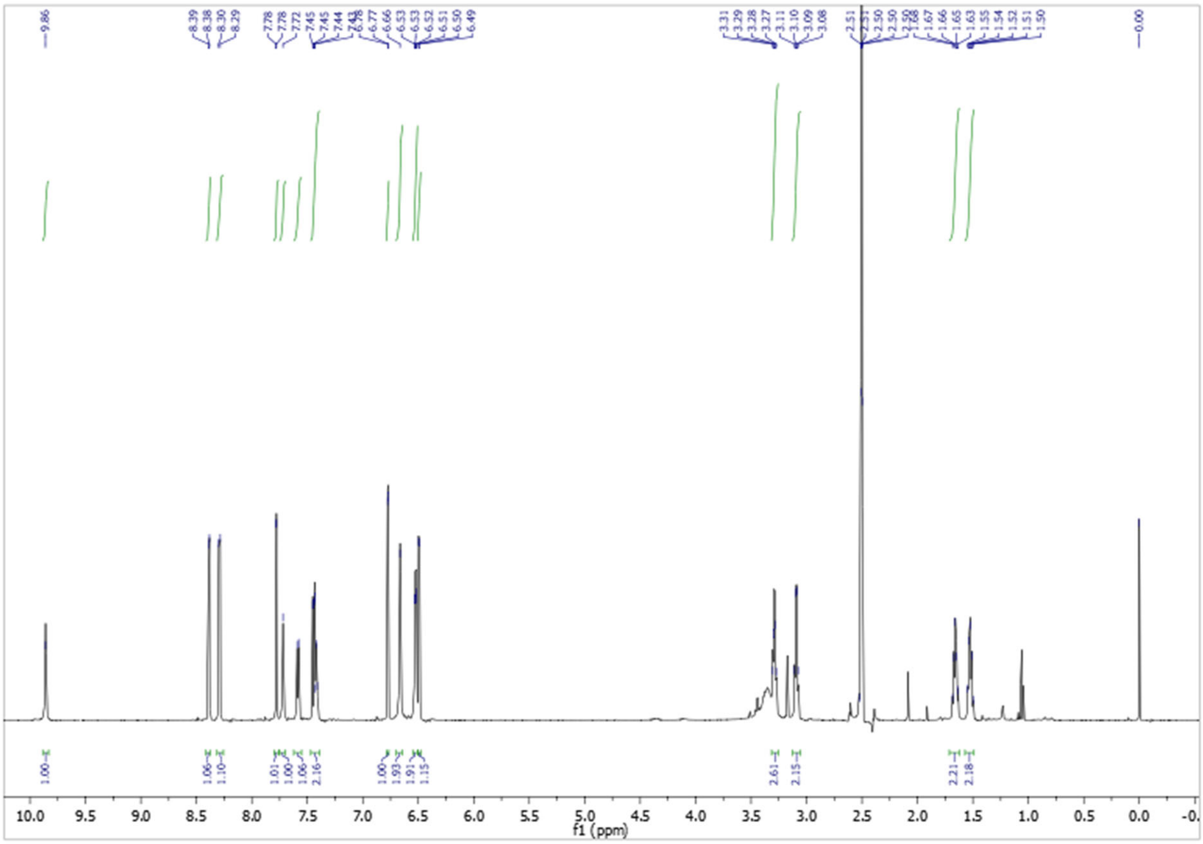
9.86
8.39
8.38
8.30
8.29
7.78
7.78
7.72
7.45
7.45
7.44
6.78
6.77
6.66
6.53
6.53
6.52
6.51
6.50
6.49
3.31
3.29
3.28
3.27
3.11
3.10
3.09
3.08
2.51
2.51
2.50
2.50
2.50
1.68
1.67
1.66
1.65
1.63
1.55
1.54
1.52
1.51
1.50
0.00
1.00
1.06
1.10
1.01
1.00
1.06
2.16
1.00
1.93
1.91
1.15
2.61
2.15
2.21
2.18
f1 (ppm)

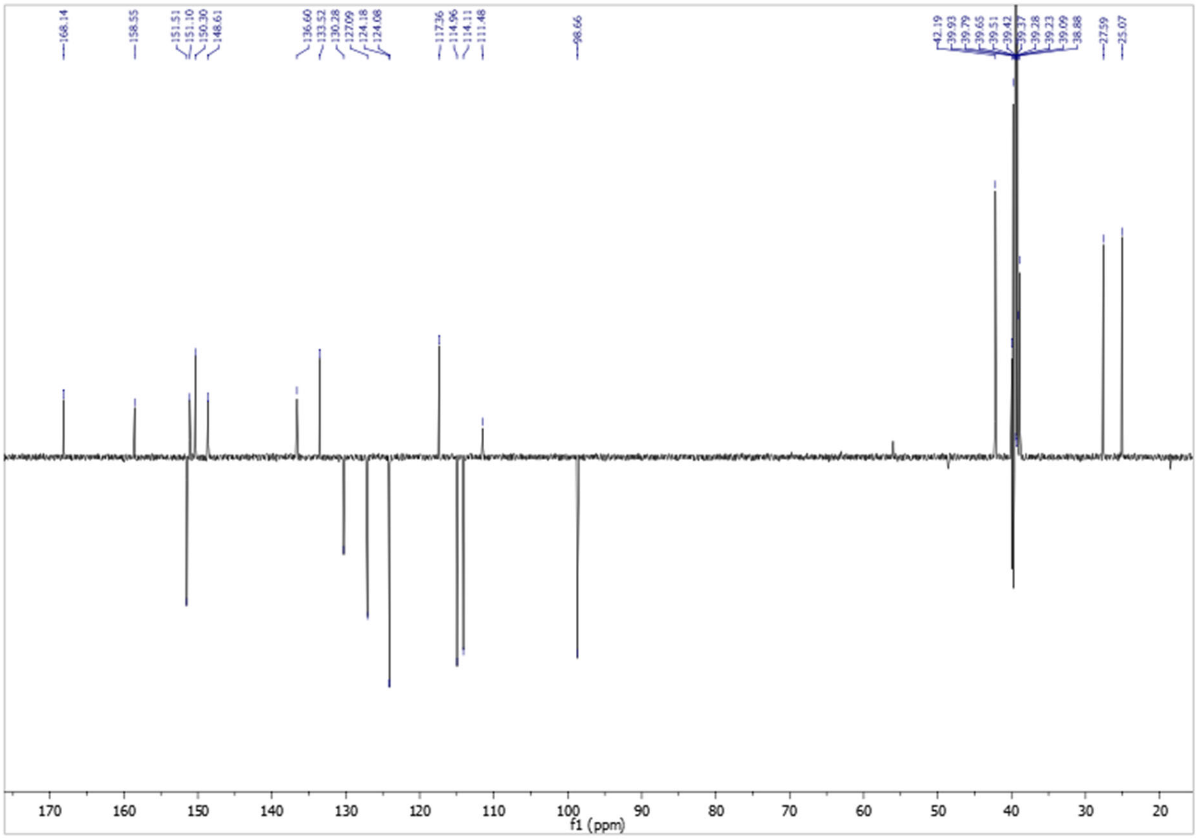
168.14
158.55
151.51
151.10
150.30
148.61
136.60
133.52
130.28
127.09
124.18
124.08
117.36
114.96
114.11
111.48
98.66
42.19
39.93
39.79
39.65
39.51
39.42
39.37
39.28
39.23
39.09
38.88
27.59
25.07
f1 (ppm)

14

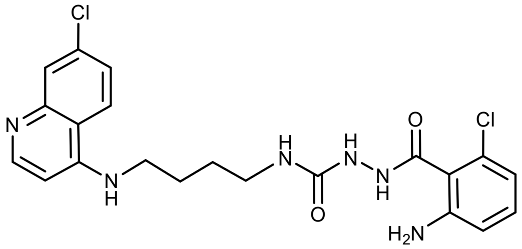
Cl
N
H
N
H
N
H
N
H
O
O
Cl
H2N

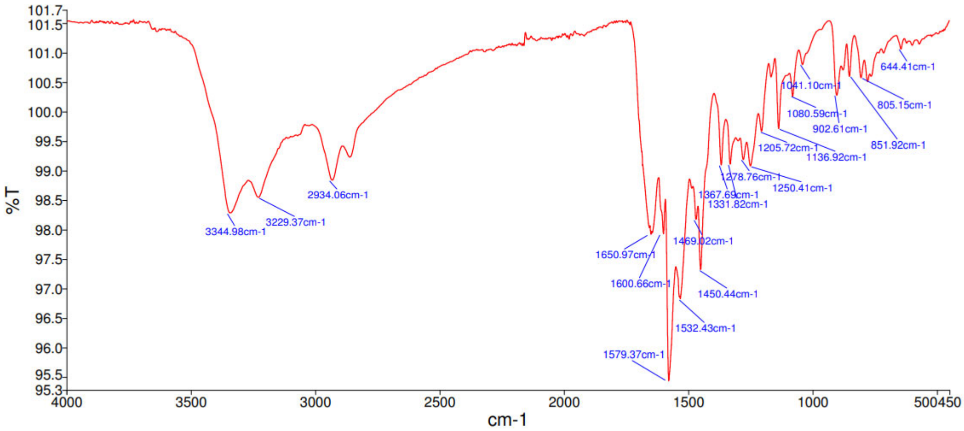
%T
cm-1
3344.98cm-1
3229.37cm-1
2934.06cm-1
1650.97cm-1
1600.66cm-1
1579.37cm-1
1532.43cm-1
1469.02cm-1
1450.44cm-1
1367.69cm-1
1331.82cm-1
1278.76cm-1
1250.41cm-1
1205.72cm-1
1136.92cm-1
1080.59cm-1
1041.10cm-1
902.61cm-1
851.92cm-1
805.15cm-1
644.41cm-1

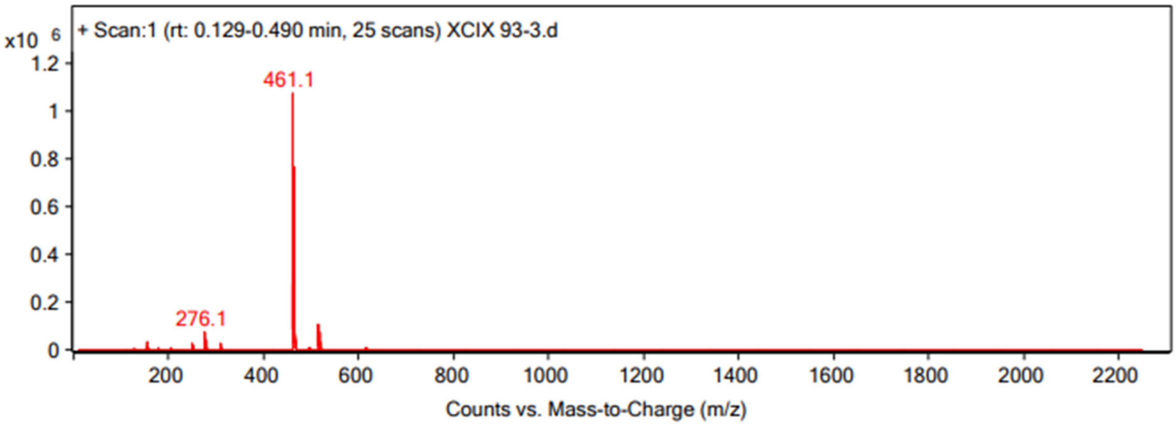
x10 6
+ Scan:1 (rt: 0.129-0.490 min, 25 scans) XCIX 93-3.d
461.1
276.1
Counts vs. Mass-to-Charge (m/z)

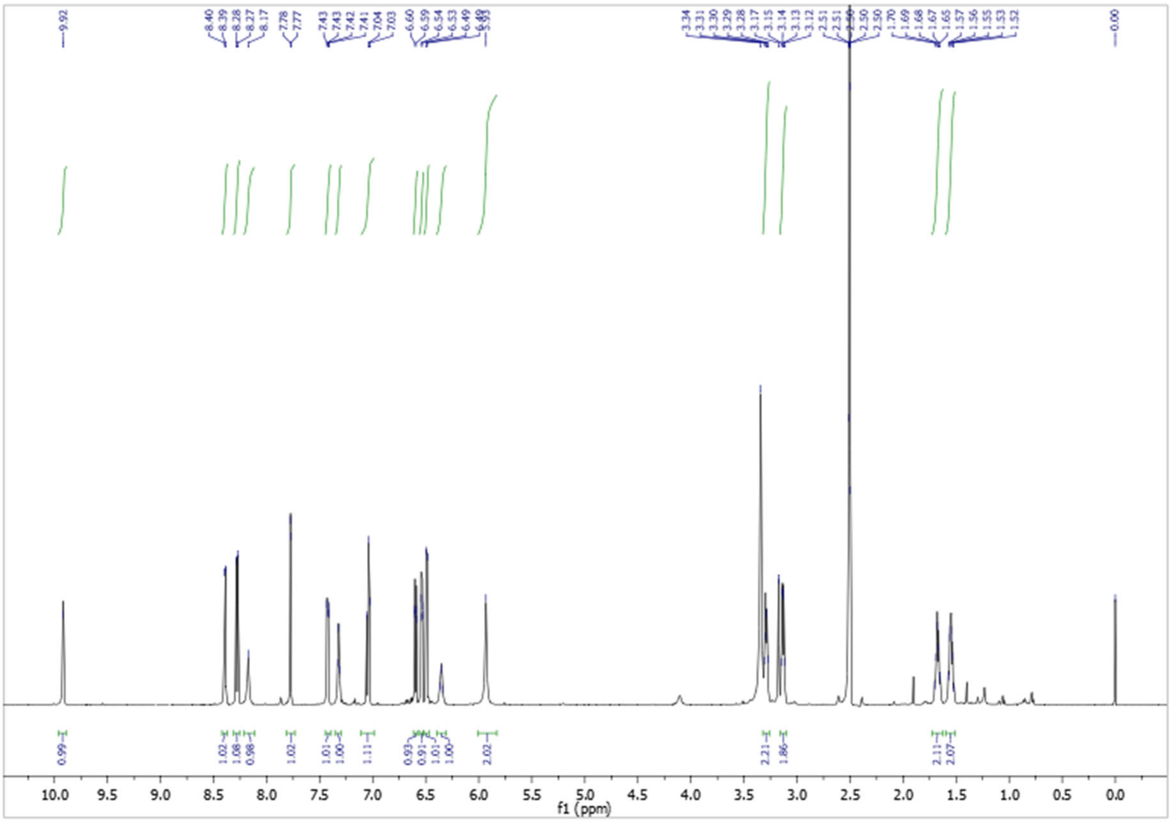
9.92
8.40
8.39
8.28
8.27
8.17
7.78
7.77
7.43
7.43
7.42
7.41
7.04
7.03
6.60
6.59
6.54
6.53
6.49
6.48
5.93
3.34
3.31
3.30
3.29
3.28
3.17
3.15
3.14
3.13
3.12
2.51
2.51
2.50
2.50
2.50
1.70
1.69
1.68
1.67
1.65
1.57
1.56
1.55
1.53
1.52
0.00
0.99
1.02
1.08
0.98
1.02
1.01
1.00
1.11
0.93
0.91
1.01
1.00
2.02
2.21
1.86
2.11
2.07
10.0
9.5
9.0
8.5
8.0
7.5
7.0
6.5
6.0
5.5
5.0
4.5
4.0
3.5
3.0
2.5
2.0
1.5
1.0
0.5
0.0
f1 (ppm)

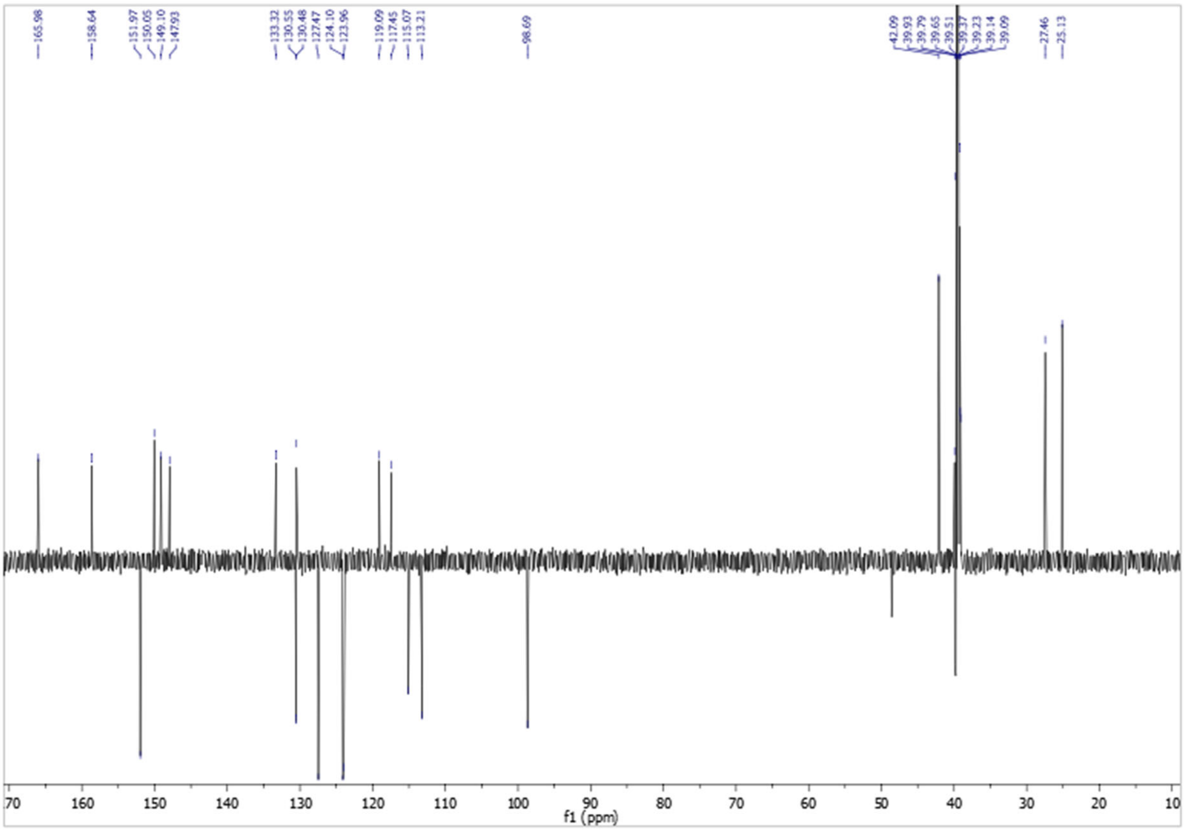
165.98
158.64
151.97
150.05
149.10
147.93
133.32
130.55
130.48
127.47
124.10
123.96
119.09
117.45
115.07
113.21
98.69
42.09
39.93
39.79
39.65
39.51
39.37
39.23
39.14
39.09
27.46
25.13
70
160
150
140
130
120
110
100
90
80
70
60
50
40
30
20
10
f1 (ppm)

15

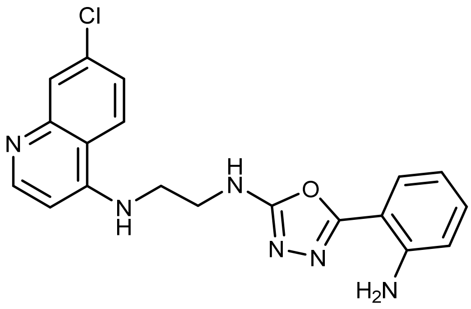

Cl
N
H
N
N
H
O
N
N
H2N

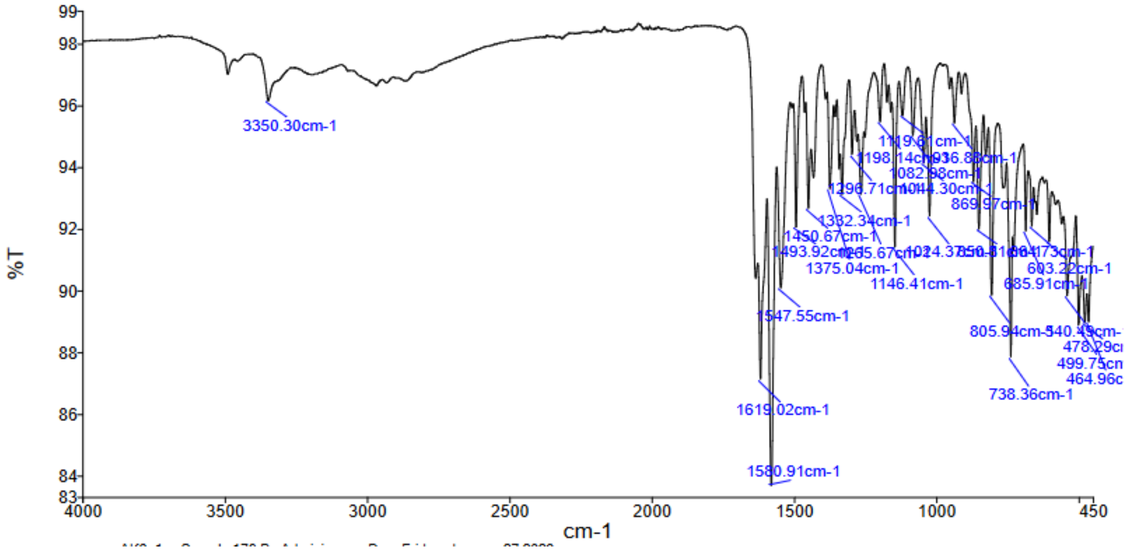

%T
cm-1
99
98
96
94
92
90
88
86
84
83
4000
3500
3000
2500
2000
1500
1000
450
3350.30cm-1
1619.02cm-1
1580.91cm-1
1547.55cm-1
1493.92cm-1
1450.67cm-1
1375.04cm-1
1332.34cm-1
1146.41cm-1
1082.98cm-1
869.97cm-1
805.94cm-1
738.36cm-1
685.91cm-1
603.22cm-1

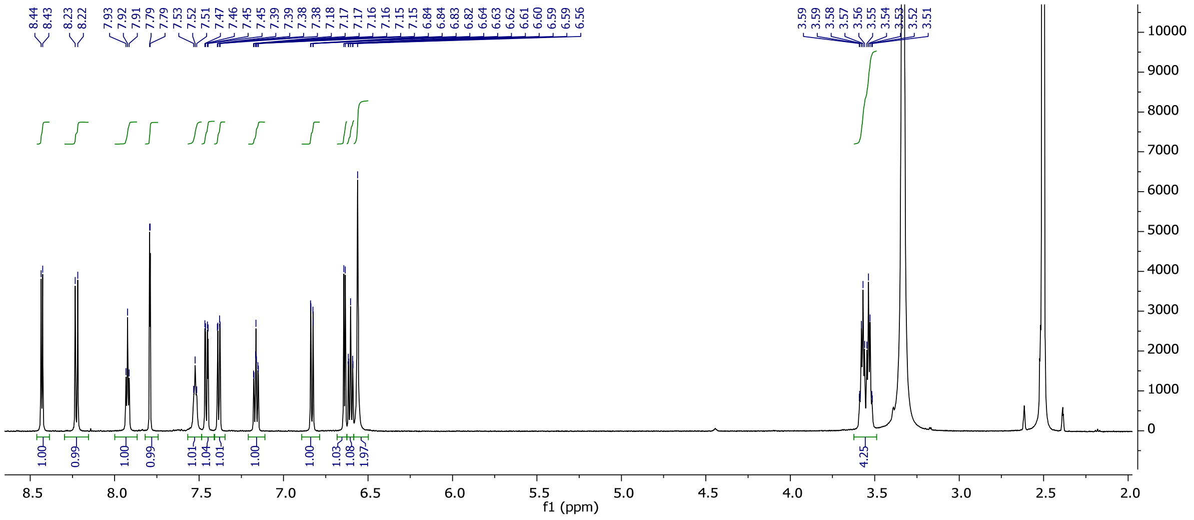

8.44
8.43
8.23
8.22
7.93
7.92
7.91
7.79
7.79
7.53
7.52
7.51
7.47
7.46
7.45
7.45
7.39
7.39
7.38
7.38
7.18
7.17
7.17
7.16
7.15
7.15
6.84
6.84
6.83
6.82
6.64
6.63
6.62
6.61
6.60
6.59
6.59
6.56
3.59
3.59
3.58
3.57
3.56
3.55
3.54
3.53
3.52
3.51
1.00
0.99
1.00
0.99
1.01
1.04
1.01
1.00
1.00
1.03
1.08
1.97
4.25
f1 (ppm)

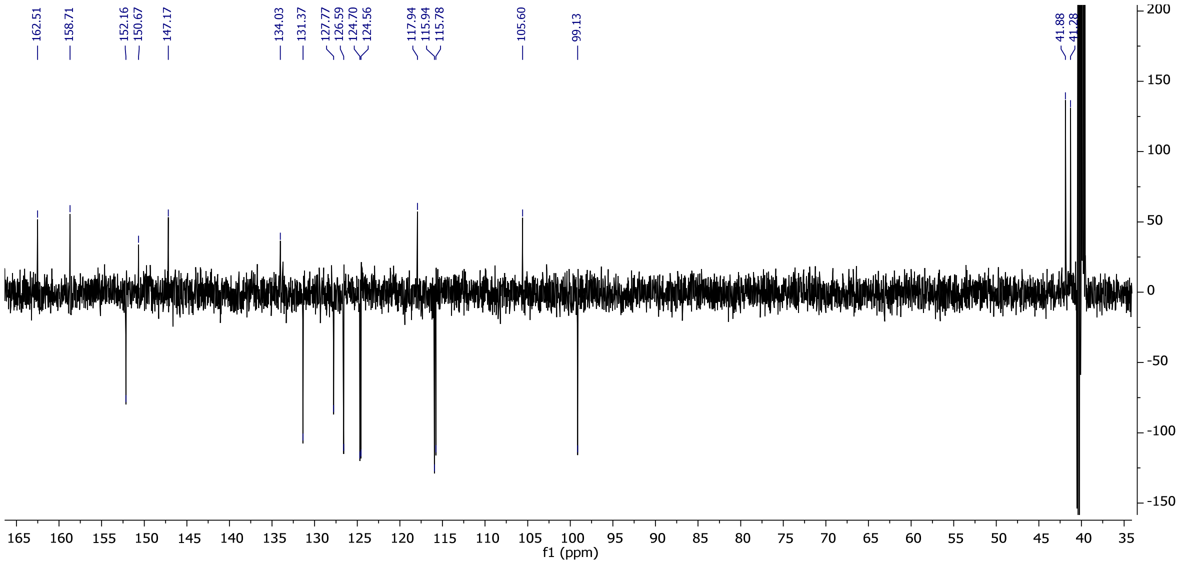
162.51
158.71
152.16
150.67
147.17
134.03
131.37
127.77
126.59
124.70
124.56
117.94
115.94
115.78
105.60
99.13
41.88
41.28
f1 (ppm)

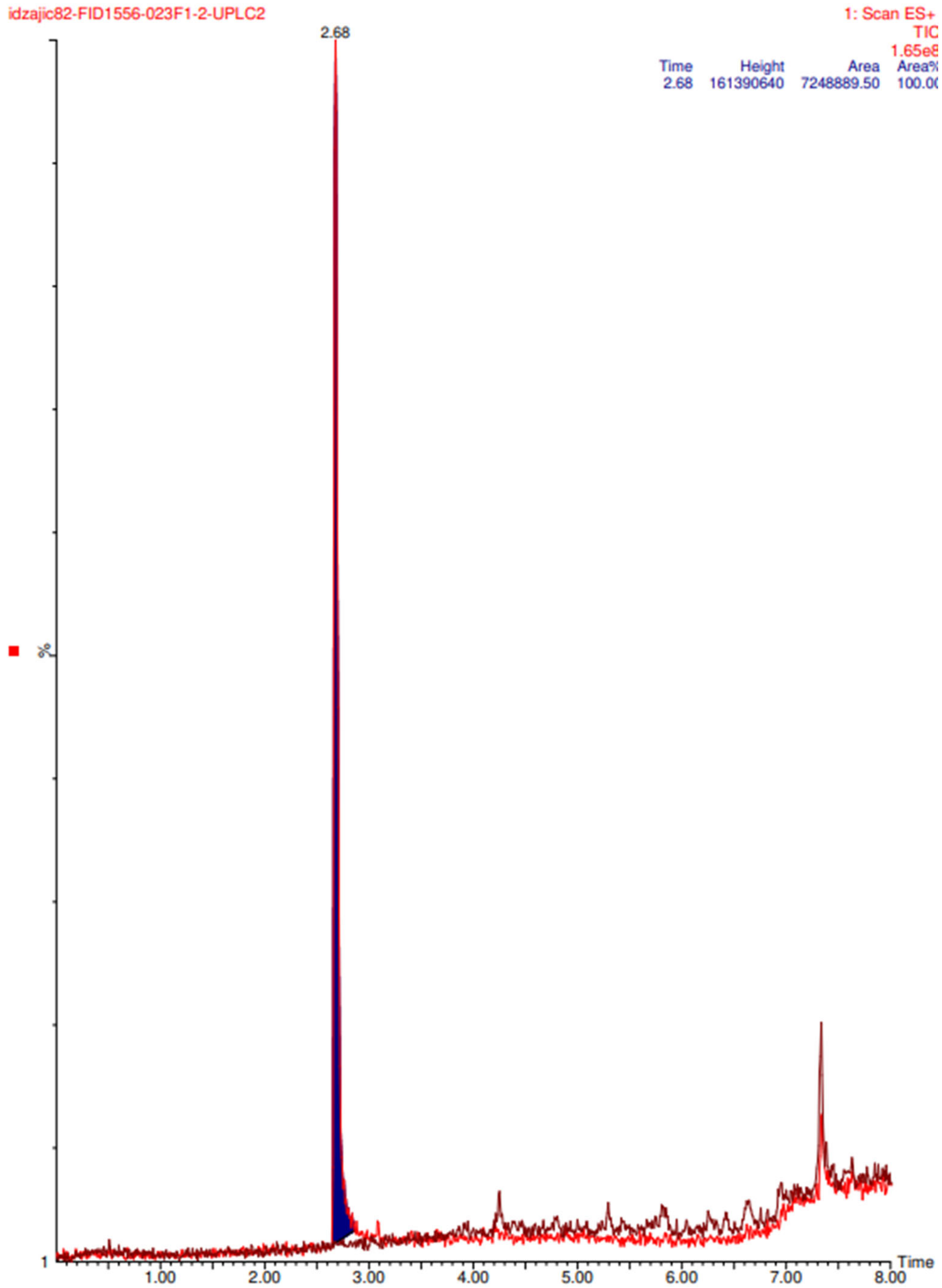

Peak ID	Compound	Time	Mass Found
3		2.68	

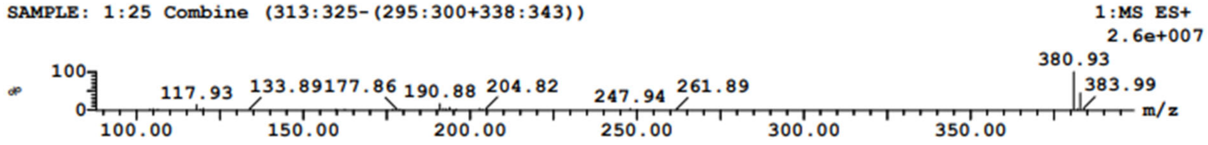

Peak ID	Compound	Time	Mass Found
3		2.68	

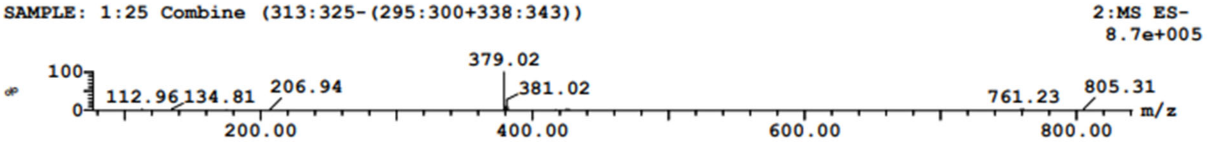

16

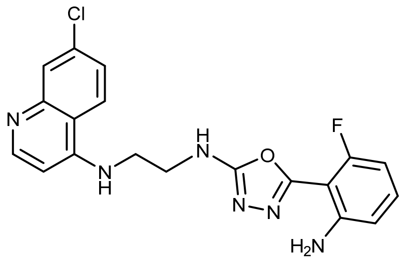

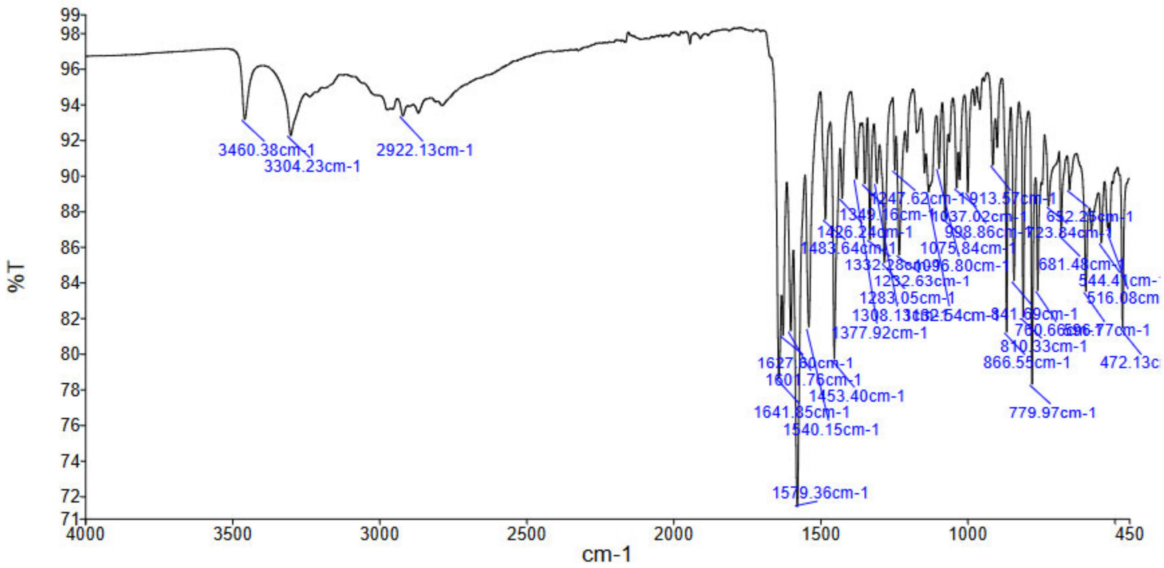

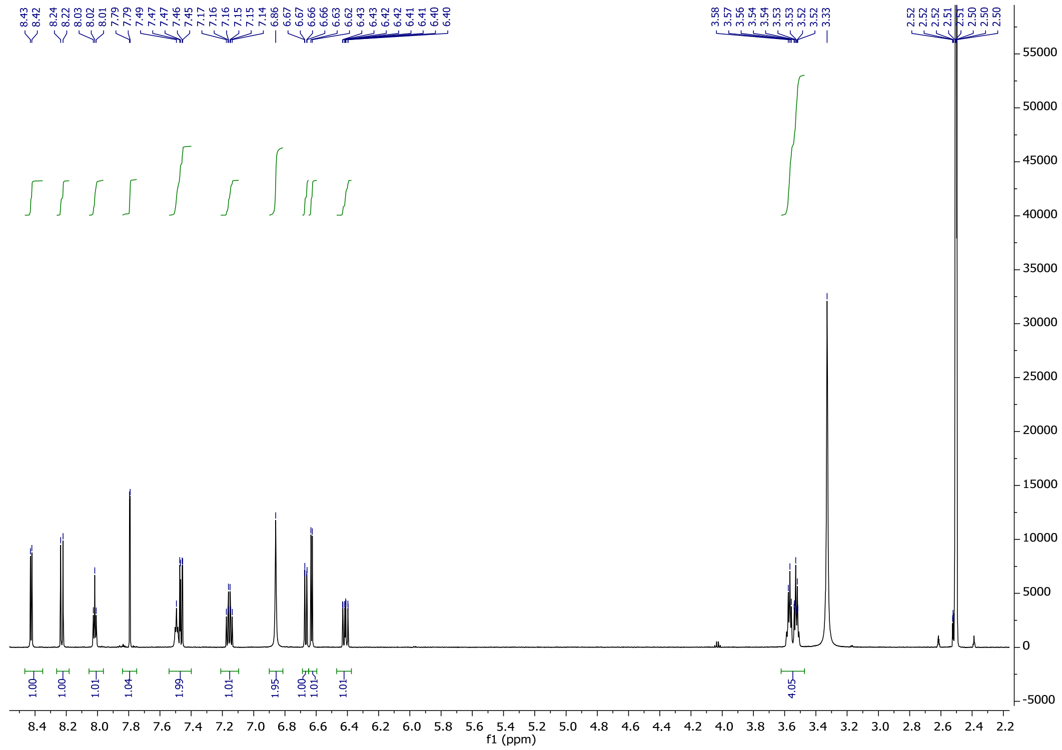

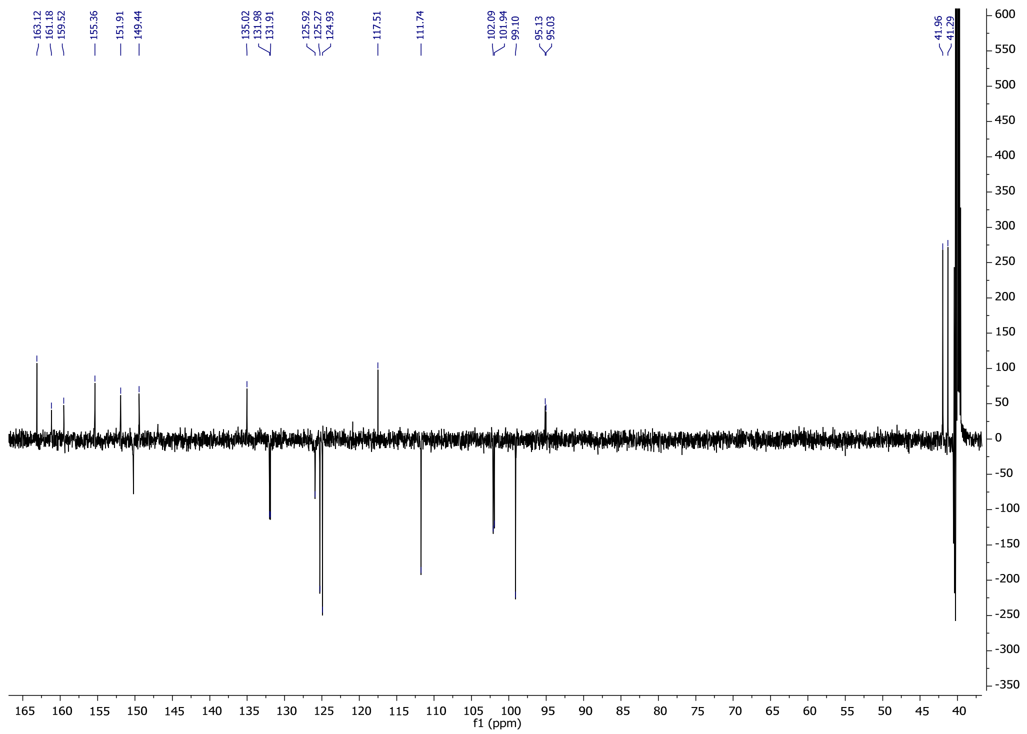

163.12
161.18
159.52
155.36
151.91
149.44
135.02
131.98
131.91
125.92
125.27
124.93
117.51
111.74
102.09
101.94
99.10
95.13
95.03
41.96
41.29
f1 (ppm)

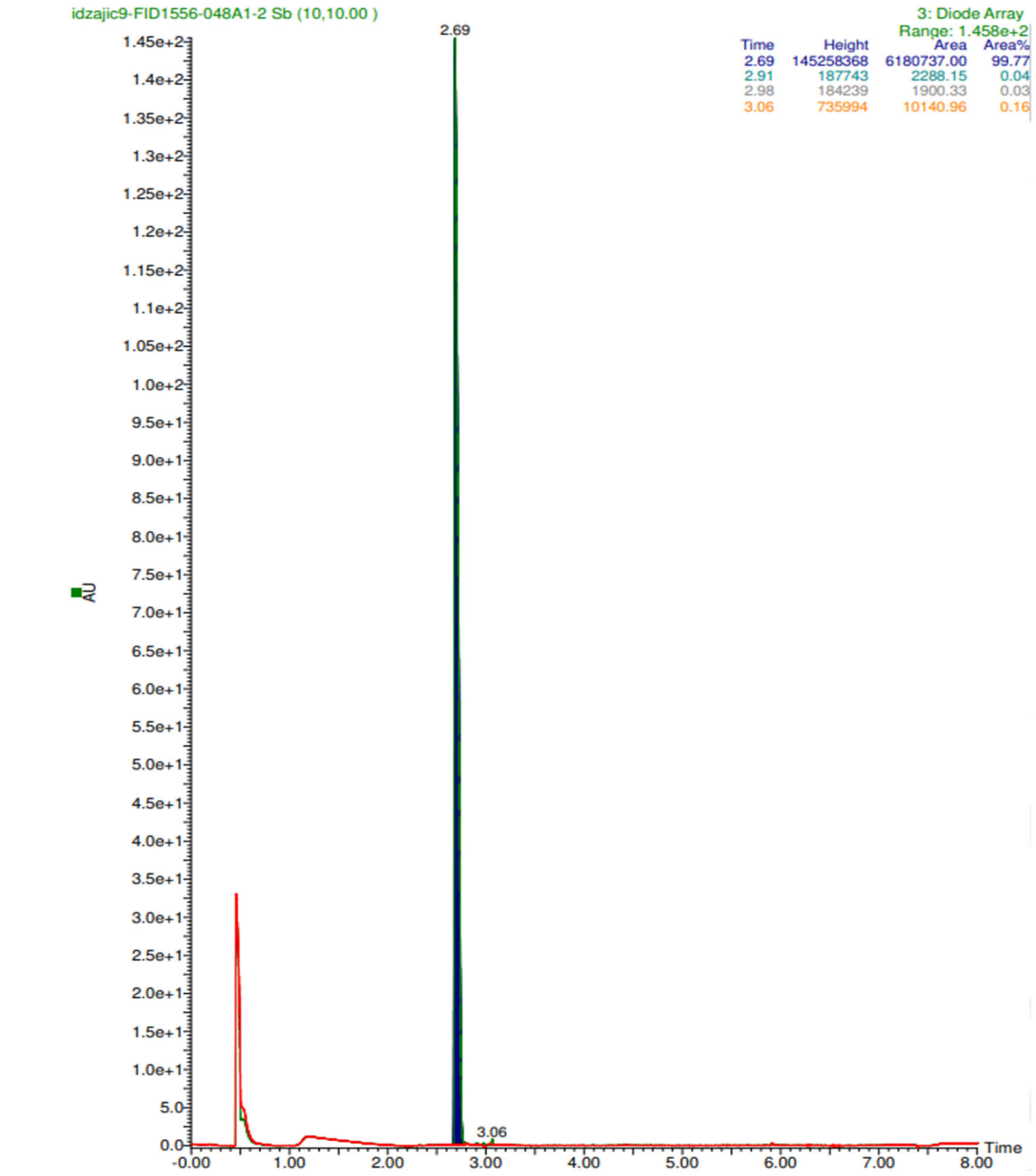

Peak ID	Compound	Time	Mass Found
3		2.71	

SAMPLE: 2:20 Combine (317:329-(299:304+342:347))

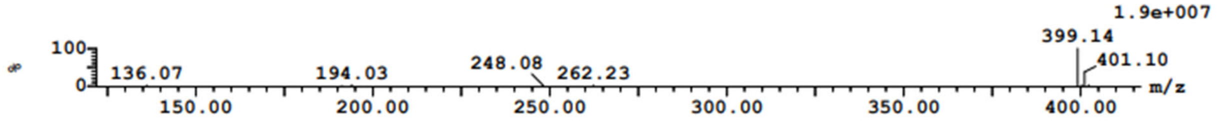

Peak ID	Compound	Time	Mass Found
3		2.71	

SAMPLE: 2:20 Combine (317:329-(299:304+342:347))

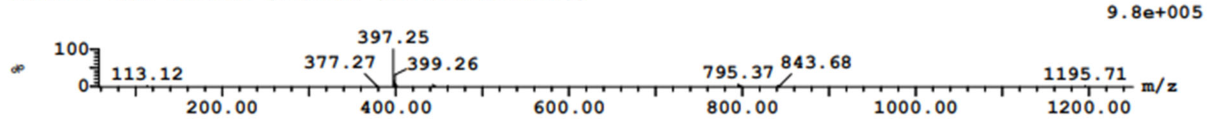

17

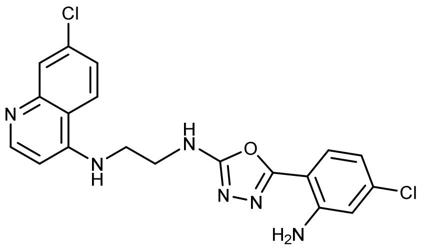
Cl
N
N
H
H
N
O
N
N
Cl
H2N

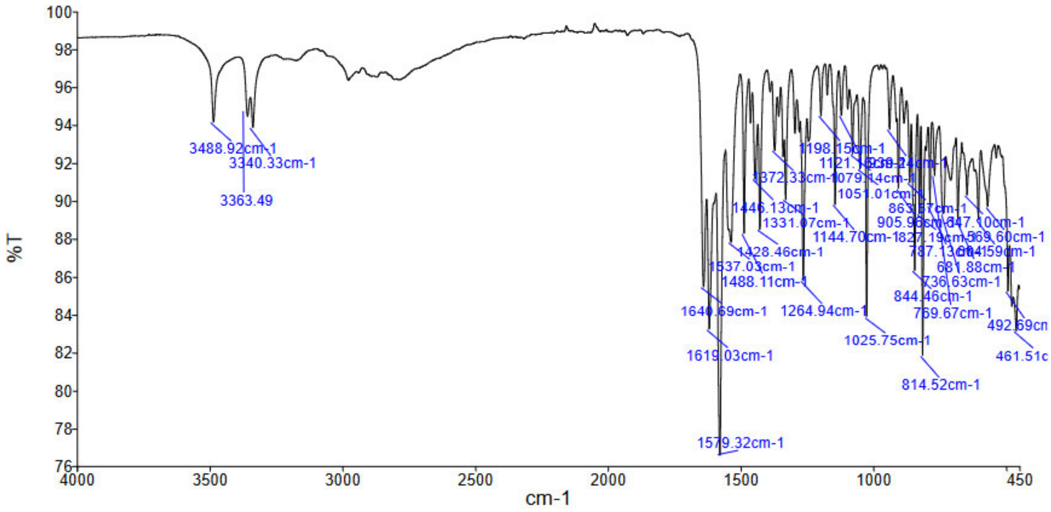
%T
cm-1
3488.92cm-1
3340.33cm-1
3363.49
1579.32cm-1
1619.03cm-1
1264.94cm-1
1025.75cm-1
814.52cm-1

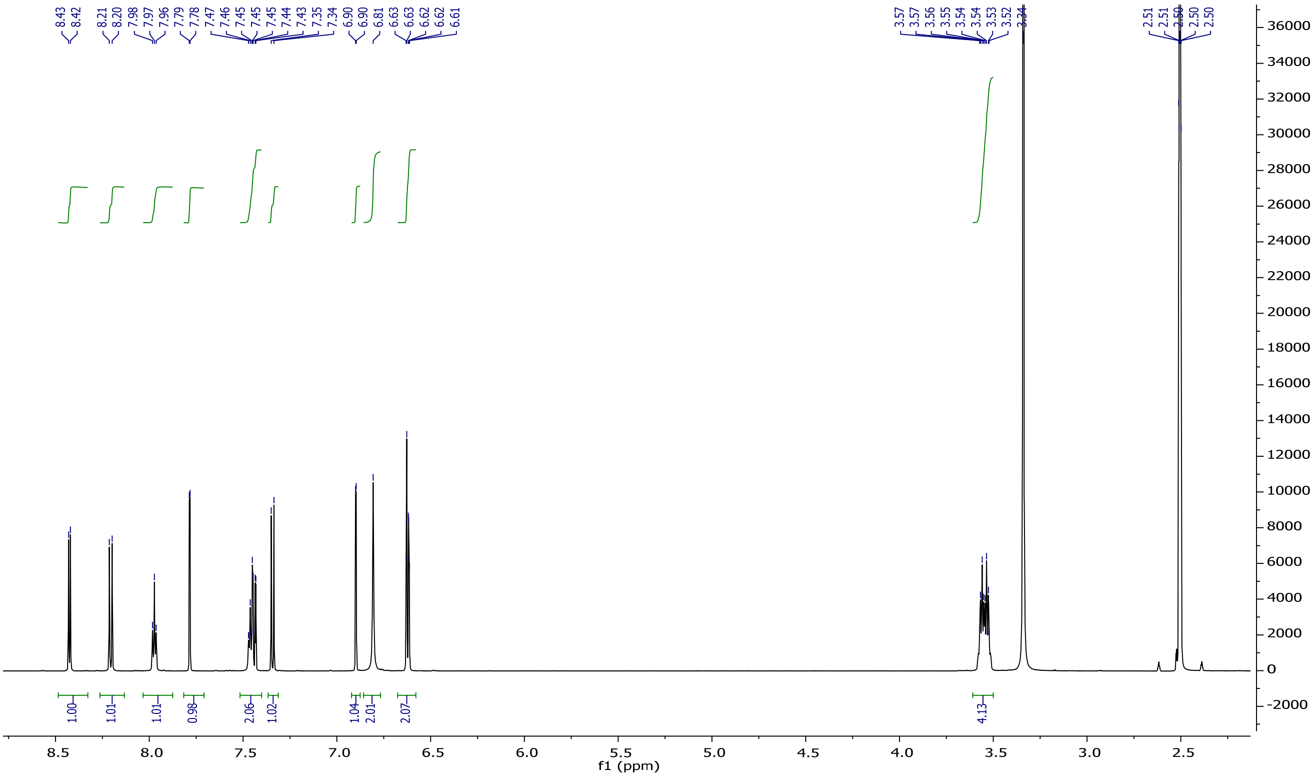
f1 (ppm)

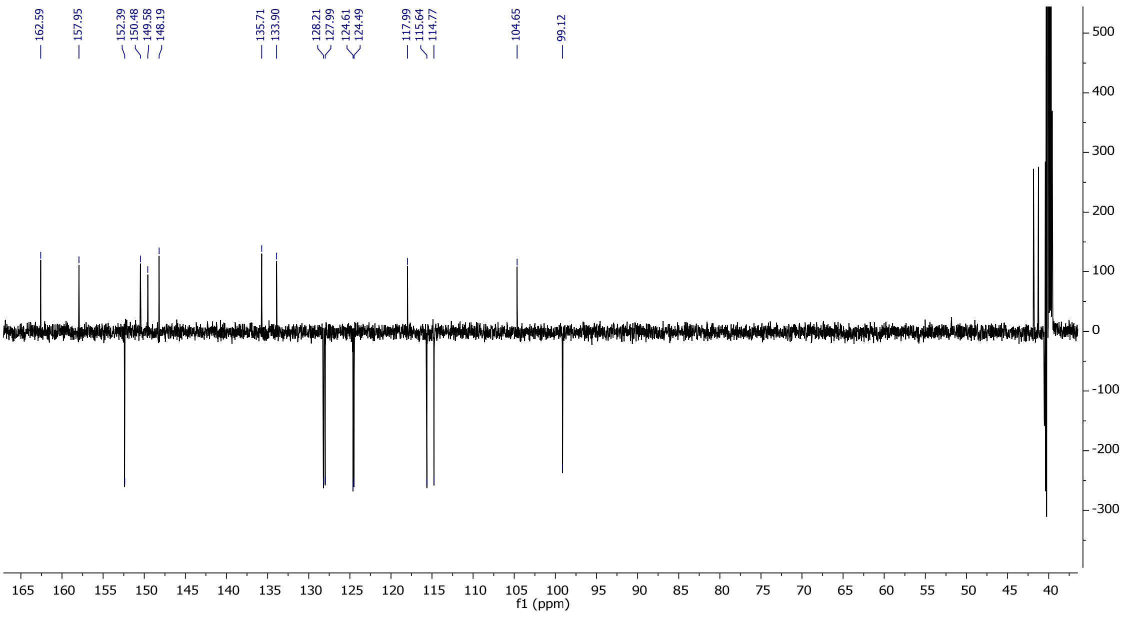

162.59
157.95
152.39
150.48
149.58
148.19
135.71
133.90
128.21
127.99
124.61
124.49
117.99
115.64
114.77
104.65
99.12
500
400
300
200
100
0
-100
-200
-300
165 160 155 150 145 140 135 130 125 120 115 110 105 100 95 90 85 80 75 70 65 60 55 50 45 40
f1 (ppm)

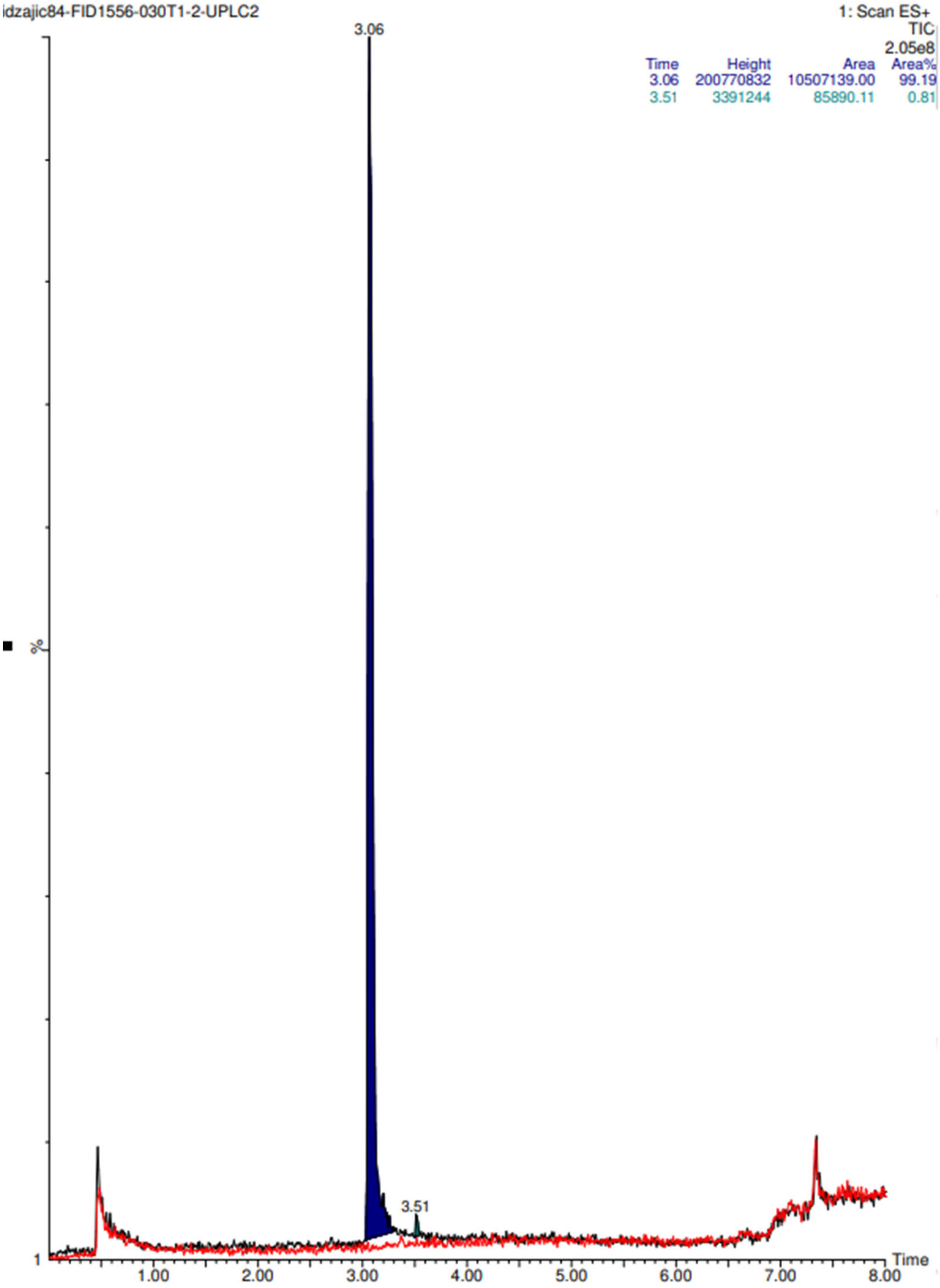

Peak ID	Compound	Time	Mass Found
3		3.07	

SAMPLE: 1:9 Combine (360:372-(342:347+385:390))

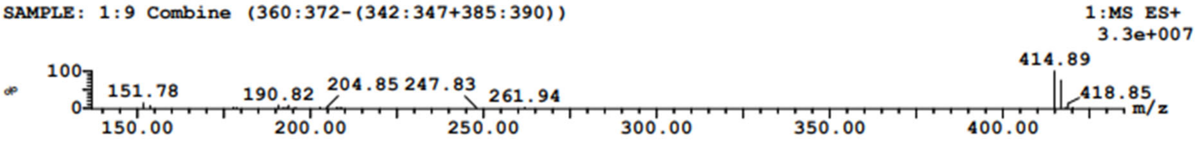

Peak ID	Compound	Time	Mass Found
3		3.07	

SAMPLE: 1:9 Combine (359:371-(341:346+384:389))

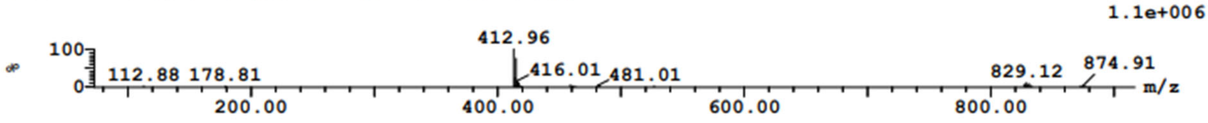

18

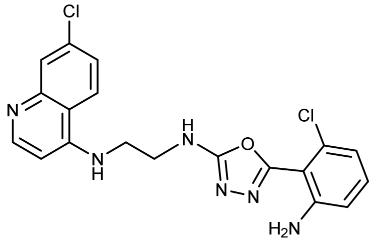
Cl
N
H
N
H
N
O
N
N
Cl
H2N

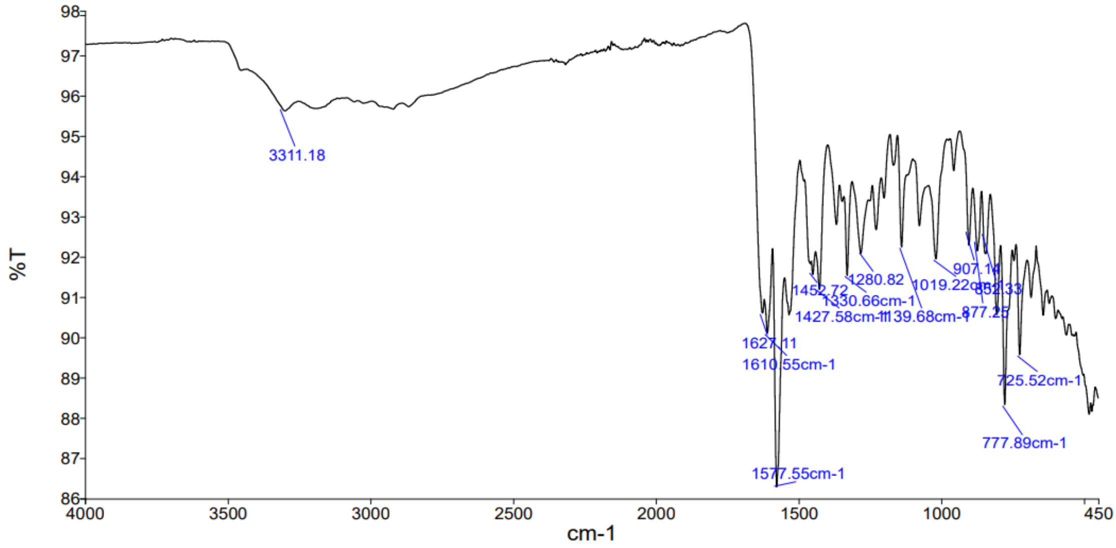
%T
cm-1
3311.18
1627.11
1610.55cm-1
1577.55cm-1
1452.72
1427.58cm-1
1330.66cm-1
1280.82
1139.68cm-1
1019.22cm-1
907.14
877.25
777.89cm-1
725.52cm-1

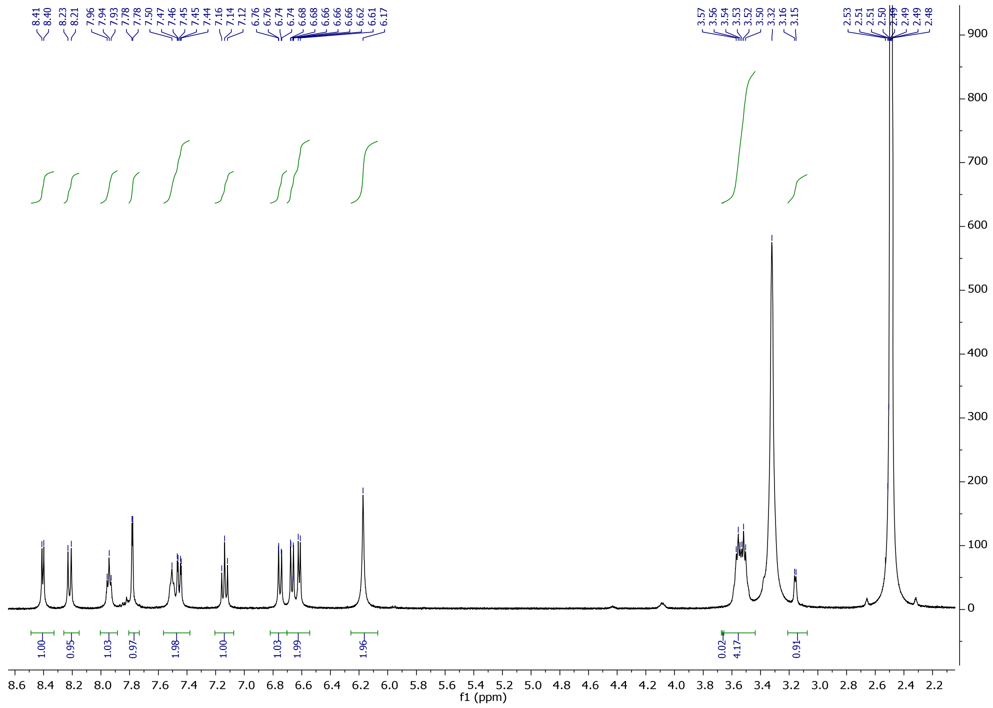
f1 (ppm)

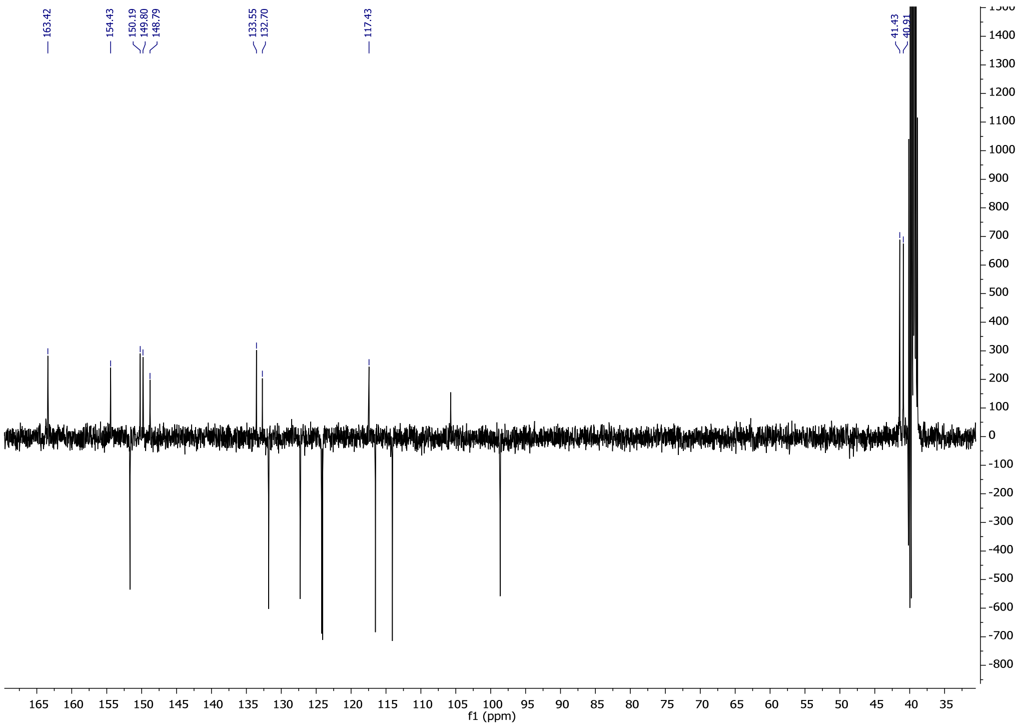

163.42
154.43
150.19
149.80
148.79
133.55
132.70
117.43
41.43
40.91
165 160 155 150 145 140 135 130 125 120 115 110 105 100 95 90 85 80 75 70 65 60 55 50 45 40 35
f1 (ppm)
1400
1300
1200
1100
1000
900
800
700
600
500
400
300
200
100
0
-100
-200
-300
-400
-500
-600
-700
-800

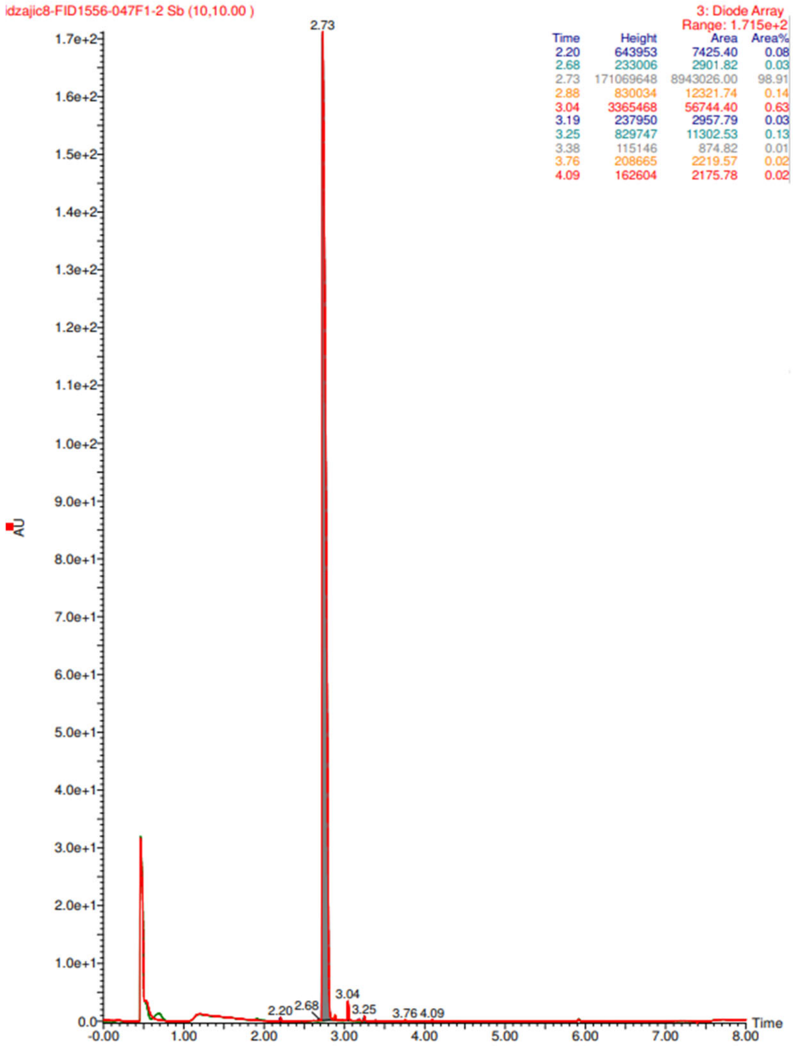

Peak ID	Compound	Time	Mass Found
3		2.75	

SAMPLE: 2:16 Combine (322:334-(304:309+347:352))

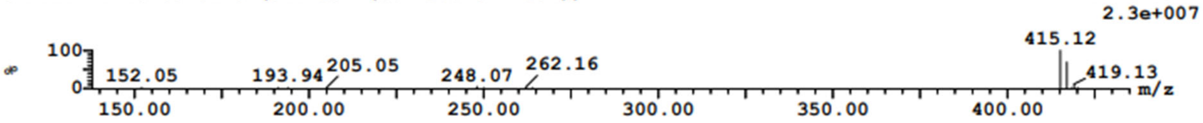

Peak ID	Compound	Time	Mass Found
3		2.75	

SAMPLE: 2:16 Combine (324:336-(306:311+349:354))

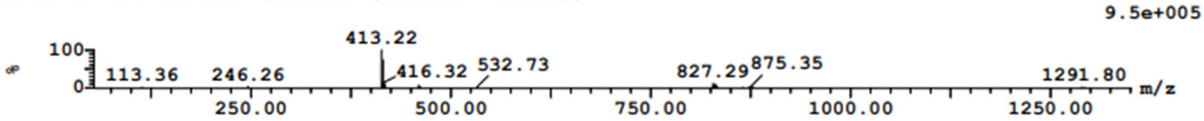

19

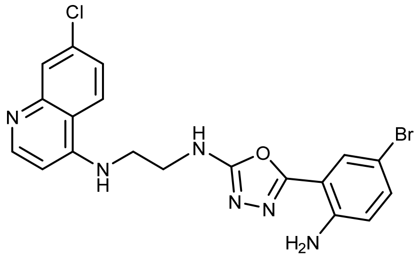
Cl
N
N
H
H
N
O
N
N
Br
H2N

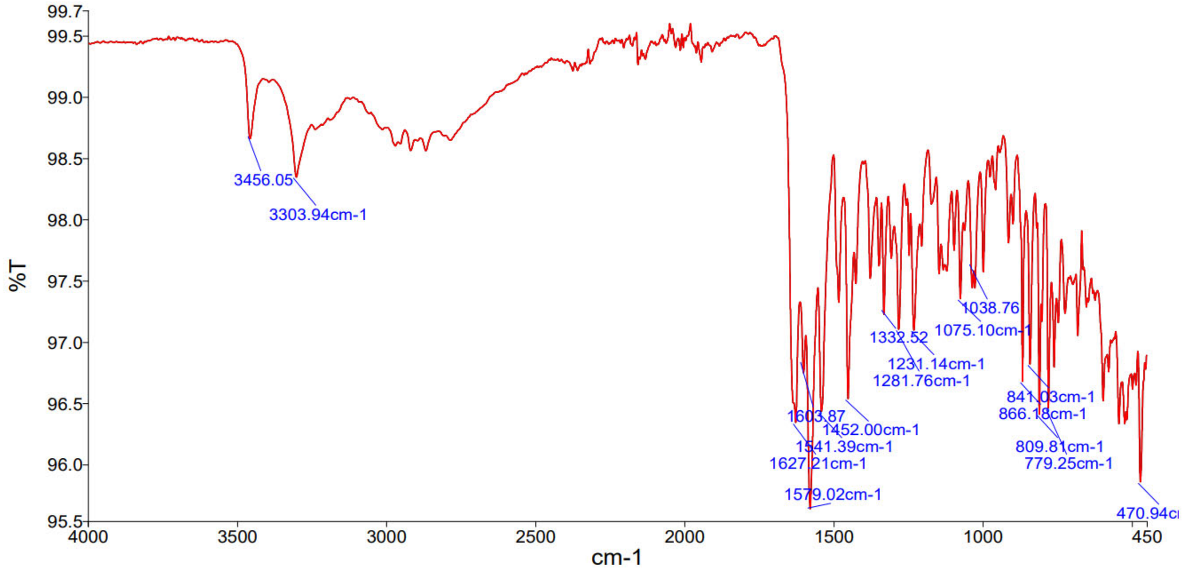
%T
cm-1
3456.05
3303.94cm-1
1603.87
1452.00cm-1
1541.39cm-1
1627.21cm-1
1579.02cm-1
1332.52
1231.14cm-1
1281.76cm-1
1038.76
1075.10cm-1
841.03cm-1
866.18cm-1
809.81cm-1
779.25cm-1

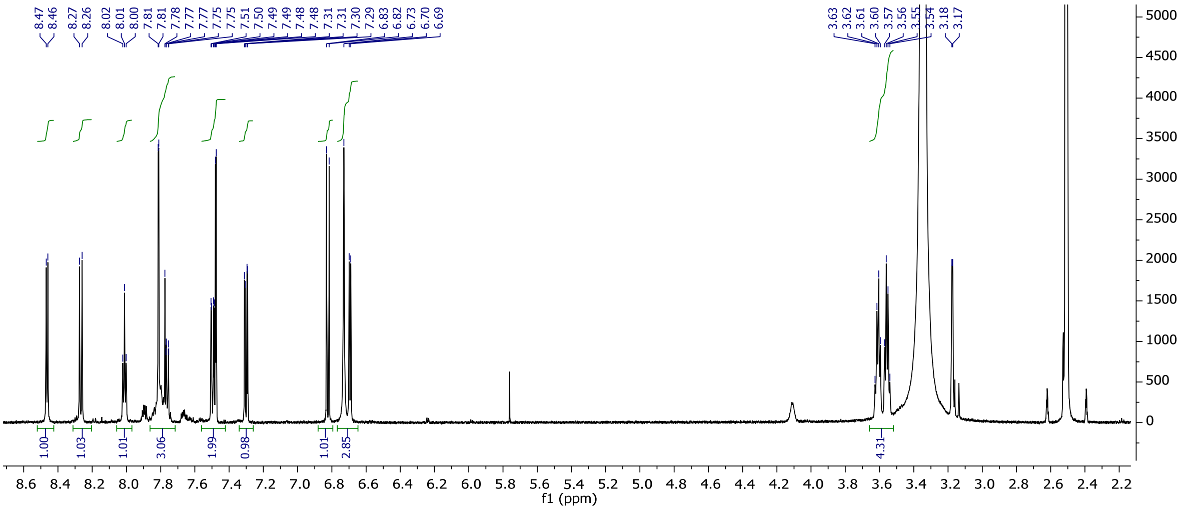
f1 (ppm)

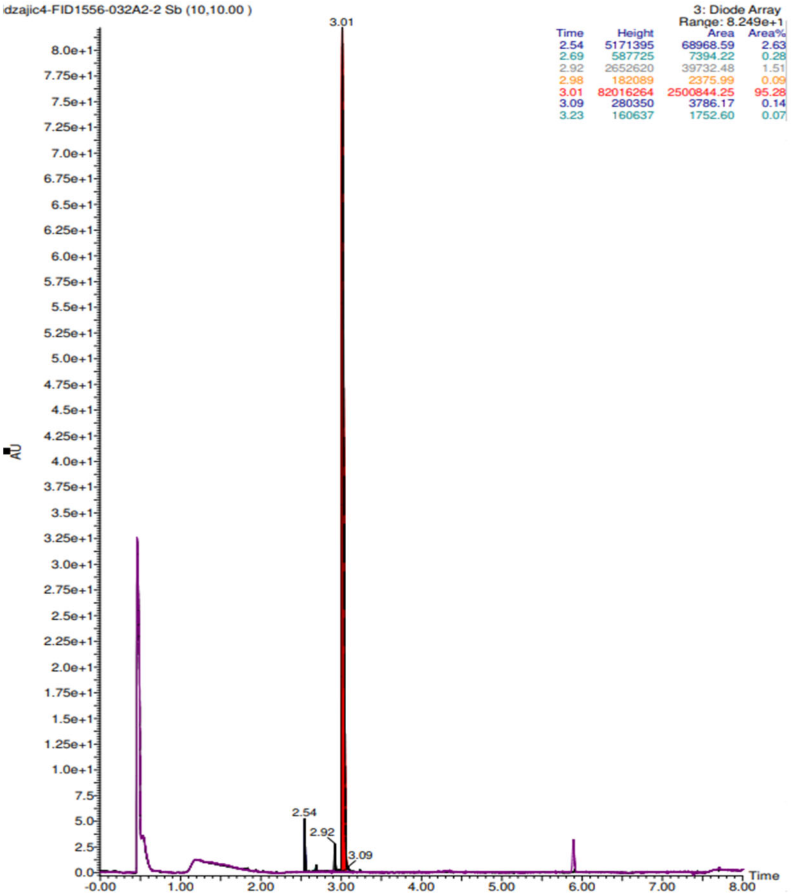

Peak ID	Compound	Time	Mass Found
5		3.05	

SAMPLE: 1:6 Combine (357:369-(339:344+382:387))

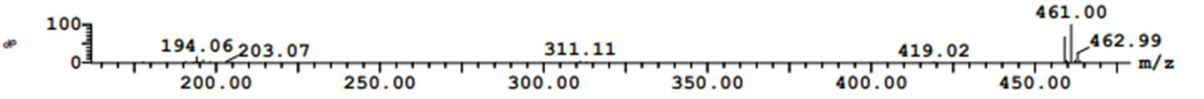

20

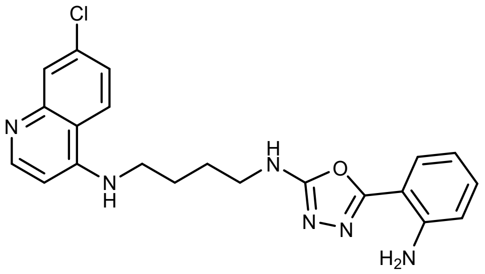

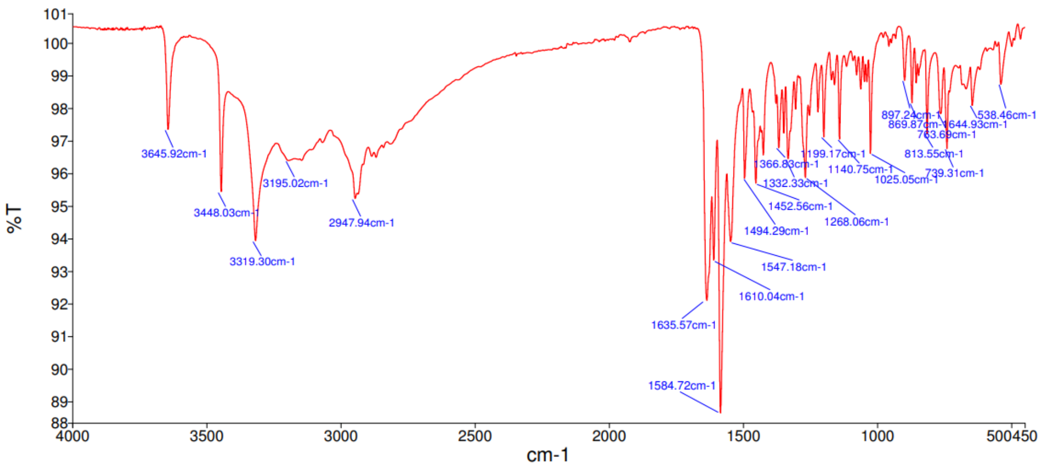

Spectrum Source	Fragmentor Voltage	Collision Energy	Ionization Mode
Peak (1) in "+/- TIC Scan"		0	ESI

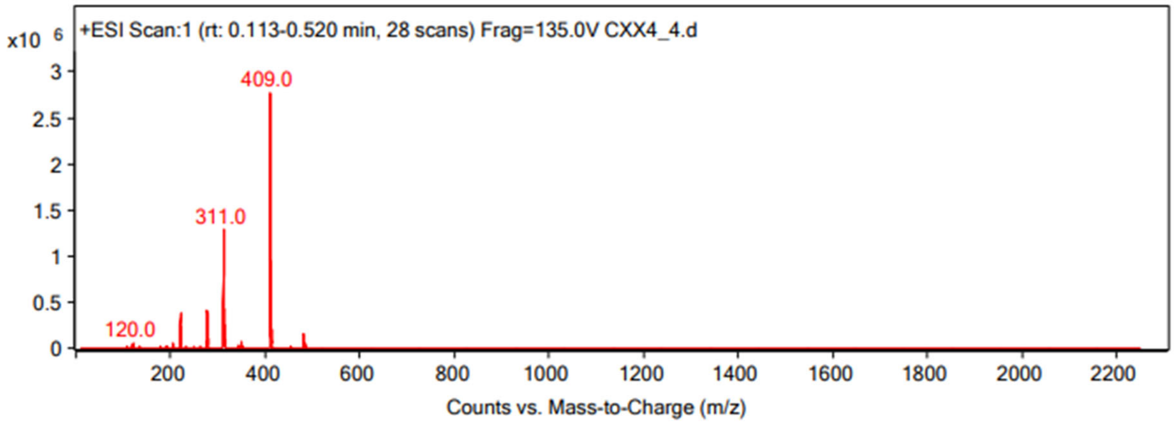

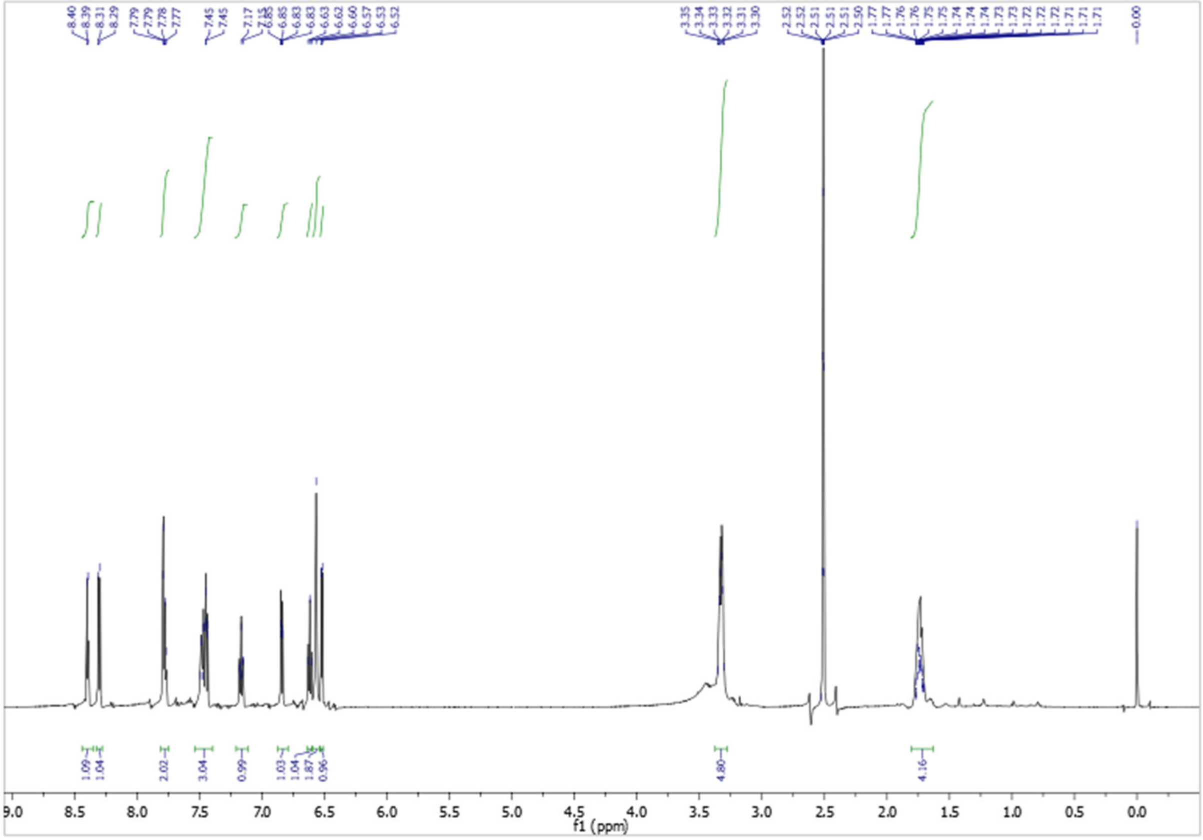
8.40
8.39
8.31
8.29
7.79
7.79
7.78
7.77
7.45
7.45
7.17
7.15
6.85
6.85
6.83
6.83
6.63
6.62
6.60
6.57
6.53
6.52
3.35
3.34
3.33
3.32
3.31
3.30
2.52
2.52
2.51
2.51
2.51
2.50
1.77
1.77
1.76
1.76
1.75
1.75
1.74
1.74
1.74
1.73
1.73
1.72
1.72
1.72
1.71
1.71
0.00
1.09
1.04
2.02
3.04
0.99
1.03
1.04
1.87
0.96
4.80
4.16
f1 (ppm)

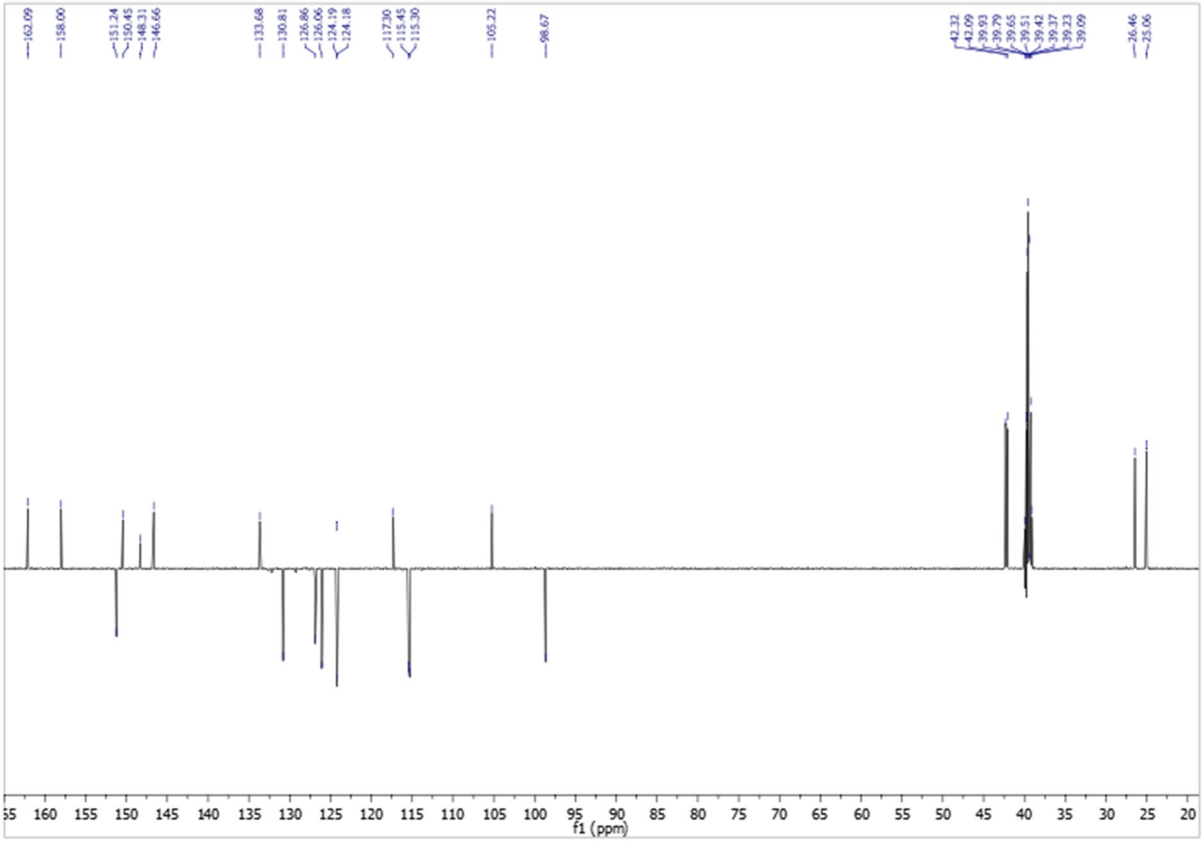
162.09
158.00
151.24
150.45
148.31
146.66
133.68
130.81
126.86
126.06
124.19
124.18
117.30
115.45
115.30
105.22
98.67
42.32
42.09
39.93
39.79
39.65
39.51
39.42
39.37
39.23
39.09
26.46
25.06
f1 (ppm)

21

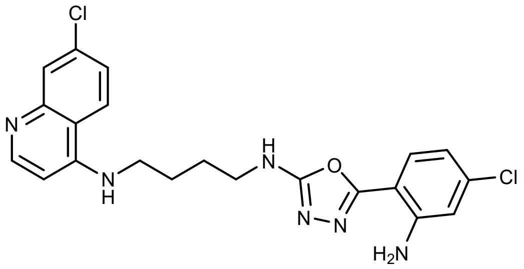

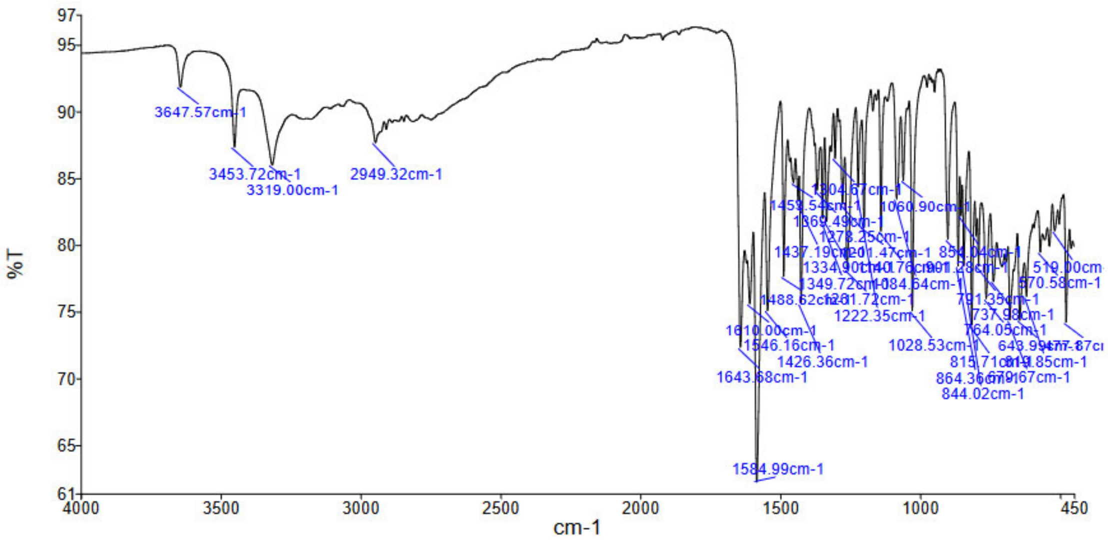

Spectrum Source	Fragmentor Voltage	Collision Energy	Ionization Mode
Peak (1) in "+/- TIC Scan"		0	ESI

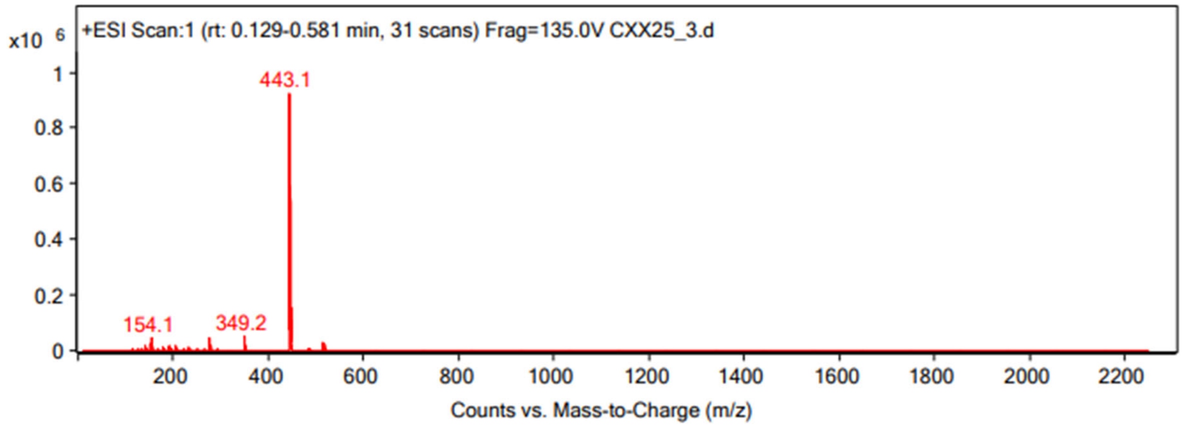

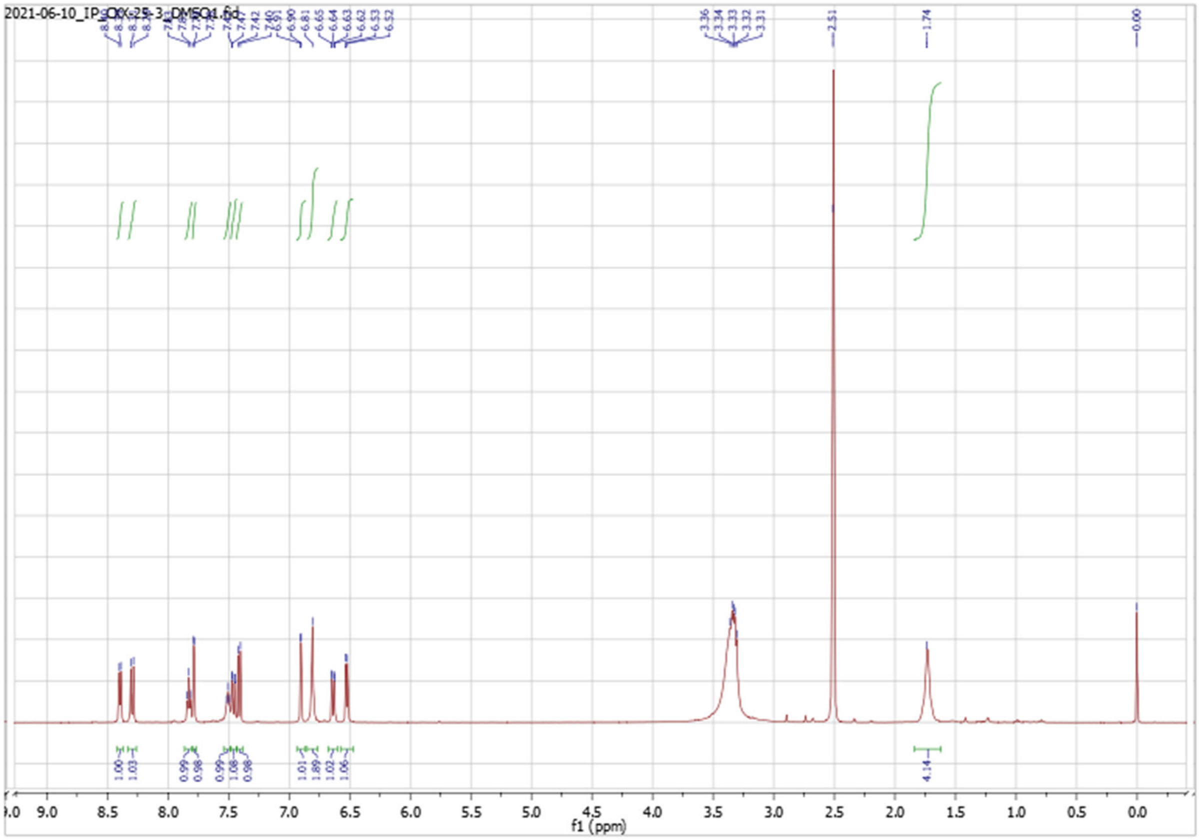
2021-06-10_IP_CXX-25-3_DMSO1.fid
f1 (ppm)

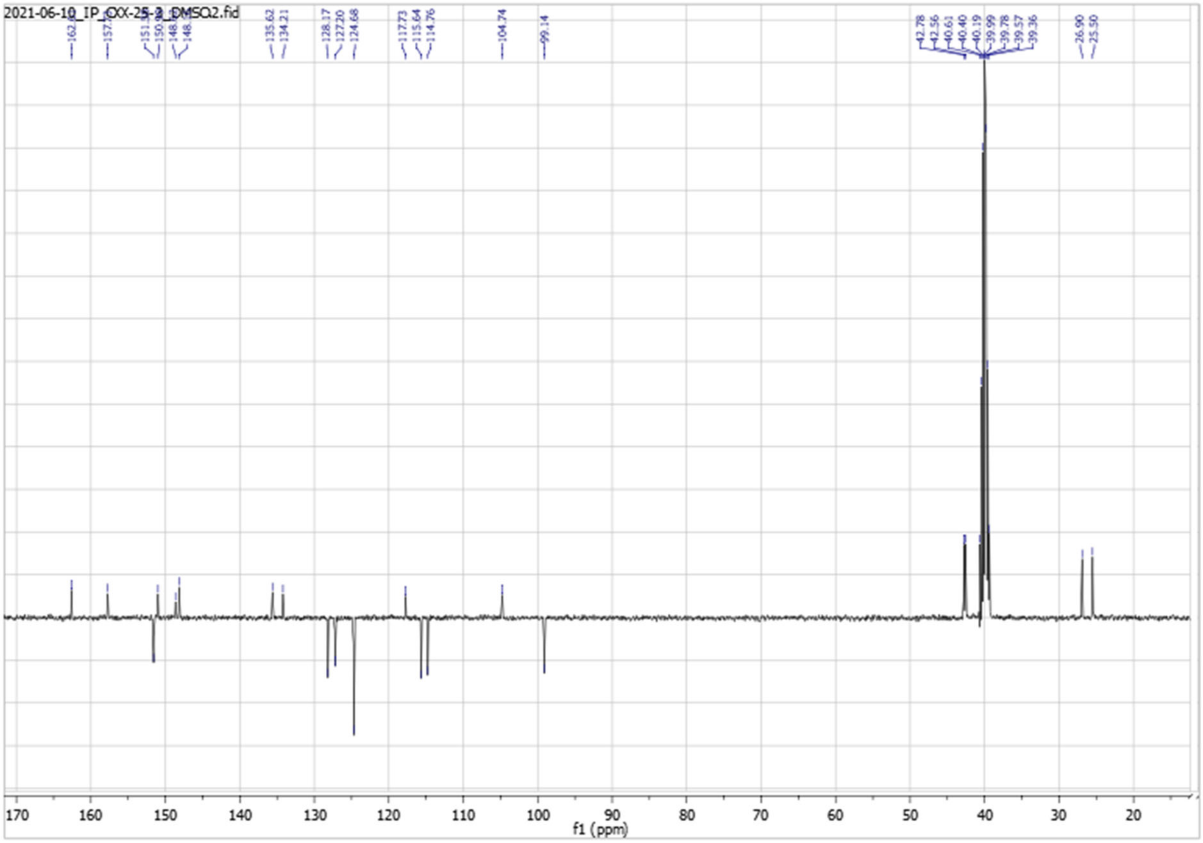
2021-06-10_IP_CXX-25-3_DMSO2.fid
f1 (ppm)

22

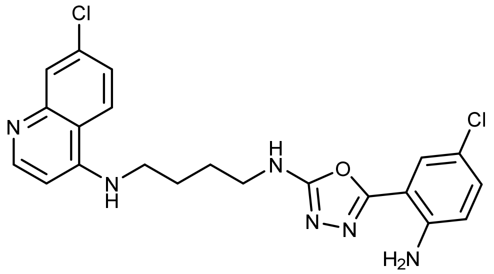
Cl
N
N
H
H
N
O
N
N
Cl
H2N

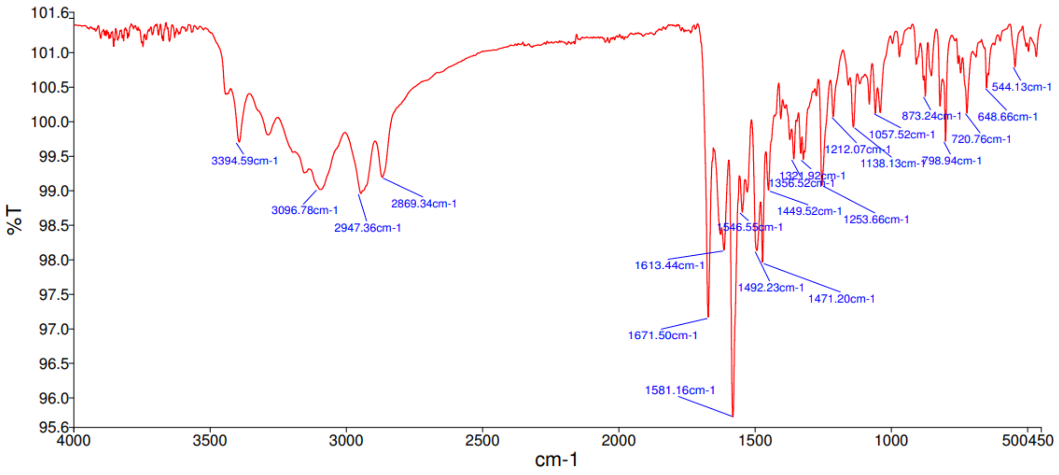
101.6
101.0
100.5
100.0
99.5
99.0
98.5
98.0
97.5
97.0
96.5
96.0
95.6
%T
4000
3500
3000
2500
2000
1500
1000
500450
cm-1
3394.59cm-1
3096.78cm-1
2947.36cm-1
2869.34cm-1
1671.50cm-1
1613.44cm-1
1581.16cm-1
1546.55cm-1
1492.23cm-1
1471.20cm-1
1449.52cm-1
1356.52cm-1
1321.92cm-1
1253.66cm-1
1212.07cm-1
1138.13cm-1
1057.52cm-1
873.24cm-1
798.94cm-1
720.76cm-1
648.66cm-1
544.13cm-1

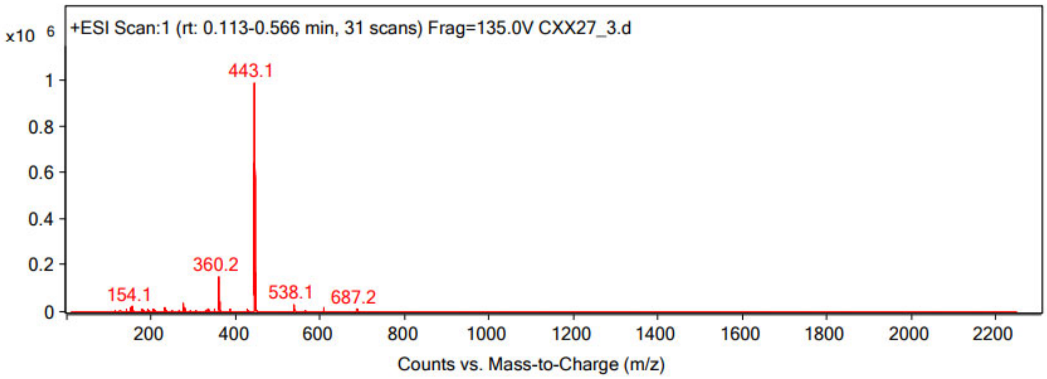
x10 6
+ESI Scan:1 (rt: 0.113-0.566 min, 31 scans) Frag=135.0V CXX27_3.d
1
0.8
0.6
0.4
0.2
0
443.1
360.2
154.1
538.1
687.2
200
400
600
800
1000
1200
1400
1600
1800
2000
2200
Counts vs. Mass-to-Charge (m/z)

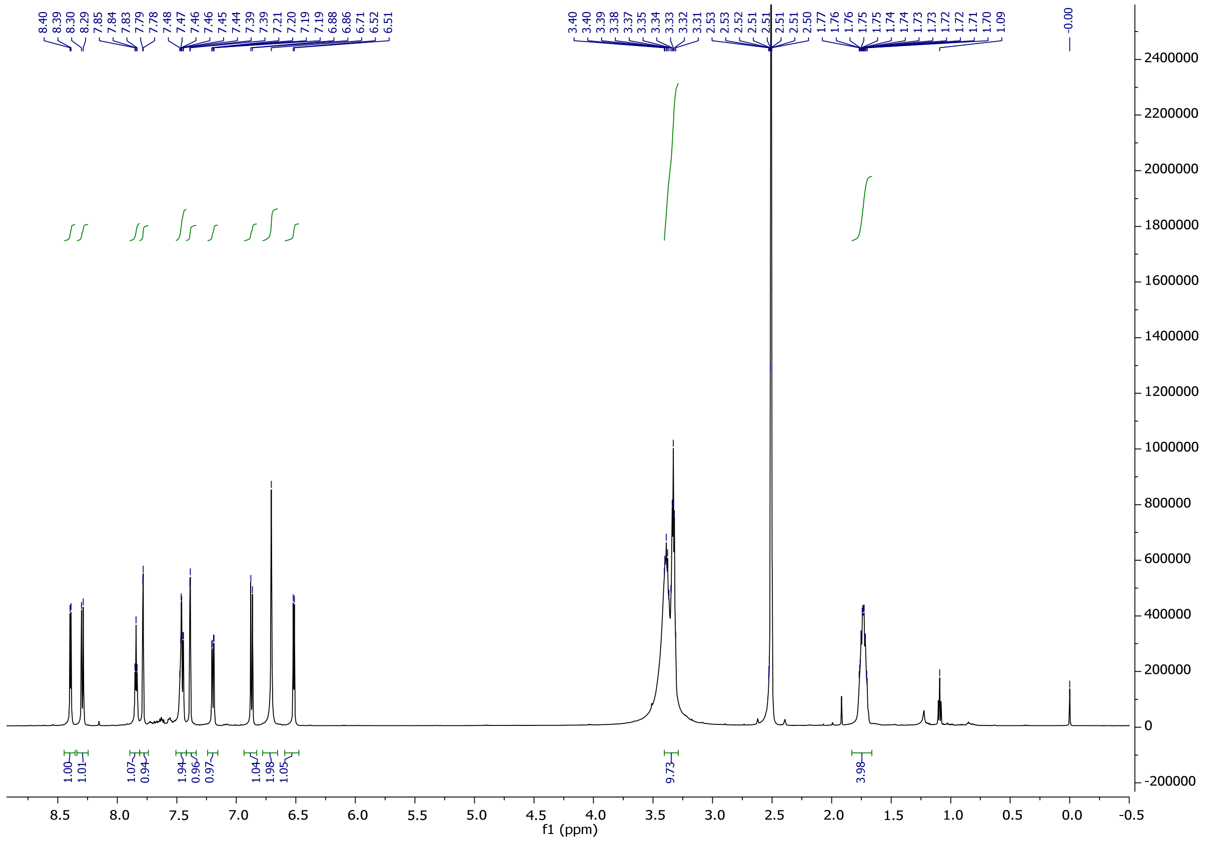
8.40 8.39 8.30 8.29 7.85 7.84 7.83 7.79 7.78 7.48 7.47 7.46 7.46 7.45 7.44 7.39 7.39 7.21 7.20 7.19 7.19 6.88 6.86 6.71 6.52 6.51
3.40 3.40 3.39 3.38 3.37 3.35 3.34 3.33 3.32 3.31 2.53 2.52 2.51 2.51 2.51 2.51 2.50 1.77 1.76 1.76 1.75 1.75 1.74 1.74 1.73 1.73 1.72 1.72 1.71 1.70 1.09
-0.00
1.00 1.01 1.07 0.94 1.94 0.96 0.97 1.04 1.98 1.05 9.73 3.98
f1 (ppm)

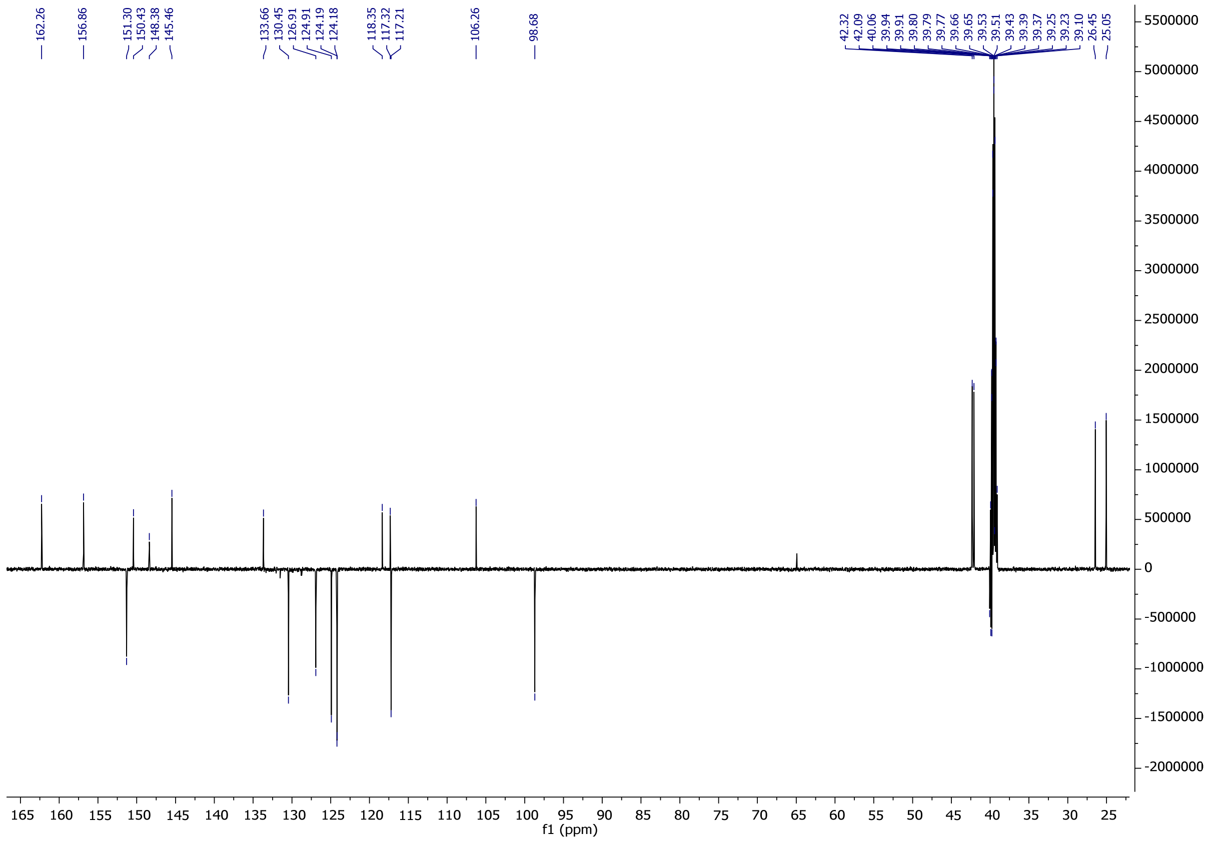
162.26 156.86 151.30 150.43 148.38 145.46 133.66 130.45 126.91 124.91 124.19 124.18 118.35 117.32 117.21 106.26 98.68
42.32 42.09 40.06 39.94 39.91 39.80 39.79 39.77 39.66 39.65 39.53 39.51 39.43 39.39 39.37 39.25 39.23 39.10 26.45 25.05
f1 (ppm)

23

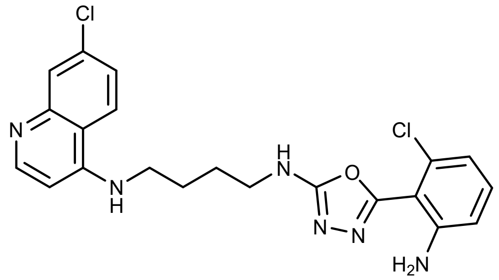
Cl
N
N
H
H
N
O
N
N
Cl
H2N

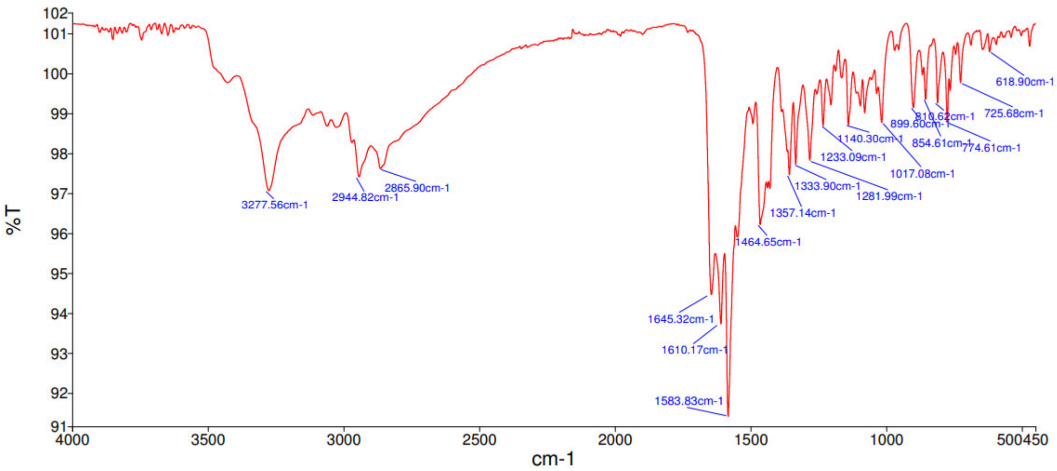
%T
cm-1
3277.56cm-1
2944.82cm-1
2865.90cm-1
1645.32cm-1
1610.17cm-1
1583.83cm-1
1464.65cm-1
1357.14cm-1
1333.90cm-1
1281.99cm-1
1233.09cm-1
1140.30cm-1
1017.08cm-1
899.60cm-1
854.61cm-1
774.61cm-1
725.68cm-1
618.90cm-1

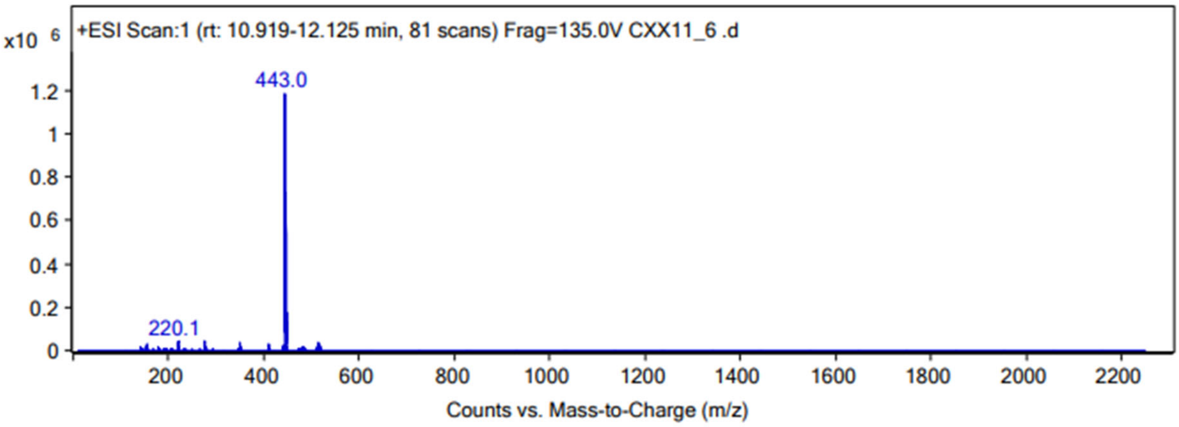
x10 6
+ESI Scan:1 (rt: 10.919-12.125 min, 81 scans) Frag=135.0V CXX11_6 .d
443.0
220.1
Counts vs. Mass-to-Charge (m/z)

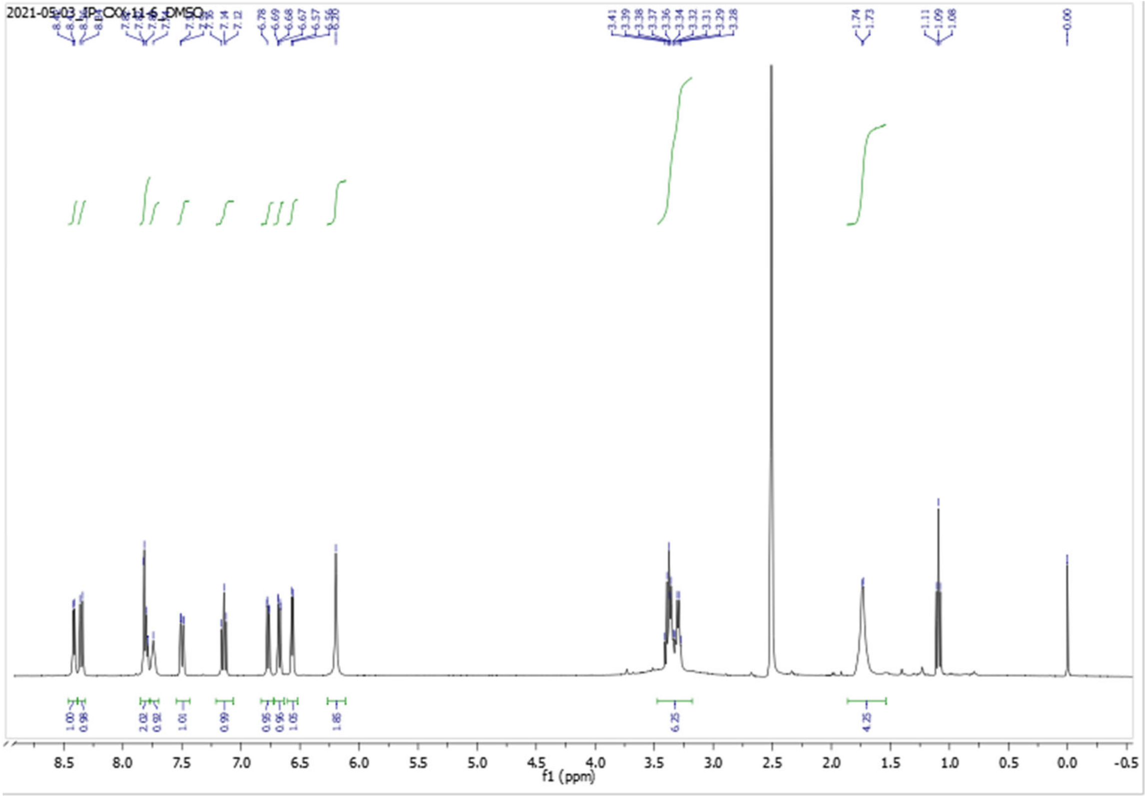
2021-05-03_IP_OX-11-6_DMSO
f1 (ppm)

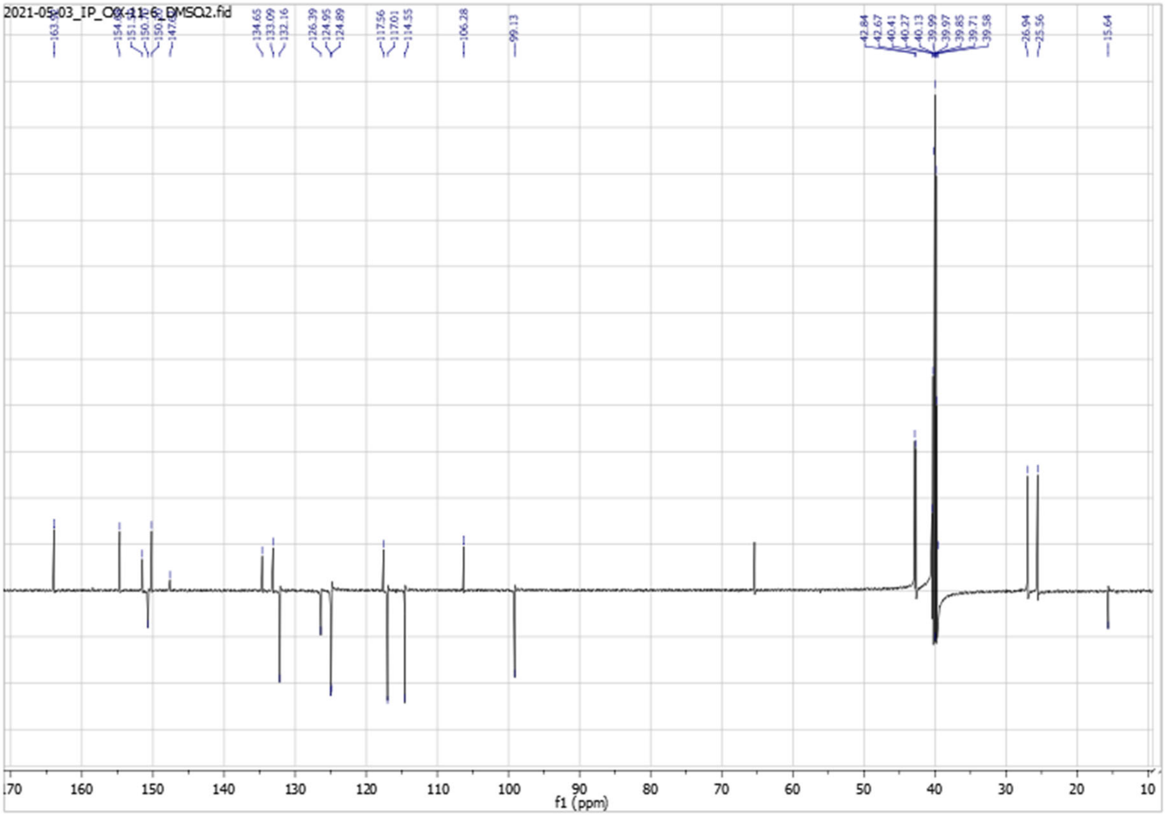
2021-05-03_IP_OX-11-6_DMSO2.fid
f1 (ppm)
